# Neoantigen-specific CD8 T cells with high structural avidity preferentially reside in and eliminate tumors

**DOI:** 10.1038/s41467-023-38946-z

**Published:** 2023-06-06

**Authors:** Julien Schmidt, Johanna Chiffelle, Marta A. S. Perez, Morgane Magnin, Sara Bobisse, Marion Arnaud, Raphael Genolet, Julien Cesbron, David Barras, Blanca Navarro Rodrigo, Fabrizio Benedetti, Alexandra Michel, Lise Queiroz, Petra Baumgaertner, Philippe Guillaume, Michael Hebeisen, Olivier Michielin, Tu Nguyen-Ngoc, Florian Huber, Melita Irving, Stéphanie Tissot-Renaud, Brian J. Stevenson, Sylvie Rusakiewicz, Denarda Dangaj Laniti, Michal Bassani-Sternberg, Nathalie Rufer, David Gfeller, Lana E. Kandalaft, Daniel E. Speiser, Vincent Zoete, George Coukos, Alexandre Harari

**Affiliations:** 1 Ludwig Institute for Cancer Research, Lausanne University Hospital (CHUV) and University of Lausanne (UNIL), Agora Cancer Research Center, Lausanne, Switzerland; 2 Center for Cell Therapy, Department of Oncology, Lausanne University Hospital, Lausanne, Switzerland; 3 Center of Experimental Therapeutics, Department of Oncology, Lausanne University Hospital (CHUV), Lausanne, Switzerland; 4 Swiss Institute of Bioinformatics (SIB), Lausanne, Switzerland; 5 Department of Oncology, Lausanne University Hospital, Lausanne, Switzerland

**Keywords:** Tumour immunology, Immunotherapy, Tumour immunology, Cytotoxic T cells, Immunological surveillance

## Abstract

The success of cancer immunotherapy depends in part on the strength of antigen recognition by T cells. Here, we characterize the T cell receptor (TCR) functional (antigen sensitivity) and structural (monomeric pMHC-TCR off-rates) avidities of 371 CD8 T cell clones specific for neoantigens, tumor-associated antigens (TAAs) or viral antigens isolated from tumors or blood of patients and healthy donors. T cells from tumors exhibit stronger functional and structural avidity than their blood counterparts. Relative to TAA, neoantigen-specific T cells are of higher structural avidity and, consistently, are preferentially detected in tumors. Effective tumor infiltration in mice models is associated with high structural avidity and CXCR3 expression. Based on TCR biophysicochemical properties, we derive and apply an in silico model predicting TCR structural avidity and validate the enrichment in high avidity T cells in patients’ tumors. These observations indicate a direct relationship between neoantigen recognition, T cell functionality and tumor infiltration. These results delineate a rational approach to identify potent T cells for personalized cancer immunotherapy.

## Abstract (web summary)

Tumor neoantigens versus tumor-associated antigens may have different functions in antitumor immunity depending on the strength of antigen recognition. Here the authors characterize CD8 T cell clones specific for TAA, neoantigens or viral antigens isolated from tumor and blood and show that neoantigen-specific clones have a higher structural avidity than TAA-specific ones and preferentially infiltrate tumors.

## Introduction

Tumor neoantigen recognition is likely a major factor in the success of clinical immunotherapies^1–5^ and patients with high tumor mutational burden (TMB)^6^ and significant number of neoantigens benefit more from immune checkpoint blockade as well as adoptive cell therapy (ACT) using natural tumor-infiltrating lymphocytes (TILs)^4, 7^. TIL therapy also showed success in low TMB cancers^8–11^. Unlike T cell clones that recognize TAAs, which are self-antigens, those recognizing neoantigens are presumably not subject to negative thymic selection^12^. Consequently, neoantigen-specific T cells may be of higher functionality^13^ and their superior antigen sensitivity was recently demonstrated^14^. T cell functionality is partly determined by the avidity of TCRs for their cognate pMHC. Because this is dictated by structure, it is referred to as structural avidity and it is determined through the dissociation kinetic of monomeric pMHCs and TCRs^15^. Conversely, sensitivity to antigen reflects the properties of the TCR but also the functional state of the cells, and is thus referred to as functional avidity^16^. Studies in mice and humans indicate that structural and functional avidities of CD8 T cells correlate^15^ and determine T cells performance^17^.

In TIL-ACT, clinical efficacy has been correlated with the persistence of adoptively transferred TIL clones in vivo^4,18^, linked to unique gene expression patterns^19^. However, how avidity affects tumor engraftment of tumor-specific T cells is presently not well understood. Yet, this is a key parameter affecting the success of T cell-based immunotherapy.

In this work, we evaluate broadly the structural avidities and antigen sensitivities of neoantigen-specific T cells and ask whether these correlate with the cell aptitude for tumor infiltration and homing. We profile a large library of CD8 T cells specific for neoantigens, tumor-associated antigens and virus epitopes from tumors and peripheral blood from healthy donors and patients with melanoma, ovarian, lung or colorectal cancer. Although neoantigen-specific T cells exhibit superior avidity than TAA-specific cells as expected, a wide range of avidities is observed. High clonotype avidity is specifically associated with tumor residence at steady state, higher CXCR3 expression and tumor engraftment following ACT in mice. Finally, we show that high-avidity TCRs share biophysicochemical properties and this allows us to generate an in silico predictor of TCR avidity. We imply a direct relationship between the strength of antigen recognition, CXCR3 expression and tumor infiltration, and provide a functional parameter for screening neoantigen-specific T cells for ACT.

## Results

### Neoantigen-specific CD8 T cells are structurally and functionally heterogeneous

**Fig. 1:**
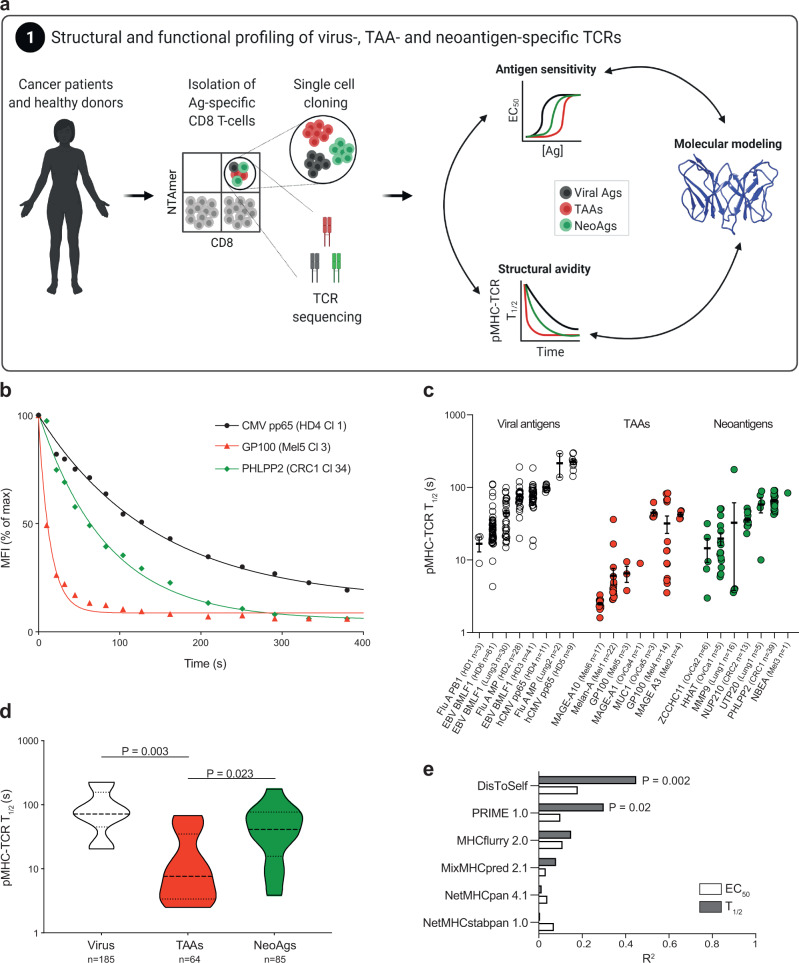
Structural avidity of neoantigen-, TAA- and virus-specific CD8 T cells. **a** Neoantigen- and TAA-specific CD8 T cells were purified from in vitro expanded CD8 T cells of melanoma, ovarian, lung or colorectal cancer patients and virus-specific CD8 were isolated from healthy donors and cancer patients. After single-cell cloning and expansion, individual clones were subjected to antigen sensitivity and structural avidity measurements as well as TCR sequencing. Molecular modeling of pMHC-TCR interactions were also performed. **b** Representative examples of structural avidity of virus-, TAA- and neoantigen-specific CD8 T cells. Structural avidity was determined with reversible pMHC multimers to measure monomeric pMHC-TCR *T*_1/2_. **c** Structural avidity of individual virus-, TAA- or neoantigen-specific CD8 T cells (mean ± SEM). The number of clones is indicated in brackets (each clone was tested individually). **d** Cumulative structural avidities per classes of antigen- (virus, TAAs and neoantigen)-specific CD8 T cells. The number of clones is indicated in brackets. *P* values are provided when significant at 95% confidence interval and using two-sided Mann–Whitney test. **e** Coefficient of determination *R*^2^ of the regression analyses (Supplementary Fig. 4a–f) between pMHC binding/stability and immunogenicity predictors values and the medians of *T*_1/2_ (s) or EC_50_ (M) of antigen-specific CD8 T cells. Pearson coefficients (two-sided test) were calculated and mentioned when significant. Patients and clones are described in Supplementary Tables 1 and 2. Source data are provided as a Source Data file.

Neoepitopes are generally considered as prototypical tumor rejection antigens. Yet, it remains unclear whether their clinical relevance stems from their tumor specificity alone or whether they truly drive better effector T cells relative to TAAs. To learn more, we generated a library of 371 CD8 T cell clones recognizing 19 neoantigens, TAAs and virus epitopes (Supplementary Table 1) from 16 patients with melanoma, ovarian, lung or colorectal cancer and 6 healthy donors (Supplementary Table 2), and investigated the functional and structural profiles of their TCRs in 190 and 338 clones, respectively (Fig. 1a). Antigen-specific cells were sorted using double-fluorescent reversible pMHC multimers (i.e. NTAmers), which avoid the selective loss of high-avidity cells^20^.

We first assessed T cell structural avidity, intended as the strength of TCR binding to cognate pMHC. This was determined through the dissociation kinetic (pMHC-TCR half-life, *T*_1/2_) of monomeric pMHCs and TCRs, as we described previously^15^. Briefly, rapid decay of reversible pMHC multimers to pMHC monomers allows dissociation rate measurements of fluorescent monomeric pMHC off CD8 T cells. We detected polyclonal responses against individual epitopes of any class in most patients or donors, with marked variance of *T*_1/2_ among clones recognizing the same epitope in each class of antigen specificity (Fig. 1b, c). Overall, the structural avidities of neoantigen-specific CD8 T cells were higher than that of TAA-specific CD8 T cells (Fig. 1d). Similar conclusions were drawn when we examined exclusively HLA-A*0201-restricted CD8 T cells (Supplementary Fig. 1a) or unique CDR3 sequences (Supplementary Fig. 1b-c and Supplementary Table 1). This supports the long-proposed hypothesis that neoepitope-specific TCRs are of higher structural avidity than T cells directed against “self” tumor antigens^21,22^.

We next assessed the antigen sensitivity of each clone by IFNγ ELISpot, measuring the peptide concentration required for half-maximal T cell activation (effect concentration 50%, EC_50_, Supplementary Fig. 2a, b). Similar to pMHC-TCR *T*_1/2_, we observed an important variance in EC_50_ among different clones recognizing the same peptide for each class of antigens, including for HLA-A*0201-restricted T cell responses (Supplementary Fig. 2c–e) or genetically unique clonotypes (Supplementary Fig. 2f, g). As expected, a positive correlation was observed between *T*_1/2_ and EC_50_, despite some variability (Supplementary Fig. 3a). We attribute the latter to more reproducible and reliable measurements obtained by pMHC-TCR dissociation kinetics when compared to functional assays, which also depend on T cell intrinsic regulatory mechanisms^17,23^. To further test the reproducibility of the structural avidity parameter, we cloned six TCRαβ chain pairs into healthy peripheral blood T cells; measurements of structural avidity remained more consistent between original and recipient T cells, maintaining similar ranking between clones, as opposed to antigen sensitivity (Supplementary Fig. 3b–e and Supplementary Material 1). This supports the robustness of structural avidity as a biophysical parameter to profile T cells.

We used an in vitro pMHC refolding assay^24^ to validate the predicted affinity of each peptide for the cognate HLA allele. The overall ranges of pMHC affinity ruled out any important bias in measurements of antigen sensitivity due to low peptide-MHC interactions. Highlighting the limitation of commonly used algorithms for predicting epitope immunogenicity, we found poor correlations between measured structural avidity (or antigen sensitivity) with in silico predictors of pMHC affinity, stability or processing, mainly relying on the determination of antigen presentation (Fig. 1e and Supplementary Fig. 4)^25–28^. However, structural avidity was significantly correlated with immunogenicity predicted by PRIME^29^ and with pMHC Dissimilarity-to-Self (DisToSelf)^30^ (Fig. 1e), also significant when viral epitopes were excluded (Supplementary Fig. 4a–f) and when only genetically unique clonotypes were considered (Supplementary Fig. 4g). PRIME not only considers the binding capacity of a peptide to a given MHC but also integrates its propensity to be recognized by TCRs^29^. DisToSelf determines the similarity (or dissimilarity) of a given peptide with the human proteome^30^. Peptides with high DisToSelf scores are recognized by higher avidity T cells (Fig. 1e and Supplementary Fig. 4g).

### High structural avidity neoantigen-specific CD8 T cells reside in tumors

Given the unveiled heterogeneity of TCR avidities for given tumor epitopes, we asked whether TCR strength discriminates cells with a propensity for tumor infiltration. Indeed, if higher avidity cells were to carry an antitumor response, they would be expected to be rather enriched in the tumor microenvironment^31,32^. Strikingly, TILs recognizing neoantigen- or TAA-epitope exhibited significantly superior antigen sensitivity relative to cognate peripheral blood lymphocytes (PBLs) recognizing the same epitope across melanoma, ovarian, colorectal and lung cancer patients (Supplementary Figs. 5 and 6).

**Fig. 2:**
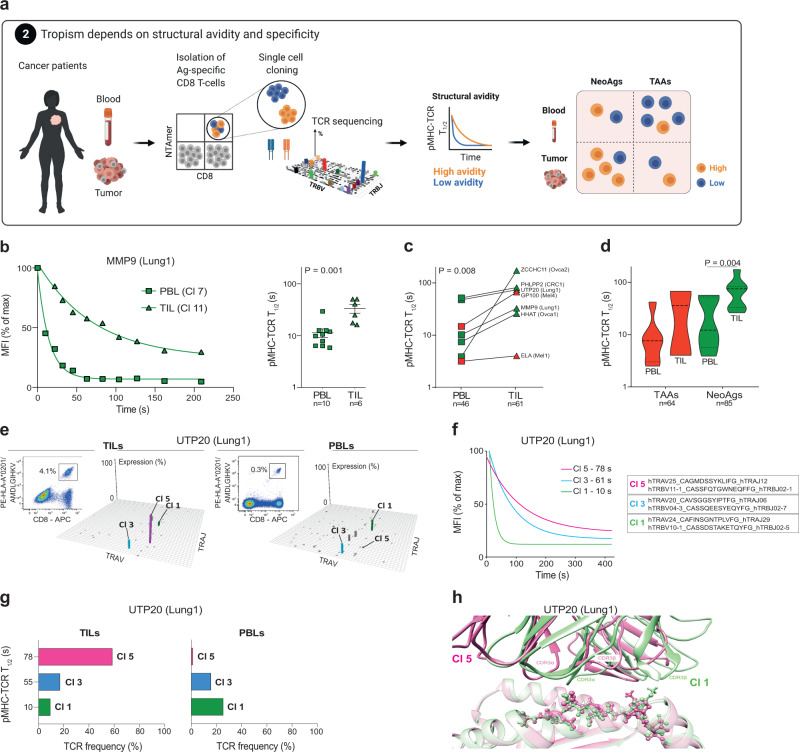
Association between structural avidity and tumor tropism. **a** Correlation between the structural profile of antigen-specific CD8 T cells and tropism at steady state. **b** Representative examples and cumulative analyses (Mean ± SEM) of monomeric pMHC-TCR dissociation kinetics of MMP9-specific PBLs and TILs assessed with NTAmers (reversible pMHC multimers). The number of clones is indicated in brackets (each clone was tested individually). *P* values are provided at 95% confidence interval and using two-sided Mann–Whitney test. **c** Comparison of the structural avidity of seven pairs of PBLs and TILs recognizing the same pMHCs. The number of clones is indicated in brackets (each clone was tested individually). Wilcoxon two-sided test was used to determine the *P* value. **d** Structural avidity of TAA- and neoantigen-specific PBLs and TILs. The number of clones is indicated in brackets. *P* values are provided at 95% confidence interval and using two-sided Mann–Whitney test when significant. **e** UTP20-specific CD8 T cells from patient Lung1 were sorted from TILs and PBLs using NTAmers, bulk TCR sequenced and cloned by limiting dilution. The Manhattan plots of TCRα repertoires are shown and only clonotypes identified in both PBLs and TILs repertoires are color-coded. **f** Monomeric pMHC-TCR dissociation kinetics of three UTP20-specific clones of patient Lung1 assessed with reversible pMHC multimers (NTAmers). **g** Relative frequency of clones 1, 3 and 5 among UTP20-specific CD8 TILs (left) and PBLs (right). Structural avidity for each clone is also plotted. **h** Superimposition of in silico analyses of the pMHC-TCRs molecular interactions for UTP20-specific clones 5 and 1. Pink and green are used to color TCR ribbons, MHC (shaded color) and peptides (ball and stick) for clones 5 and 1, respectively. Source data are provided as a Source Data file.

To assess whether differences in antigen sensitivity could be attributed to structural avidity attributes of TIL vs. PBL clones (Fig. 2a), we analyzed seven pairs of tumor-specific T cells originating from TILs or PBLs. We found that the structural avidity of TILs was significantly higher than that of cognate PBLs across all studied cancers (Fig. 2b, c and Supplementary Fig. 6a, b). Thus, antigen-specific T cells infiltrating tumors, particularly neoantigen-specific clones, display stronger structural avidity than their blood counterparts, including when genetically unique clonotypes are considered (Fig. 2d and Supplementary Fig. 6c, d).

To better understand the relative enrichment of TILs in high-avidity cells, we sequenced the TCRs of sorted primary CD8 PBLs and TILs recognizing the same neoepitope from the UTP20 protein from patient Lung1. Neoantigen-specific T cells were oligoclonal, but only three TCRs were shared between PBLs and TILs (Fig. 2e). Remarkably, clonotype 5, which was dominant in TILs (58.8% of neoepitope-specific cells), was only contributing to 1.4% of the PBL repertoire, while clonotype 1 was less frequent in TILs (9.6%) but dominant in PBLs (26.1%), and clonotype 3 showed similar frequency in TILs (17.2%) and in PBLs (16.2%). Interestingly, the structural avidity of UTP20-specific TCRs (Fig. 2f) correlated with their frequency in the tumor compartment, and was the highest for clonotype 5, indicating that tumor-resident clones have higher structural avidity (Fig. 2g). We previously showed^31^ that molecular modeling of TCR and pMHC can accurately infer the strength of their interaction. Here we confirmed that clone 5 TCR established significantly more favorable interactions with UTP20 pMHC than clone 1 TCR (Fig. 2h and Supplementary Table 3). Similar results were obtained in a second example in patient CRC1 for PHLPP2-specific TCRs confirming the association between structural avidity and tumor residence (Supplementary Fig. 7a–d). These observations indicate that the preferential accumulation of high and low-avidity clones in tumors and blood, respectively, is also true among clonotypes from the same antigen-specific repertoires.

**Fig. 3:**
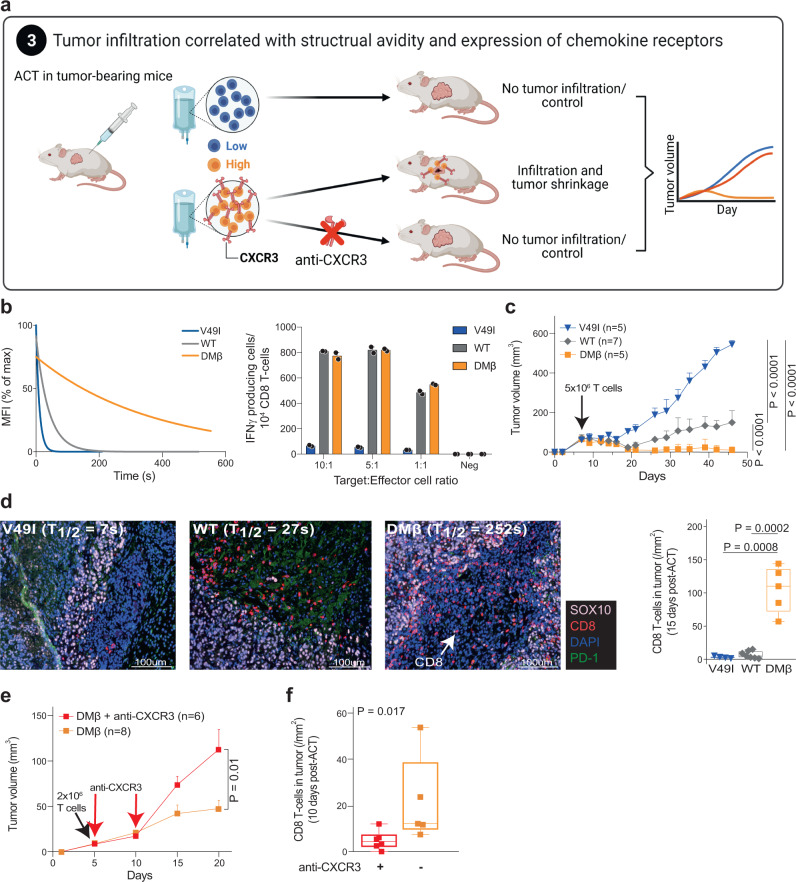
Tumor infiltration of high-avidity clones is associated to CXCR3 expression. **a** Preferential tumor infiltration by high-avidity clones and CXCR3-mediated tumor homing was validated by in vivo ACT in mice and in four melanoma patients receiving T cell therapy. **b** Reactivity of V49I, WT and DMβ-transduced T cell was measured through IFN-γ secretion upon coculture with Me275 tumor cells (right, *n* = 2 independent experiments, Mean ± SEM) and monomeric pMHC-TCR dissociation kinetics of the three mutants were determined using reversible pMHC multimers (left, *n* = 2 independent experiments). **c** Me275 tumor growth in IL-2 NOG mice adoptively transferred at day 7 post-tumor engraftment with 5 × 10^6^ primary CD8 T cells transduced with V49I, WT or DMβ NY-ESO-I_157–165_-specific TCRs (*n* = 1 independent experiment, Mean ± SEM). Log-rank two-sided tests were used to determine *P* values. **d** Representative examples of Me275 tumor sections, harvested from engrafted IL-2 NOG mice at day 8 post-ACT with 5 × 10^6^ V49I, WT or DMβ-transduced T cells (*n* = 2 independent experiments). Tumors were stained for SOX10, PD-1 and CD8. DAPI was used to stain nuclei. For tumor infiltration by CD8 T cells (cells/mm^2^), bounds of box are 25^th^ to 75^th^ percentiles with median, whiskers are min to max. Mann–Whitney two-sided test was used to calculate *P* values. Analyses were performed using Inform v2.3.0^62^. **e** Me275 tumor growth in IL-2 NOG mice adoptively transferred with 2 × 10^6^ DMβ-transduced primary CD8 T cells at day 5 and co-injected or not with anti-CXCR3 blocking antibody (100 μg at day 5 and day 10) (*n* = 2 independent experiments, Mean ± SEM). Log-rank test was used to determine *P* value. **f** Quantitative measurement of tumor infiltration by CD8 T cells (cells/mm^2^) 10 days post-ACT of 2 × 10^6^ DMβ-transduced primary CD8 T cells co-injected or not with anti-CXCR3 blocking antibody (*n* = 2 independent experiments). Bounds of box are 25^th^ to 75^th^ percentiles with median, whiskers are min to max. Mann–Whitney two-sided test was used to calculate the *P* value. Source data are provided as a Source Data file.

To experimentally validate the preferential tumor infiltration by high structural avidity T cells (Fig. 3a), we took advantage of a well-characterized panel of NY-ESO-1_157_–_165_-specific TCRs with high (DMβ), intermediate (WT) and low (V49I) structural avidity. Their avidity covers the range of viral-, neoantigen- and TAA-specific T cells^33–35^. We stably transduced CD8 T cells of an HLA-A*0201 donor with DMβ, WT or V49I TCRs and profiled their structural and functional avidities (Fig. 3b). Unlike V49I-transduced T cells, both WT and DMβ variants showed equivalent in vitro responsiveness to HLA-matched Me275 melanoma tumor expressing NY-ESO-1 (Fig. 3b). ACT of 5 × 10^6^ T cells in interleukin-2 (IL-2) NOG mice bearing Me275 tumors indicated a correlation between the in vivo efficacy and the structural but not the functional avidity of TCR-transduced T cells (Fig. 3c). Following ACT, DMβ-transduced CD8 T cells significantly better infiltrated tumors as compared to V49I- and WT-transduced cells (Fig. 3d), confirming higher engraftment propensity of high-avidity clones.

### CXCR3-mediated tumor infiltration and control by high-avidity T cells

Having established a relationship between T cell avidity and tumor homing, we hypothesized that high-avidity cells may be endowed with a superior ability for tumor infiltration and retention (Fig. 3a). Several studies reported that key chemokine receptors, especially CXCR3, may be required for tumor homing^36^. We analyzed the expression of a panel of chemokine receptors on seven pairs of low and high-avidity antigen-specific CD8 T cells. CXCR3 was more strongly expressed and upregulated after short-term stimulation by high as compared to low-avidity T cell clones (Supplementary Fig. 8a). This observation was specific to tumor homing-related molecules since no significant difference was found for chemokine receptors that are not specifically involved in tumor infiltration (e.g. CCR7). In addition to CXCR3, CD103 and CD49a (VLA-1), two major integrins associated with a tissue-residency phenotype^37–39^, were both upregulated in high-avidity clones (Supplementary Fig. 8b). Therefore, T cell structural avidity is associated to CXCR3 expression and, to a lower extend, to CD103 and CD49a expression.

Higher CXCR3 expression was also observed on DMβ- relative to V49I- or WT-transduced T cells upon pMHC stimulation in vitro (Supplementary Fig. 8c). Of interest, addition of an anti-CXCR3 antibody after ACT in IL-2 NOG mice bearing the Me275 melanoma tumor, known to express CXCR3 ligands i.e. CXCL9/10/11^40^, with DMβ-transduced T cells (Fig. 3a) significantly impaired tumor control (Fig. 3e). Consistently, lower densities of CD8 T cells were observed in animals treated with an anti-CXCR3 blocking antibody post-ACT (Fig. 3f). The inhibition of tumor control after ACT by blocking CXCR3 was further demonstrated in two additional models using neoantigen-specific TCRs (Supplementary Fig. 8d, e). This confirms the contribution of CXCR3 in tumor homing and mechanistically links CXCR3 expression with high-avidity clones and tumor infiltration.

### Biophysicochemical inference of tumor-specific T cells that engraft in tumors

**Fig. 4:**
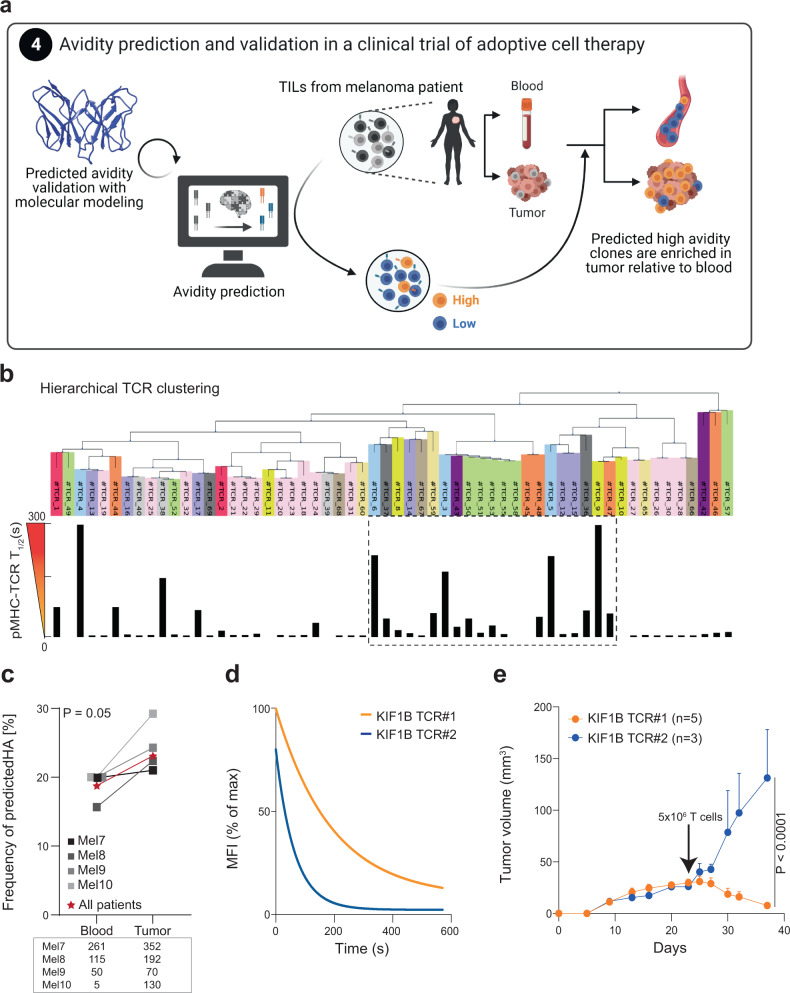
Tumor infiltration after ACT correlates with predicted structural avidity inferred from TCR clustering analyses. **a** Computational analysis of TCR features led to the establishment of a predictor of TCR avidity and its application on patients’ TIL-ACT products allowed tracking of predicted low and high-avidity TCRs in post-ACT tumor samples. **b** Hierarchical clustering of 58 TCR sequences provided based on a biophysical approach^43^. TCRs sharing the closest 4-mer features are next to each other and TCRs recognizing the same pMHC have the same color code. TCR model numbers are presented as labels and further details about TRAV, TRAJ, TRBV, TRBJ, HLA and peptide are found in Supplementary Table 4. The structural avidity of each cognate TCRs is represented below (mean of *n* = 3 independent experiments). The black dashed box highlights a region where high-avidity TCRs recognizing multiple pMHC specificities are clustering. **c** Cumulative analysis for four melanoma patients of the percentage of predicted high-avidity CD8 T cells in blood and tumor samples. Values for individual patients are plotted (gray and black) as well as the cumulative analysis (in red) for which the *P* value was calculated as described in the method section. The number of clones is indicated below for each patient individually. **d** Monomeric pMHC-TCR dissociation kinetics of Jurkat cells transfected with neoantigen KIF1B_S918F_-specific TCR#1 and TCR#2, respectively predicted as high and low-avidity TCRs. **e** Autologous (Mel8) tumor growth in IL-2 NOG mice adoptively transferred at day 22 with 5 × 10^6^ primary T cells transduced with KIF1B_S918F_-specific TCRs (*n* = 1 independent experiment, Mean ± SEM). Log-rank two-sided test was used to determine the exact *P* value. Source data are provided as a Source Data file.

The above findings collectively suggest that tumor-infiltrating lymphocytes are enriched in tumor-specific T cell clones endowed with high-avidity TCRs. Inspired by prior demonstration that differences in the antigen sensitivity of T cell clones targeting a given pMHC correlates with the strength of pMHC-TCR binding, specifically the number of atomic contacts between TCR and pMHC inferred by molecular modeling^31^, we sought to develop further methods to infer the avidity of clones for a given epitope (Fig. 4a). We used homology modeling (see methods) to compare TCRs recognizing the same pMHC with high or low structural avidity, applied to five distinct antigens. The number of favorable interactions (bonds) of each TCR with its cognate pMHC, inferred based on the modeled structures of its α and β chains and the cognate pMHC, was consistently higher for high structural avidity TCRs (Supplementary Fig. 9a and Supplementary Table 3), and significantly correlated with pMHC-TCR *T*_1/2_ (Supplementary Fig. 9b).

A major limitation in identifying clinically relevant T cells is the lack of knowledge of possible cognate antigens. To solve this, we hypothesized that high-avidity TCRs may share common sequence features (Fig. 4a). Furthermore, it has been reported that highly frequent clones among TILs may not be tumor-specific^41^. To overcome these limitations, and driven by the above molecular modeling results, we asked whether we could infer specifically high structural avidity TCRs based on their sequence analysis and without prior knowledge of their specificity. We selected 58 individual TCRs recognizing 12 distinct pMHC, for which TCR *α* and *β* sequences as well as structural avidities (Supplementary Table 4) were known, and looked for structural patterns. We used biophysical features of k-mers encoded based on the Atchley factors and a generic hierarchical clustering algorithm^42,43^. We found that CDR3β sequences in high-avidity TCRs (*T*_1/2_ > 60 s) were significantly enriched in specific amino acid residues (i.e. N, E, I, K, T, Y, V; all *P* < 0.0001 compared to low-avidity TCRs). Conversely, A, R, D, L, M and P were more frequent in low-avidity TCRs (all *P* < 0.0001) (Supplementary Fig. 10 and Supplementary Table 5).

We next developed hierarchical clustering based on CDR3β motifs and, interestingly, we identified a hotspot enriched in TCRs with high structural avidity, irrespectively of their target (Fig. 4b, dashed black box). This comprised 62% of all TCRs of intermediate or high avidity (*T*_1/2_ > 10 s), while outside of this cluster, 65% of TCRs had structural avidity <10 s. Such enrichment was not observed in a control analysis with 1000 random clustering, illustrating the significance of this observation (*P* < 0.001) and indicating that some shared common CDR3β features were preferentially associated with higher structural avidity (Supplementary Material 2).

Guided by this observation, we derived a structure-based logistic regression model to predict the structural avidity of TCRs of unknown specificity (Fig. 4a, “Methods” and Supplementary Material 3). We applied it to our panel of 58 TCRs and were able to accurately discriminate between high and low-avidity TCRs with an AUC of 0.96, with only one false-positive and one false-negative high avidity among the full set of TCRs (sensitivity of 0.91 and specificity of 0.97) (see “Methods”). Three cross-validations schemes were successfully performed on viral peptides, TAAs and neo-antigens and on different HLA alleles to assess the robustness of the predictor, following the standard leave-20%-out protocol (cross-validations 1 and 2) as well as a more challenging leave-one-epitope-out cross-validation (cross-validation 3). We achieved a success higher than 70% in all cases, which leaves room for improvement but is significantly better than random and gives us confidence in the algorithm for the purpose and data described herein (Supplementary Material 3). The relevance and the domain of applicability of this model will require further investigations and will most probably increase in time by incorporating additional experimental data. In the future, thanks to the additional experimental data that will be collected by us and the community, more CDRs and residues will be included in our predictor.

We then applied the structure-based logistic regression to identify high and low-avidity TCRs in blood and tumors of four additional melanoma patients (Supplementary Table 2). When analyzing total tumor and blood TCR repertoires, we consistently found an enrichment in TCRs predicted to be of high avidity in tumors relative to blood (Fig. 4c, *P* = 0.05), therefore validating the preferential tumor tropism for high-avidity TCRs. Furthermore, we also identified two TCRs directed against neoantigen KIF1B_S918F_ in enriched TILs^44^ from patient Mel8 (Supplementary Fig. 11a and Supplementary Table 2). Among the two KIF1B_S918F_ TCRs, and despite no differences in functional avidity (Supplementary Fig. 11b), TCR#1 was predicted by our logistic regression to be of high avidity, while TCR#2 was predicted to be of low avidity and these predictions were validated experimentally (Fig. 4d). We then took the opportunity of the availability of autologous tumor cells to assess the relative clinical efficacy of the two KIF1B_S918F_ TCRs. Despite the fact that both TCRs were tumor-reactive in vitro (Supplementary Fig. 11c), tumor control in mice bearing the autologous tumor was exclusively achieved after ACT with T cells transduced with the predicted and validated high-avidity TCR but not with the low-avidity TCR (Fig. 4e). This prototypical example illustrates the superior tumor infiltration and control by high-avidity cells, even when they target the same neoepitope but also highlights the clinical relevance of the logistic regression to predict clinically relevant TCRs regardless of their specificity.

## Discussion

Success of TIL-based immunotherapies for solid tumors relies on strong antitumoral activity of adoptively transferred T cells. Major efforts are thus made to develop methods allowing to estimate a priori the functional potential of tumor antigen-specific T cells. The strength of T cell recognition is a key parameter, as it affects T cell activation, proliferation, infiltration and effector functions, as well as longevity of T cell responses^17,45^. Besides the structural avidity of the TCR, multiple coreceptors are implicated in determining T cell functional avidity^46,47^. Cellular assays (cytotoxicity or cytokine production) have been traditionally used to determine antigen sensitivity for which EC_50_ represents a widely accepted parameter. However, cellular assay results depend on the state of cellular activation or exhaustion, limiting their performance^17^. The dissociation kinetic measurement of pMHC from the TCR, a structural avidity parameter reflecting the binding strength of a TCR^15^, can be readily applied on viable T cells. Reversible pMHC multimers can be used to reliably determine such dissociation kinetics, showing T cell functionality independently of the cellular activation state^17^.

Here we comprehensively profiled tumor antigen-specific T cells in patients with solid tumors and compared them with virus-specific cells. To do so, we generated 371 T cell clones upon FACS-sorting with reversible pMHC multimers, precluding the loss of high-avidity T cells prone to TCR-induced cell death induced by conventional pMHC multimers^20^. As expected, we found that virus-specific T cells show higher avidity and function than TAA-specific cells^48^. Neoantigen-specific T cells displayed higher structural avidity than TAA-specific T cells. The superiority of neoantigen-specific T cells over TAA-specific ones was not recapitulated by IFNγ release. Functional avidity assays largely rely on T cell activation states and are more prone to intra- and inter-experimental fluctuations^17^. T cell responses of high-avidity clonotypes can also be inhibited by exhaustion mechanisms^49^. This legitimates structural avidity as a robust and reliable biomarker of T cell responsiveness. However, a broad heterogeneity was observed in the avidity of neoantigen-specific T cells, ranging from low avidities, comparable to TAAs-, to high avidities, comparable to virus-specific cells. Also seen by others^50,51^, we found a correlation between TCR avidity and T cell potency for the same antigen but also across antigen specificities, providing the rationale for developing prioritization algorithms in TCR discovery. Of note, the strength of interaction between effector cells and cognate antigen is becoming an attractive parameter to measure T cell activation and predict efficacy^52^. Interestingly, the importance of the binding strength between effector cells and tumors was also recently demonstrated with CAR T cells^53^.

The broad heterogeneity of TCR avidities supports the notion that neoantigens may strongly differ in their potential to mediate antitumor effects in vivo. Identification of neoantigens relies on in silico prediction of antigen binding avidity to MHC molecules, with a discovery rate <5%, arguing that only a minor fraction of presented peptides are immunogenic^31,41,54^. Indeed, we did not find any correlation between functional or structural parameters and prediction of peptide binding affinities, but did so with immunogenicity prediction through PRIME^29^. This presumably reflects the importance of the mutation occurring at MHC anchor residues or directly in those in contact with the TCR, which is ultimately captured by molecular modeling^29,55^.

It has been reported that clonally expanded T cells can reside in tumor tissue and adjacent normal tissue or blood^56^. Here, by analyzing T cells from blood and tumor targeting the same tumor antigen, we found that the latter consistently show higher antigen sensitivity and structural avidity. However, while TILs are enriched for high-avidity T cell populations, common TCR clonotypes were also identified in PBLs (albeit at lower frequencies), consistently with the presence of tumor-reactive TIL clonotypes in the circulation^32^. The association between structural avidity and tumor infiltration was seen across multiple epitopes and multiple patients, but also within antigen classes (TAAs and neoantigens) and within distinct clonotypic repertoires of neoantigen-specific T cells. Furthermore, despite the fact that T cells from both blood and tumors were systematically interrogated for each patient and each antigen, neoepitopes and TAAs were preferentially detected in TILs and PBLs, respectively, consistently with the superior structural avidity of neoepitope-specific T cells. The high frequency of neoepitope-specific TCRs in tumors was recently shown in patients with lung cancer^57^.

We also showed that structural avidity is associated with CXCR3 expression, known to promote tumor infiltration^36,58^, as well as CD103 (αEβ7) and CD49a (VLA-1) expression, both associated with tumor residency^38,39^. CXCR3 blocking after ACT prevented tumor infiltration of high-avidity tumor-specific T cells. Tumor infiltration and eradication probably rely on several other complementary parameters, like T cell expansion and persistence. A major issue in ACT therapy is the downregulation of antigen presentation, which could indeed favor high-avidity T cells that are less dependent on antigen concentration. This was recently suggested in melanoma patients where TAA-related antigen expression was higher than that of neoantigen and was inversely correlated with the functional avidity of the respective antigen-specific T cells^14^.

Our unique capacity to comprehensively profile CD8 T cells by measuring structural avidity, linked to TCR biophysicochemical and sequence features, as well as structure modeling, allowed us to build a structure-based logistic regression model of TCR avidity level prediction of TCRs with unknown antigen specificity. To prevent overfitting of the model, we limited the number of parameters entering the equation and performed successfully several cross-validation tests to assess the robustness of the approach, following the standard leave-20%-out protocol as well as a more challenging leave-one-epitope-out cross-validation. Although this predictor will require further optimization—e.g. by addition of other parameters—and validation using a larger external test set when more experimental data will become available, it allowed us to identify TCRs with high-avidity features in four melanoma patients. These were found more frequently within tumors than blood at steady state, supporting the notion that high-avidity TCRs preferentially home and reside in tumors.

Our data link neoantigen recognition, T cell functionality and ability to infiltrate and reside in tumors, suggesting that the clinical relevance of neoantigen-specific T cells is not only related to their tumor specificity but also to their higher functionality and their preferential ability to infiltrate tumors. Our data also indicate that tumor-specific CD8 T cells are highly heterogeneous and that measurements of structural avidity can be used for better selection of clinically relevant T cells, avoiding the use of poorly functional clonotypes, both for TAA- and neoantigen-specific T cells. High-avidity T cells (i.e. preferentially TILs) should therefore be prioritized for personalized therapies, including TCR-based immunotherapy.

## Methods

### Ethical statement

This research complies with all relevant ethical regulations. All patient samples used in this study were collected under protocols approved by the respective institutional regulatory committees (see in the next section). All patients signed written informed consents. In vivo experiments were performed in accordance with Swiss ethical guidelines under approved licenses (see in the next section) and comply with the 3R guidelines.

### Patients and regulatory issues

Patients included stage III/IV metastatic melanoma, ovarian, non-small cell lung cancer and colorectal cancer patients (Supplementary Table 2) and had received several lines of chemotherapy and immunotherapy. Samples were collected and biobanked from patients enrolled under protocols approved by the respective institutional regulatory committees at the University of Pennsylvania, USA, and Lausanne university hospital (CHUV), Switzerland. Patients recruitment, study procedures, and blood withdrawal were approved by regulatory authorities and all patients signed written informed consents. Collection from healthy donors followed legal Swiss guidelines under the project P_123 with informed consent and with Ethics Approval from the Canton de Vaud (Switzerland). Gender was not considered in the study design as no biases are expected.

### In vivo studies

IL-2 NOG mice were obtained from Taconic Biosciences and maintained in a conventional animal facility at the University of Lausanne under specific pathogen–free status, with dark/light cycles of 12 h, humidity 55% (± 10%) and a temperature of 22 °C (±1 °C). Six- to 9-week-old female mice were used in all experiments. As only one gender could be obtained from Taconic Biosciences and we are studying ovarian cancer, only females were used. This study was approved by the Veterinary Authority of the Canton de Vaud (under the license 3387 and 3746) and performed in accordance with Swiss ethical guidelines.

### Identification of non-synonymous tumor mutations

Genomic DNA from cryopreserved tumor tissue and matched PBMC was isolated using DNeasy kit (Qiagen, cat# 4452222) and subjected to whole exome capture and paired-end sequencing using the HiSeq 2500 Illumina platform as described^31^. RNA was extracted for RNA sequencing using the Total RNA Isolation RNeasy Mini Kit (Qiagen, cat# 74104) according to the manufacturer’s protocol and sequenced on the same platform for paired-end sequencing.

Non-synonymous tumor mutations were identified from tumor tissues and matched blood cells. Samples from patients CRC1 and CRC2 and OvCa1-4 were analyzed as previously described^31^. Samples from patients Mel7-10 were analyzed with NeoDisc V1.2 pipeline^59^ that includes the GATK variant calling algorithm Mutect2, Mutect1, HaplotypeCaller and VarScan 2. NeoDisc v1.2 also determines the presence of each mutation and quantifies the expression of each mutant gene and mutation from RNAseq data. Predictions for binding to HLA class-I of all candidate peptides of samples from patients CRC1 and CRC2 and OvCa1-4 were performed using the NetMHC v3.4 and netMHCpan-3.0 algorithms. Predictions for binding and immunogenicity on candidate peptides of samples from patients Mel7-10 were performed using the PRIME 1.0 algorithm^29^. Candidate neoantigen-antigen peptides (i.e. mutant 9mer and 10mer peptide sequences containing the somatically altered residue) with a %rank <0.5 were synthesized.

### Antigen validation

CD8 T cells (10^6^ mL^−1^) positively isolated from PBMC (with Dynabeads CD8 positive isolation kit, Invitrogen) were co-incubated with autologous irradiated PBMCs and peptides (1 µg mL^−1^, single peptide or pools of ≤50 peptides) in RPMI supplemented with 8% human serum and IL-2 (20 IU mL^−1^ for 48 h and then 100 IU mL^−1^). IFN-γ Enzyme-Linked ImmunoSpot (ELISpot) and peptide-MHC multimer staining assays were performed at day 12. T cell reactivity for every neoantigens was validated by ≥2 independent experiments. ELISpot assays were performed using precoated 96-well ELISpot plates (Mabtech) and counted with Bioreader-6000-E (BioSys). We considered as positive conditions those with an average number of spots higher than the counts of the negative control (No Ag) plus 3 times the standard deviation of the negative. TILs were generated from tumor enzymatic digestion by plating total dissociated tumor in p24-well plates at a density of 1 × 10^6^ cells/well in RPMI supplemented with 8% human serum and IL-2 (6000 IU mL^−1^). After 2–4 weeks, TILs were collected and a fraction of the cultures underwent a rapid expansion (REP) for 14 days. T cell reactivity against predicted neoantigens was tested by IFN-γ ELISpot on pre-REP TILs, when available, and post-REP TILs as described above. Positivity was confirmed in ≥ 2 independent experiments.

### Isolation and expansion of antigen-specific CD8^+^ T cells

Circulating and tumor-infiltrating antigen-specific CD8 T cells were FACS sorted using reversible pMHC multimers (NTAmers), and were either used for TCR sequencing or cloned by limiting dilution. To this end, cells were plated in Terasaki plates and stimulated with irradiated feeder cells (PBMC from two donors) in RPMI supplemented with 8% human serum, phytohemagglutinin (1 μg mL^−1^) and IL-2 (150 IU mL^−1^). At the end of the expansion, pMHC-multimer-positive cells were ≥ 95% pure.

### Peptide synthesis

Peptides produced by the Peptides and Tetramers Core Facility (PTCF) of the University of Lausanne were HPLC purified (≥ 90% pure), verified by mass spectrometry and kept lyophilized at −80 °C.

### Production of NTAmers and peptide binding assay

NTAmers (reversible pMHC multimers) were synthesized at the Peptide and Tetramer Core Facility of the University of Lausanne as described^20^. NTAmers are composed of streptavidin-phycoerythrin (SA-PE; Invitrogen) complexed with biotinylated peptides carrying four Ni2^+^-nitrilotriacetic acid (NTA4) moieties and non-covalently bound to His-tagged pMHC monomers. For pMHC-TCR dissociation kinetics experiments, pMHC monomers were refolded with Cy5-labeled β2m. Briefly, β2m containing the S88C mutation was alkylated using Cy5-maleimide (Pierce), purified and used for further refolding assay. Peptide-MHC monomers were produced by refolding of the different HLA heavy chains in the presence of labeled β2m and peptide of interest, purified on a Superdex S75 quantified by Bradford, aliquoted and kept at −80 °C until further use. Validation and quantification of peptide binding was done by micro-scale refolding. Refolding with HLA heavy chains carrying a C-terminal BirA substrate peptide (BSP), Cy5-labeled β2m and a test peptide were performed essentially as described^20^. Human β2m was mutated S88 to C and after refolding alkylated with maleimide-PEG2-Cy5 (Pierce, Thermo Fisher Scientific) in PBS at pH 7.4. Refolding reactions were performed in 96-well plates at 4 °C for 72 h in the presence of 10 µM peptide. Incubation without peptide control. After centrifugation (3200 × *g*, 5 min), the reaction mixtures were transferred into 96-well plates and Cy5 fluorescence read on a fluorescence plate reader (Modulus, Promega). All measurements were performed in triplicates and data processed using Excel (Microsoft).

### Structural avidity assay

KIF1B_S918F_-specific T cells were obtained by co-transfecting Jurkat cells (Promega) with 500 ng each of TCRα and TCRβ chain RNA together with 300 ng each of CD8α and CD8β RNA, using a Neon electroporation system (Thermo Fisher Scientific) as previously described^44^.

Antigen-specific CD8 T cell clones (i.e. obtained from isolation and expansion of primary cells) or transfected Jurkat cells (2 × 10^5^ cells) were incubated for 40 min at 4 °C with cognate NTAmers containing streptavidin-phycoerythrin and Cy5-labeled pMHC monomers in 50 µL FACS buffer (PBS supplemented with 0.5% BSA and 2 mM EDTA), as described^15^. Irrelevant T cells were used to measure background signal and values were systematically subtracted. Specific gMFI values were plotted and analyzed using the GraphPad Prism software (v.7, or v.9, GraphPad) fitting a one phase exponential decay model.

### Functional avidity assay

Functional avidity of antigen-specific CD8 T cell responses was assessed by performing in vitro IFN-γ Enzyme-Linked ImmunoSpot (ELISpot, Mabtech) assay with limiting peptide dilutions (ranging from 10 μg mL^−1^ to 0.1 pg mL^−1^) as described^16^. EC_50_ values were derived by dose-response curve analysis (log(peptide concentration) versus response) using GraphPad Prism software (v.7 or v.9, GraphPad). The peptide concentration required to achieve a half-maximal cytokine response (EC_50_) was determined and referred to as the functional avidity.

### CD8 T cell tropism assay

PBMCs, primary CD8 T cells clones, or primary CD8 T cells transduced with engineered TCR specific for NY-ESO-1 restricted by HLA-A*0201 were distributed in 48-well plates (6 × 10^5^/well) in RPMI supplemented with 8% human serum and IL-2 (150 U mL^−1^). Cells were stimulated at 37 °C under 5% CO_2_ either with culture medium alone, phytohemagglutinin (PHA; Oxoid, 1 mg mL^−1^), OKT3 antibody (plate precoated with 30 ng mL^−1^, 5 ng mL^−1^, or 1 mg mL^−1^ in PBS), or 2 × 10^5^ T2 cells pulsed with cognate peptide (at 1 mM or 1 nM). After 48 h, cells were washed and replaced in culture for 48 h at 37 °C under 5% CO_2_ in RPMI supplemented with 8% human serum and IL-2 (150 IU mL^−1^). Half of the cells were analyzed by flow cytometry (BD LSRII flow cytometer) using the following panel of antibodies: Zombie Aqua dye (Biolegend), Pacific Blue anti-CD8 (SK1, Biolegend, dil 1/100), PE-Texas Red anti-CD3d (7D6, Invitrogen, dil 1/100), Brilliant Violet 650 anti-CX3CR1 (2A9-1, Biolegend, dil 1/50), Brilliant Violet 605 anti-CD194 (CCR4) (L291H4, Biolegend, dil 1/50), Brilliant Violet 711 anti-CD197 (CCR7) (G043H7, Biolegend, dil 1/50), FITC anti-CD49b (P1E6-C5, Biolegend, dil 1/50), PerCP/Cy5.5 anti-CD195 (CCR5) (HEK/1/85a, Biolegend, dil 1/25), Brilliant Violet 650 anti-CD196 (CCR6) (G034E3, Biolegend, dil 1/50), PE anti-CD49a (TS2/7, Biolegend, dil 1/25), PE/Cy7 anti-CD103 (Integrin αE) (Ber-ACT8, Biolegend, dil 1/10), Brilliant Violet 510 anti-CD183 (CXCR3) (G025H7, Biolegend, dil 1/25). After 5 days of resting, the remaining cells were profiled with the same panel. Data were analyzed using FlowJo 10.5.3.

### TCRα and TCRβ repertoire sequencing

mRNA was extracted using the Dynabeads mRNA DIRECT purification kit according to the manufacturer instructions (ThermoFisher, cat# 61012) and was then amplified using the MessageAmp II aRNA Amplification Kit (Ambion, cat# AM1751) with the following modifications: in vitro transcription was performed at 37 °C for 16 h. First strand cDNA was synthesized using the Superscript III (Thermofisher) and a collection of TRAV/TRBV specific primers. TCRs were then amplified by PCR (20 cycles with the Phusion from NEB) with a single primer pair binding to the constant region and the adapter linked to the TRAV/TRBV primers added during the reverse transcription. A second round of PCR (25 cycles with the Phusion from NEB) was performed to add the Illumina adapters containing the different indexes. The TCR products were purified with AMPure XP beads (Beckman Coulter), quantified and loaded on the MiniSeq instrument (Illumina) for deep sequencing of the TCRα/TCRβ chain. The TCR sequences were further processed using ad hoc Perl scripts to: (i) pool all TCR sequences coding for the same protein sequence; (ii) filter out all out-frame sequences; (iii) determine the abundance of each distinct TCR sequence. TCR with a single read were not considered for the analysis. This methodology was previously reported in Arnaud et al. and Bobisse et al.^31,44^.

### Clone TCRα and TCRβ sequencing

mRNA was extracted using the Dynabeads mRNA DIRECT purification kit according to the manufacturer instructions (ThermoFisher, cat# 61012). First strand cDNA was synthesized using oligo dT and the Superscript III (Thermofisher). Second strand was performed using a collection of TRAV/TRBV specific primer (1 cycle with the Phusion from NEB). TCRs were then amplified by PCR (20 cycles with the Phusion from NEB) with a single primer pair binding to the constant region and the adapter linked to the TRAV/TRBV primers added during the reverse transcription. A second round of PCR (25 cycles with the Phusion from NEB) was performed to add the Illumina adapters containing the different indexes. The TCR products were purified with AMPure XP beads (Beckman Coulter), quantified and loaded on the MiniSeq instrument (Illumina) for deep sequencing of the TCRα/TCRβ chain. The TCR sequences were further processed using ad hoc Perl scripts to: (i) pool all TCR sequences coding for the same protein sequence; (ii) filter out all out-frame sequences; (iii) determine the abundance of each distinct TCR sequence. TCR with a single read were not considered for the analysis. This methodology was previously reported in Schmidt et al.^29^.

### TCR transduction

TCRα/TCRβ chains were cloned in a pMSGV retroviral vector downstream of the blasticidin resistance gene followed by a P2A element. Viral particles were produced by mixing in 250 μl of Optimem medium (Life Technologies), pMSGV (1.25 μg) and packaging plasmids pMD.gagpol (1.25 μg) and pMD.G (1.25 μg, VSV-G envelope protein) with 7.5 μl of MIRUS reagent (MIRUS Bio LLC, USA). After 20 min at RT, the mix was added slowly to 10^6^ 293 T cells^60^. After 48 h, 50 μl of virus-containing supernatant were collected and added to 10^6^ primary CD8 T cells previously stimulated for 24 h with anti-CD3/anti-CD28 beads. After 24 h, medium was changed and blasticidin (Sigma-Aldrich) added at 500 μg mL^−1^. TCR expression was checked by pMHC multimer staining after 4 days. Once TCR expressing cells reached > 90% purity, they were used in functional and structural assays.

For experiments with NY-ESO-I-specific TCRs, the following procedure was followed. Full-length codon-optimized TRAV23.1 and TRBV13.1 chain sequences of a dominant HLA-A0201/NY-ESO-I_157–__165_ specific T cell clone of patient LAU155^15^ were cloned in the pRRL third generation lentiviral vectors as an hPGK-AV23.1-IRES-BV13.1 construct and structure-based amino acid substitutions were introduced into the WT TCR sequence by point mutations. Lentiviral production was performed using the calcium-phosphate method and concentrated supernatant of lentiviral-transfected 293 T cells was used to infect primary CD8 T cells overnight. Levels of TCR transduction efficacy were monitored by pMHC multimer staining^15^.

For experiments with KIF1B_S918F_-specific TCRs, the following procedure was followed. TCRα and TCRβ chains, divided by a Furin/GS linker/T2A, were cloned into a pCRRL-pGK lentiviral plasmid to produce high-titer replication-defective lentiviral particles, as previously described^61^. For primary human T cell transduction, CD8 T cells were negatively selected with beads (Miltenyi Biotec) from PBMCs of a healthy donor, activated and transduced as previously reported^61^, with minor modifications. Briefly, CD8 T cells were incubated with lentiviral particles after 24 h activation with anti-CD3/CD28 beads (Thermo Fisher Scientific) in R8 medium supplemented with 50 IU ml^−1^ IL-2. After incubation at 37 °C for 72 h, beads were removed and transduced cells were sorted with a BD FACSAria III or BD melody Cell Sorter for viability and expression of CD8 and mouse TCRβ-constant region. Sorted KIF1B_S918F_ TCR-transduced CD8 T cells were then expanded for 10 days in R8 medium and 50 IU ml^−1^ of IL-2 before mouse injection.

### Adoptive T cell transfer in immunodeficient IL-2 NOG mice and multispectral immunofluorescence staining

IL-2-NOG mice were anesthetized with isoflurane and subcutaneously injected with 10^6^ autologous human melanoma tumor cells (grown in DMEM medium supplemented with 10% FCS). Once the tumors became palpable (around day 7), 2–5 × 10^6^ human tumor-specific CD8 T cell clones^15^ were injected intravenously in the tail vein. Tumor volumes were measured by caliper twice a week and calculated as follows: volume = length × width × width/2. Mice were sacrificed by CO_2_ inhalation before the tumor volume exceeded 10^3^ mm^3^ or when necrotic skin lesions were observed at the tumor site. The same experiment with NY-ESO-I-specific TCRs was repeated but anti-CXCR3 monoclonal antibody (Biolegend) was injected i.p. (100 μg per mouse) at day 5 (simultaneously of ACT) and day 10. Additionally, for the CXCR3-blockade experiment with KIF1B-specific TCRs, anti-CXCR3 or isotype monoclonal antibodies (Biolegend) were injected i.p. (100 μg per mouse) twice a week for two weeks starting the day of ACT. Tumors were then harvested, processed and analyzed by in situ immunofluorescence labeling. Briefly, Multiplexed staining was performed on 4-micrometer formalin-fixed paraffin-embedded (FFPE) tissue sections on automated Ventana Discovery Ultra staining module (Ventana, Roche). Slides were placed on the staining module for deparaffinization, epitope retrieval (64 min at 95 °C) and endogenous peroxidase quenching (Discovery Inhibitor, 8 min, Ventana). Multiplex staining consists in multiple rounds of staining. Each round includes non-specific sites blocking (Discovery Goat IgG and Discovery Inhibitor, Ventana), primary antibody incubation, secondary HRP-labeled antibody incubation for 16 min (Discovery OmniMap anti-rabbit HRP (Ventana, # 760-4311) or anti-mouse HRP (Ventana, #760-4310)), OPAL reactive fluorophore detection (Akoya Biosciences, Marlborough, MS, USA) that covalently label the primary epitope (incubation: 12 min) and then antibodies heat denaturation. Sequence of antibodies used in the multiplex with the associated OPAL are the following: 1st, rabbit anti-CD8 antibody (4 µg/ml, Clone SP16, Cellmarque, 1 h, 37 °C), OPAL520; and 2nd, rabbit anti-SOX10 antibody (1 µg/ml, Clone EP268, CellMarque, 1 h, RT), OPAL690; Nuclei were visualized by a final incubation with Spectral DAPI (1/10, FP1490, Akoya Biosciences) for 12 min. Multiplex IF images were acquired on Vectra 3.0 automated quantitative pathology imaging system (Akoya Biosciences). Tissue and panel specific spectral library of each panel individual fluorophore and tumor tissue autofluorescence were acquired for an optimal IF signal un-mixing (individual spectral peaks) and multiplex analysis. IF-stained slides were pre-scanned at 10x magnification. Using the Phenochart whole-slide viewer (Akoya Biosciences). Whole tumor was selected and annotated for high-resolution multispectral acquisition of images at ×20 magnification. IF signal extractions were performed using inForm 2.3.0 image analysis software (Akoya Biosciences)^62^ enabling a per cell analysis of IF markers of multiplex stained tissue sections. The images were first segmented into tumor, stroma and necrosis regions, based on the cytokeratin staining using the inForm Tissue Finder algorithms. Individual cells were then segmented using the counterstained-based cell segmentation algorithm, based on DAPI staining. Quantification of the immune cells are performed using the inform active learning phenotyping algorithm by assigning the different T cell phenotypes across several images. IF-stained cohorts are then batch processed, data were exported and process via an in-house developed R-script algorithm to retrieve every cells population.

### TCR reactivity validation

To validate antigen specificity and interrogate tumor reactivity, TCRαβ pairs were cloned into recipient activated T cells. Paired α and β chains were annotated based on single-cell TCR sequencing data. For TCR cloning, DNA sequences coding the full-length TCR chains were codon optimized and synthesized by GeneArt (Thermo Fisher Scientific) or with a BioXP System (Telesis Bio). Each DNA sequence included a T7 promoter upstream of the ATG codon, whereas human constant regions of α and β chains were replaced by corresponding homologous mouse constant regions. DNA served as template for in vitro transcription (IVT) and polyadenylation of RNA molecules as per the manufacturer’s instructions (Thermo Fisher Scientific).

Autologous or HLA-matched allogeneic PBMCs were resuspended at 10^6^ cells mL^−1^ in 48-well plates in R8 medium supplemented with 50 IU mL^−1^ IL-2 (Proleukin). T cells were activated with Dynabeads Human T Activator CD3/CD28 beads (Thermo Fisher Scientific) at a ratio of 0.75 beads: 1 total PBMCs. After 3 days of incubation at 37 °C and 5% CO2, beads were removed and activated T cells cultured for two extra days before electroporation or freezing. For the transfection of TCRαβ pairs into T cells, the Neon electroporation system (Thermo Fisher Scientific) was used. Cells were resuspended at 15–20 × 10^6^ cells mL^−1^ in buffer R (buffer from the Neon kit, cat# MPK10096) and mixed with 500 µg of TCRα chain RNA together with 500 µg of TCRβ chain RNA and electroporated with the following parameters: 1600V, 10 ms, 3 pulses.

For the validation of antigen specificity, electroporated cells were interrogated by pMHC-multimer staining with the following surface panel: anti-CD3 APC Fire 750 (SK7, Biolegend, dil 1/100) anti-CD4 PE-CF594 (RPA-T4, BD Biosciences, dil 1/25), anti-CD8 FITC (SK1, Biolegend, dil 1/25) anti-mouse TCRβ-constant APC (H57-597, ThermoFisher Scientific, dil 1/25) and Aqua viability dye (ThermoFisher Scientific) (see gating strategy in Supplementary Fig. 11a). The following experimental controls were included: MOCK (transfection with water) and a control TCR (specific for a viral antigen).

Electroporated cells were incubated for 6 h and used in coculture experiments for tumor recognition and functional avidity assays. To assess antitumor reactivity of TCRs, RNA-electroporated T cells were incubated with IFNγ-treated autologous tumor cells at a ratio of 5:1 in tubes or 10^5^ electroporate T cells and 3 × 10^4^ IFNγ-treated tumor cells in IFNγ ELISpot assay. After over-night culture, cells were collected and the upregulation of 4-1BB (CD137) was evaluated by staining with anti-4-1BB PE (4B4-1, Miltenyi, dil 1/50), anti-CD3 APC Fire 50 (SK7, Biolegend, dil 1/100) or anti-CD3 APC-H7 (SK7, BD Biosciences, dil 1/100), anti-CD4 PE-CF594 (RPA-T4, BD Biosciences, dil 1/25), anti-CD8 Pacific Blue (RPA-T8, BD Biosciences, dil 1/100) and anti-mouse TCRβ-constant APC (H57-597, Thermo Fisher Scientific, dil 1/50) and with viability dye Aqua (Thermo Fisher Scientific). The following experimental controls were included: mock (transfection with water), an irrelevant TCR (random crossmatch of a TCRα and β chain). Flow cytometry was performed using LSR Fortessa (BD Biosciences) or IntelliCyt iQue Screener PLUS (Bucher Biotec) and analyzed with FlowJo v10.5.3 (TreeStar).

### Single-cell RNA and TCR sequencing

Expanded TILs from patients Mel7-10 were resuspended in PBS + 0.04% BSA and DAPI (Invitrogen) staining was performed. Live cells were sorted with a BD FACS Melody sorter and manually counted to assess viability with Trypan blue. Cells were then resuspended at 10^3^ cells µL^−1^ with a viability of > 90% and subjected to a 10X Chromium instrument for the single-cell analysis. The standard protocol of 10X Genomics was followed and the reagents for the Chromium Single Cell 5′ Library and V(D)J library (v1.0 Chemistry) were used. 12,200 cells were loaded per sample, with the targeted cell recovery of 7000 cells according to the protocol. Using a microfluidic technology, single-cell were captured and lysed, mRNA was reverse transcribed to barcoded cDNA using the provided reagents (10X Genomics). 14 PCR cycles were used to amplify cDNA and the final material was divided into two fractions: first fraction was target-enriched for TCRs and V(D)J library was obtained according to manufacturer protocol (10X Genomics). Barcoded VDJ libraries were pooled and sequenced by an Illumina HiSeq 2500 Sequencer. The second fraction was processed for 5′ gene expression library following the manufacturer’s instruction (10X Genomics). Barcoded samples were pooled and sequenced by an Illumina HiSeq 4000 sequencer.

The scRNA-seq reads were aligned to the GRCh38 reference genome and quantified using Cellranger count (10x Genomics, version 3.0.1). Filtered gene-barcode matrices that contained only barcodes with unique molecular identifier (UMI) counts that passed the threshold for cell detection were used for further analysis. The number of genes per cell averaged 1862 (median: 1729) and the number of unique transcripts per cell averaged 4886 (median: 4169). We obtained 18,378 cells (7056 for Mel7, 3656 for Mel8, 3137 for Mel9 and 4529 for Mel10). Low quality cells exhibiting more than 10% of mitochondrial reads were discarded from the analysis, resulting in a final set of 17,937 cells (6916 for Mel7, 3545 for Mel8, 3059 for Mel9 and 4417 for Mel10). The data was processed using the Seurat R package (version 3.2.2) as follows briefly: counts were log-normalized using the NormalizeData function and then scaled using the ScaleData function by regressing the mitochondrial, ribosomal contents and S phase and G2/M phase scores. Dimensionality reduction was performed using the standard Seurat workflow by principal component analysis followed by tSNE and UMAP projection (using the first 75 PCs). The k-nearest neighbors of each cell were found using the FindNeighbors function run on the first 75 PCs, and followed by clustering at several resolutions using the FindClusters function. Cells were annotated by looking at expression of the canonical PTPRC and CD3E markers where all clusters where found to be T cells. The cells were then classified as CD8-positive, CD4-positive, double-negative (DN), double-positive (DP) and Tγδ as follows: cells with non-null expression of CD8A and null expression of CD4 were defined as CD8-positive (and vice-versa for CD4-positive). Cells showing non-null expression of both genes were classified as DP. Due to notorious dropout events in single-cell data, cells lacking the expression of both markers were classified as follows: if a cell belongs to a cluster (taking a fine resolution of 10) in which the 75th percentile expression of CD8 was higher than its 75th percentile expression of CD4, it was classified as CD8-positive (and vice-versa for CD4-positive cells). If the 75th percentile expressions of both markers equal 0, the cells were classified as DN. Finally, cells with an average expression scores of all TRG and TRD-related genes higher than 0.3 were assigned to be Tγδ cells. This resulted in final set of 10,947 CD8 T cells, 5922 CD4 T cells, 852 DP, 1 DN and 132 Tγδ cells.

VDJ sequencing data were aligned to the same human genome using the Cellranger VDJ (10x Genomics, version 3.1.0). Cells from the VDJ sequencing were mapped to the scRNA-seq data and 90.7% of the T cells had a mapped TCR β-chain (84.6% for TCR α-chain).

### TCR-pMHC structure modeling and correlation with structural avidity

The Rosetta “TCRmodel” protocol^63^ was adapted to our approach and applied to find the respective templates and model TCR. The orientation of the TCR relative to the pMHC was performed based on TCR-pMHC templates retrieved from Protein Data Bank^64^ and identified using sequence similarity. Side chains and backbones of the TCR-pMHC models were refined using the fast “relax” protocol in Rosetta 3.10^65^. A total of 500 models were produced for each TCR-pMHC. These models were subsequently ranked based on a consensus approach that combines the Rosetta energy function as implemented in Rosetta 3.10^63^ and the Discrete Optimized Potential Energy as implemented in Modeller 10.1^66^. This consensus score corresponded to the sum of the normalized (Z-score) Rosetta and DOPE energies calculated over the peptide residues, as well as the CDRs and MHC residues within 6 Å from the peptide. For each TCR-pMHC, the best model according to the consensus score was selected for CDR loop refinement. The later was performed by creating 100 alternative loop conformations using the kinematic closure loop modeling of Rosetta 3.10^67^ and subsequent refinement using the fast “relax” protocol. The final TCR:pMHC structural model is the one with the highest number of favorable interactions within the top5 high-score models over the 600. Molecular graphics and analyses were performed with the UCSF 1.14 Chimera package^68^. Correlation between the mean structural avidity of each pMHC-TCR pair with the number of non-polar, *n*_apolar_, and the number of polar, *n*_polar_, contacts between modeled TCR and pMHC was obtained via the equation:

1 $${T}_{\frac{1}{2}}\left(s\right)=K+\gamma \,*\, {n}_{{{{{{\rm{apolar}}}}}}}+\delta \,*\, {n}_{{{{{{\rm{polar}}}}}}}$$

This equation represents a simplification of the binding free energy estimation^69^, where *γ* and *δ* are weighting terms applied on the number of apolar and polar contacts, respectively, and *K* is added to account for contributions that are not a function of the number of polar and non-polar contacts. The *K*, *γ* and *δ* parameters are fitted by multiple linear regression against the experimental pMHC-TCR *T*_1/2_. The parameters were optimized using 10 complexes (Supplementary Table 3) and values of −62.89 s, 2.647 s and 8.747 s were obtained for K, *γ* and *δ*, respectively. Further details regarding the modeling and correlation are available in Supplementary Methods.

### Hierarchical clustering of TCR sequences

We implemented a computational pipeline based on a biophysicochemical approach^43^ that allows TCR comparisons by analyzing the biophysicochemical properties of the 4-mer subunits that are possible to construct from CDR3β and comparing them across all the TCRs under study. To provide insights into the clusters, structural models were created for the TCRs as described in the previous section. The clustering pipeline consists of 4 main steps. First, we identified all possible sliding windows of 4 residues that constitute the so-called 4-mer subunits. The first 4 and the last 3 residues of the CDR3β are excluded from this process because these residues usually do not contact the HLA peptide. Second, each 4-mer subunit is converted into a biophysicochemical representation using 5 Atchley factors that describe (i) hydrophobicity, (ii) secondary structure, (iii) size/mass, (iv) codon degeneracy and (v) electric charge. Third, for a pair of TCRs, we compare all the *n* 4-mer subunits that are possible to construct from the first TCR with all the *m* possible 4-mer subunits of the second TCR. This results in n*m matrices to compare for each pair of TCRs. The matrices’ comparison is performed via a Manhattan distance score normalized over the maximum possible distance. This score ranges from 0, for 4-mers sharing exactly the same biophysicochemical properties, to 1, for 4-mers that have totally different biophysiochemical properties. Fourth, a distance tree is constructed using the smallest distance for each TCR pair. The generic hierarchical clustering algorithm UPGMA (unweighted pair group method with arithmetic mean) is used^70^. The clustering analysis was finally applied to a total of 58 TCRs with known pMHC, 52 of which with known avidity (Supplementary Table 4).

### Logistic regression to discriminate between low and high-avidity TCRs

We have implemented a logistic regression, based on the CDR3β amino acids that are enough solvent exposed and therefore able to interact with the peptide, to determine whether a TCR binds the cognate pMHC with high or low k_off_ value. We correlated TCR avidity with CDR3β sequence as it is generally accepted that CDR3β is the most determining CDR for antigen specificity^71,72^. The amino acids used in the logistic regression resulted from an exhaustive exploration (details below) and correspond to the optimal solution found.

The probability, *p*, of a TCR being high avidity is given by:

2 $$p=\frac{1}{1+{e}^{-({b}_{0}+{W}_{1}*R+{W}_{2}*N+{W}_{3}*D+{W}_{4}*G+{W}_{5}*I+{W}_{6}*L+{W}_{7}*F)}}$$

R, N, D, G, I, L and F take the value of 1 when the corresponding amino acid is present with a solvent accessibility higher than 30% in the CDR3β of the TCR 3D model, and 0 otherwise. The solvent accessibility of each CDR3β residue is determined as the relative solvent excluded surface area (SESA) computed with the MSMS package of the UCSF Chimera software, as described by Goddard et al.^73^. SESA is calculated by normalizing the surface area of the residue in the TCR of interest by its surface area in a reference state^74^. The bias term b0 and the weights Wn were determined using a set of 48 TCRs (TCRs with undetermined avidity and TCRs 5, 25, 26, 27 and 38 without a good 3D model were discarded and therefore without calculated solvent accessibility from Supplementary Table 4), maximizing the likelihood that each avidity prediction for these TCRs is correct. The TCRs were divided into two sets: high-avidity set with 11 TCRs (*T*_1/2_ > 60 s) and low-avidity set, with 37 TCRs (*T*_1/2_ < 60 s). Before converging to this model, we correlated TCR avidity with CDR3β sequence as it is generally accepted that CDR3β is the most determining CDR for antigen specificity^71,72^. The variables/parameters previously used resulted from an exhaustive exploration and correspond to the optimal solution found. We explored the relationship between the outcome, i.e. the avidity, and different predictors, either binomial (presence or absence of the amino acid in CDR3, presence or absence of the amino acid that is sufficiently exposed in CDR3 when within the TCR structure) or continuous (frequency of the amino acid in CDR3). Combinations of 5–8 amino acids were explored to alleviate overfitting thanks to 5–9 TCRs per explanatory variable^75^. We did 277,746 multilinear regressions (MLR) and the combinations that gave the highest correlation coefficient R2, were selected to be used in logistic regressions. The accuracy of the logistics regressions was determined by the area under the ROC curve (AUC) and by the % of correct predictions and the best solution found in the MLR was confirmed to be the optimal solution to be used in the logistic regression. The best model obtained is the one described in the upper equations and has AUC = 0.96, very close to 1, emphasizing the ability of the model to discriminate between high and low avidity. The threshold of the classifier was set to 0.5, and we predict a high-avidity structure if *P* > 0.5. Cross-validations were carried out illustrating the robustness of the approach (Supplementary Material 3). The regression trained on the full set was then applied to a library of TCRs determined by single-cell sequencing, for four melanoma patients (Mel7-10) and high and low-avidity CD8 TCRs were predicted. The library of TCRs were then tracked in bulk repertoires of blood and tumors (only β chain TCR information considered).

### Statistical analyses

Statistical analyses were performed with the GraphPad Prism software v7 and v9. Correlation analyses were performed using Pearson coefficient, nonparametric Spearman correlation, nonlinear regression, Mann–Whitney, Wilcoxon-paired and log-rank tests, that are indicated throughout.

For cumulative analyses of four melanoma patients (Mel7-10) in Fig. 4c, after pulling all TCRs for a given patient, i.e. the *n* infiltrating TCRs and m non-infiltrating TCRs, we calculate the frequency, F, of HA TCRs in this entire set of *n* + *m* TCRs. Then, for each of the n blood TCRs and m tumor TCRs, we randomly choose if it will be considered of high or low affinity, with a probability of *F*/(*n* + *m*) of being of high affinity. Subsequently, we determined the fraction of ‘random’ HA or LA TCRs in the blood and in the tumor. This process is repeated 1000 times. Finally, the *P* value is calculated as the probability to get a *%HAInf-%HANo* value in the random sets equal or higher to the value in the real set.

### Reporting summary

Further information on research design is available in the Nature Portfolio Reporting Summary linked to this article.

## Supplementary information

Supplementary Information

Reporting Summary

## Data Availability

The data generated in this study are provided within the article and in the Supplementary Information or Source Data files. All scRNA/TCR-sequencing data for patients Mel7-10 have been deposited at GEO (GSE232447). All processed scTCR-Seq used for the avidity prediction are available from the source data file. Source data are provided with this paper.

## Source data

Source Data
