# Supplementary material for: Neoantigen-specific CD8 T cells with high structural avidity preferentially reside in and eliminate tumors
Source: Nat Commun. 2023 Jun 6;14:3188. doi: 10.1038/s41467-023-38946-z (PMC10244384; doi:10.1038/s41467-023-38946-z)
Supplement: Supplementary file 1 — Supplementary Information [file 41467_2023_38946_MOESM1_ESM.pdf]

Supplementary Information for

Neoantigen-Specific CD8 T Cells with High Structural Avidity Preferentially Reside in and Eliminate Tumors

Julien Schmidt[1,2,3]†, Johanna Chiffelle[1,2]†, Marta A. S. Perez[1,4]†, Morgane Magnin[1,2]†, Sara Bobisse[1,2], Marion Arnaud[1,2], Raphael Genolet[1,2], Julien Cesbron[1,2], David Barras[1,2], Blanca Navarro Rodrigo[1,2], Fabrizio Benedetti[1,2], Alexandra Michel[1,2], Lise Queiroz[1,2], Petra Baumgaertner[1,3], Philippe Guillaume[1,2,3], Michael Hebeisen[1], Olivier Michielin[5], Tu Nguyen-Ngoc[1], Florian Huber[1,2], Melita Irving[1], Stéphanie Tissot-Renaud[1,3], Brian J. Stevenson[1,2,4], Sylvie Rusakiewicz[1,3], Denarda Dangaj Laniti[1,2], Michal Bassani-Sternberg[1,2], Nathalie Rufer[1], David Gfeller[1,4], Lana E. Kandalaft[1,2,3], Daniel E. Speiser[1], Vincent Zoete[1,4], George Coukos[1,2,5], Alexandre Harari[1,2,]*

[1]Ludwig Institute for Cancer Research, Lausanne University Hospital (CHUV) and University of Lausanne (UNIL), Agora Research Center Lausanne, Switzerland.

[2]Center for Cell Therapy, Department of Oncology, Lausanne University Hospital, Lausanne, Switzerland.

[3]Center of Experimental Therapeutics, Department of Oncology, Lausanne University Hospital (CHUV), Lausanne, Switzerland.

[4]Swiss Institute of Bioinformatics (SIB), Lausanne, Switzerland.

[5]Department of Oncology, Lausanne University Hospital, Lausanne, Switzerland.

† These authors contributed equally to this work

*Corresponding author: Alexandre Harari, AGORA, Bugnon 25A, 1011 Lausanne, Switzerland, +41795032200, Alexandre.Harari@chuv.ch

Supplementary Text

Supplementary Material 1

Structural avidity is a robust and stable physical parameter. To assess any potential bias of cell culture condition in the measurements of antigen sensitivity and/or structural avidity, we sequenced and cloned six pairs of TCRαβ chains (one high and one low functionality TCR for three distinct pMHCs: two neoantigens (SLC25A48 and PHLPP2) and one TAA (Melan-A) and transduced naïve primary CD8 T cells from healthy donors with these TCRs, as described[34]. The comparison of transduced cells relative to native T cells for each pair of TCRs shows some degree of variability in measurements of antigen sensitivity, yet the ranking of high vs low functionality cells for each antigen was maintained for all three pairs except PHLPP2 (**Supplementary Fig. 3b&d**) while the measurement of structural avidity remains more quantitatively consistent (**Supplementary Fig. 3c&e**), confirming the robustness of this physical parameter to profile T cells.

Supplementary Material 2

Features displaying consecutives G or several T are seen in the high avidity cluster region (GGGT/GGGR in TCR models #45, #48, #55, #58 or TDTQ in TCR models #3, #43, #50, #53, **Supplementary Table S4),** in agreement with the fact that G and T are more frequent in high avidity structures (17.7% and 10.4%, respectively) when compared to low avidity (17.0% and 8.9%, respectively, **Supplementary Fig. 10**). G and T are more frequent inside the cluster, with frequencies dropping by 5% and 4%, respectively, when outside the cluster **(Supplementary Table S5,** all $P<0.0001$**)**. Outside the cluster, we still observe high avidity structures with consecutive G, such as the virus-specific TCR (#4) with highest structural avidity (CDR3β is CASM**GG**AYNEQFFG) and the neoantigen-specific TCR (#17) with highest structural avidity (CDR3β is CASSITTS**GG**YEQYFG). These two TCRs do not cluster together and are not in the clustered box, because the cluster analysis is based on 4-mer features and not in 2-mer features. Three other TCRs (*i.e.* #36, #37 and #69) were of interest: with #36 of high avidity, #37 of intermediate avidity and #69 of low avidity for the same pMHC. For these three TCRs, we observed that #36 contains 4 G in the CDR3β including 2 consecutive ones; #37 contains 4 non-consecutive G and #69 only contains 3 non-consecutive G. These observations indicate that high and intermediate avidity structures do exhibit a preference for G and more specifically for consecutive G. Nevertheless, this pattern is not exclusive from high and intermediate avidity TCRs as the G does not have any side chain and therefore establishes limited interactions with the peptide, justifying the presence of some low energy structures in the clustered region. High frequency of G in high avidity structures could be explained by the fact that it allows for loop rearrangements, side chain flexibility of the residues nearby and consequently more specific and strong interactions of the nearby residues with the peptide. Amino acids N, E, I, T and Y show the largest variations in frequencies between high and low-avidity CDR3β, **Supplementary Fig. 10**, all $P<0.0001$**)**. Globally in the set of 58 complexes studied here, higher avidity between TCR and pMHC seems to be acquired by an enrichment in G that increases TCRs flexibility and adaptability[71] together with an enrichment in N, E, I, T and Y that can favor hydrophilic (H-bonds), hydrophobic as well as aromatic interactions with aromatic residues in the peptide (Π-interactions), depending on the peptide nature.

Supplementary Material 3

Residues N and I contribute with positive weights ($P<0.04$) to the avidity prediction, in agreement with their higher frequencies in high avidity structures **(Supplementary Fig. 10**

and Supplementary Table 5), while remaining amino acids in the model have negative weights as they are more frequent in low avidity structures, with the exception of G which is more frequent in the CDR3β of high avidity TCRs **(Supplementary Fig. 10 and Supplementary Table S5)**, but more accessible to the solvent in low avidity TCRs.

Supplementary Fig. 10 details amino acids frequencies in the CDR3β independently of their 3D exposure. However, in the structure-based logistic regression, we obtained significantly better models by representing the presence (1) or the absence (0) of the amino acid with a normalized solvent accessibility higher than 30% in the CDR3β of the TCR 3D model (the amino acids that have more chances to contact the peptide). Amino acid frequencies for the solvent exposed residues in CDR3β (see figure below) show some variations in amino acid composition when compared to **Supplementary Fig. 10**. Considering solvent accessibility of the residues, among others, we observe that G is now more frequent in low avidity structures. The decrease in G is expected since the expected role of these residues is to allow loop rearrangements (**Supplementary Material 2**) that help strong-interacting residues nearby (such as N, E, I, T and Y) to be displayed in front of the pMHC. As a result, G residues are hindered and exhibit decreased solvent accessibility, and consequently they are themselves exposed with lower frequency.

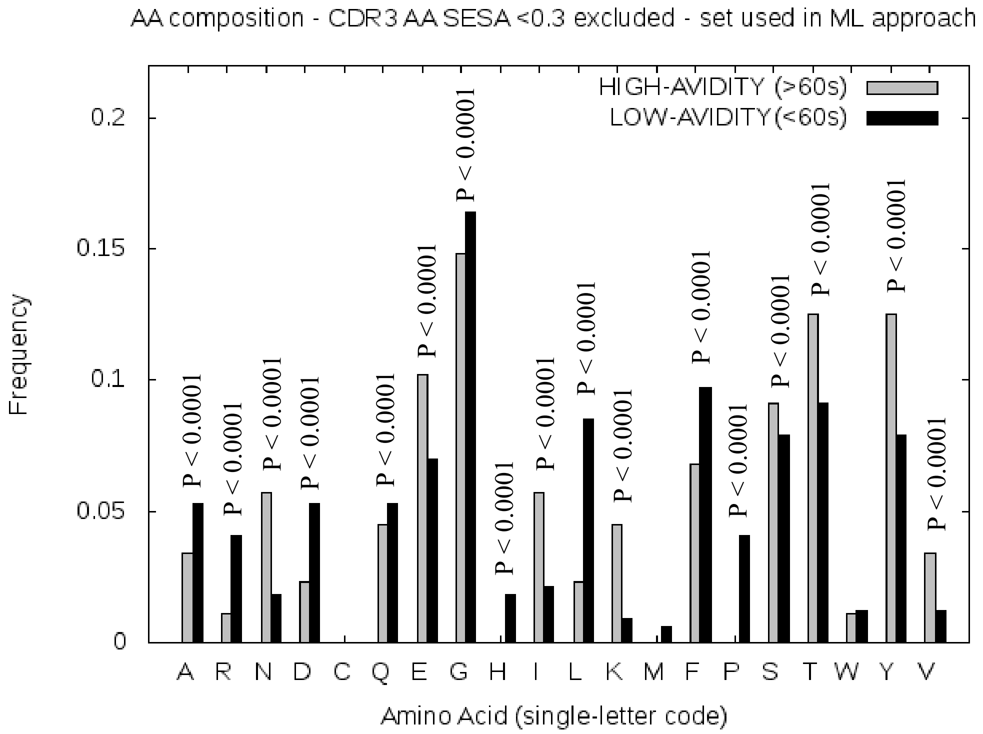

Three independent cross-validations were carried out:

First cross-validation

A first cross-validation was carried out by randomly splitting the full set into 10 sets of 38 training TCRs (3 high avidity and 7 low avidity) and 10 testing TCRs (8 high avidity and 30 low avidity). For each of the 10 combinations, we trained the model (logistic regression) from the training set and tested the predictions on the test set. Among the 10 sets, the average accuracy was 0.76±0.01. Despite the fact that the training and test sets were significantly smaller, we still observed a significant correlation, illustrating the robustness of the approach. We note that the different training and test sets include different compositions of neo-antigens, viral, TAA and different alleles, highlighting the broad applicability of the model.

Second cross-validation

For the same 10 sets we re-did the cross-validation, this time including the feature selection step in the process. The following table shows the amino acids (features) that were selected for the 10 training sets:

Set	Features	Correlation Coefficient, LR	%correct High Avidity	%correct Low Avidity
Full-set	RNDGILF	0.75	91%	97%
Training-set1	RNDGILF	0.75	88%	100%
Training-set2	RNDGILF	0.75	88%	97%
Training-set3	RNDGILF	0.72	88%	97%
Training-set4	RNDGILF	0.81	100%	97%
Training-set5	RNDGLFV	0.78	75%	97%
Training-set6	RNDILKF	0.81	100%	100%
Training-set7	DGILFPY	0.76	75%	97%
Training-set8	RNDGILF	0.75	88%	100%
Training-set9	RNDGILF	0.78	100%	100%
Training-set10	RNDILFW	0.80	100%	100%

As can be seen, RNDGIL and F are the features selected in 6/10 sets and RNDL and F were automatically selected as being the most relevant features in all the 10 sets. In addition, the sign of the coefficient of these features remained the same in all 10 cross-validations (data not shown). These results support the robustness of the model and that the CDR3β amino acid representation used is the most relevant for the data under study. Among the 10 test sets, the average accuracy was 0.76±0.01.

Third cross-validation

A leave-one-epitope-out cross validation was also done, where we trained the model on all TCRs except those recognizing one given epitope, before applying the new model on TCRs recognizing this epitope. For example, we trained the data on a set excluding the viral peptide GLCTLVAML/HLA-A*02:01 and then we applied it on a set that only includes the TCRs recognizing the GLCTLVAML/HLA-A*02:01. Then, we trained the data on the set excluding the neo antigen GRKLFGTHF/HLA-B*27:05 and we applied it on a set that only includes TCRs recognizing GRKLFGTHF/HLA-B*27:05. We did it successively for the 12 epitopes under discussion. The summary of the data is given in the table below:

		Converge	AMDLGIHKV		EAAGIGILTV		GLCTLVAML		GRKLFGTHF		IPSINVHHY		ITDQVPFSV		KQWLVWLFL		NILDAIAEI		PYMFLSEWI		QSDNGLDSDY		VFSFTATLPF		VSDGGPNLY		Full set	
Leave one epitope out		bo	constant	5.56	constant	39.97	constant	4.18	constant	4.19	constant	49.01	constant	4.07	constant	4.19	constant	76.24	constant	15.73	constant	29.78	constant	4.19	constant	3.74	constant	4.27
		b1	R	-6.17	R	-64.83	R	-5.23	R	-5.45	R	-67.74	R	-5.09	R	-5.45	R	-41.87	R	-16.36	R	-62.14	R	-4.92	R	-16.43	R	-5.52
		b2	N	4.86	N	41.35	N	4.62	N	4.94	N	68.46	N	4.79	N	4.94	N	25.86	N	17.11	N	51.63	N	4.82	N	4.80	N	5.00
		b3	D	-4.31	G	-40.66	D	-4.20	D	-4.25	D	-67.60	D	-4.19	D	-4.25	D	-41.93	D	-15.84	D	-30.30	D	-4.30	D	-3.98	D	-4.35
		b4	G	-4.66	I	42.45	G	-3.48	G	-3.57	G	-32.31	G	-3.54	G	-3.57	G	-41.64	G	-15.34	G	-30.17	G	-3.45	G	-3.14	G	-3.67
		b5	I	3.49	M	-101.85	I	3.27	I	3.51	I	36.83	I	3.46	I	3.51	L	-3.52	I	2.29	I	52.56	I	3.23	I	3.20	I	3.54
		b6	L	-4.14	P	-30.23	L	-3.85	L	-3.82	L	-70.13	L	-3.59	L	-3.82	F	-33.45	L	-2.81	L	-1.96	L	-3.88	L	-3.65	L	-3.85
		b7	F	-3.29	T	-40.66	F	-3.04	F	-3.08	F	-34.57	F	-2.96	F	-3.08	S	-32.56	F	-15.84	F	-50.71	F	-2.84	F	-2.85	F	-3.09
			HA	LA	HA	LA	HA	LA	HA	LA	HA	LA	HA	LA	HA	LA	HA	LA	HA	LA	HA	LA	HA	LA	HA	LA	HA	LA
Training	epitope out	Accuracy	0.89	0.94	0.80	0.96	0.9	0.97	0.91	0.97	1.00	1.00	0.90	0.97	0.91	0.97	0.91	0.97	0.89	1.00	0.90	1.00	0.91	0.97	0.90	0.97	0.91	0.97
			0.92		0.88		0.94		0.94		1.00		0.93		0.94		0.94		0.94		0.95		0.94		0.94		0.96	
Validation	epitope	Accuracy	1.00	1.00	None	0.67	1.00	1.00	None	1.00	0.33	None	1.00	1.00	None	1.00	None	1.00	0.00	0.50	0.00	0.50	None	0.67	0.00	1.00		
			1.00		0.67		1.00		1.00		0.33		1.00		1.00		1.00		0.25		0.25		0.67		0.50			

Ten leave-one-epitope-out sets, among the 12, converged to the same features (with the same negative and positive signs) as in the full set: R, N, D, G, I, L and F. The two sets that converge to different variables are the ones leaving the EAAGIGILTV (the pink epitope in **Fig. 4b**) and the NILDAIAEI epitopes out. However, they still keep 4/7 and 6/7 common features, respectively, with the full set. N and I, highlighted in yellow, are the unique amino acids contributing positively to the prediction in all the 12 sets.
When EAAGIGILTV (the most frequent epitope, represented in pink in **Fig. 4b**) is removed from the training set, we create the smallest and most different training set. Here, we removed 32% of the TCRs used in the full set, all of them with low avidity, making the situation the most different compared to the full set, and therefore the most challenging. Despite this, we still keep the features R, N, G and I in the predictor, with the same positive and negative signs. The overall accuracy leaving this epitope out is 88%, the smallest among all the sets but still satisfactory. The accuracy of the avidity predictions for TCRs recognizing this epitope, even if we did not train on this epitope and they are all of low-avidity, is 67%. The present cross-validation shows that we have predictive power for new epitopes, even in extreme cases, as the one excluding EAAGIGILTV.
For these epitope-out cross validations, the overall average prediction is 72% for the validation sets (predictions for the validation epitope, the epitope not used in the training). The predictions range from 25% to 100%. Of note, the number of TCRs per epitope range from 1 to 12, with an average of 4 and a median of 3. So, for some cases, one single wrong prediction in the validation sets, drastically decrease the accuracy. All these three cross-validation schemes support the reliability of the predictor for the data from this study. The predictor is relevant for TAA and neoantigen and viral peptides and different HLA alleles but can be the subject of further improvements when more data will be available.

Supplementary Methods

TCR-pMHC structure modeling and correlation with structural avidity

Starting from V and J segment identifiers and from the CDR3 sequences, the full sequence of the constant and variable domains of TCRα and TCRβ were reconstituted based on IMGT/GENE-DB reference sequences[1]. Homology models of the TCR-pMHC complexes were generated using the Rosetta 3.10 program[2]. Template libraries include TCR, TCR-pMHC and pMHC structures retrieved from Protein Data Bank[3]. The Rosetta "TCRmodel" protocol[4] was adapted to our approach and applied to find the respective templates and model TCR. Input α and β chains TCR amino acid sequences were parsed using regular expressions to identify CDR1, CDR2 and CDR3 loop regions and framework regions within the variable regions. Top matching CDR and framework templates were identified from the template library using the BLOSUM62 scoring function[5]. Grafting of CDR loop stem residues onto framework region was performed entailing superposition of residue backbone atoms for CDR N and C terminus onto corresponding framework residues. When one structural template matched exactly the CDR1 and CDR2 loops (germline gene template match) only the CDR3 loop was grafted. The orientation of the modeled Vα and Vβ structure was performed based on Vα/Vβ templates, while the orientation of the TCR relative to the pMHC was performed based on TCR-pMHC templates retrieved from Protein Data Bank[3] and identified using sequence similarity. Side chains and backbones of the TCR-p-MHC models were refined using the fast "relax" protocol in Rosetta[6]. A total of 500 models were produced for each TCR-pMHC. These models were subsequently ranked based on a consensus approach that combines the Rosetta energy function as implemented in Rosetta[4] and the Discrete Optimized Potential Energy as implemented in Modeller[7]. This consensus score corresponded to the sum of the normalized (Z-score) Rosetta and DOPE energies calculated over the peptide residues, as well as the CDRs and MHC

residues within 6 Å from the peptide. For each TCR-pMHC, the best model according to the consensus score was selected for CDR loop refinement. The later was performed by creating 100 alternative loop conformations using the kinematic closure loop modeling of Rosetta[8] and subsequent refinement using the fast “relax” protocol. Molecular interactions were analyzed in the top5 ranked models over the 600. The final TCR-pMHC structural model is the one with the highest number of favorable interactions within the top 5 high-score models. In these structures, TCRα is chain A, TCRβ is chain B, peptide is chain C, MHC is chain D. Residue numbers start from 1 for each chain. Molecular graphics and analyses are presented for this structure making use of the UCSF Chimera package[9]. We have monitored the interactions between the residues based on the VDW radii of their atoms. The contacts between two atoms i and j are defined as the sum of their VDW radii minus the distance between them and minus an allowance for potentially hydrogen-bonded pairs $overlap_{ij} = r_{VDWi} + r_{VDWj} - d_{ij}$. We use a negative cutoff value of 0.4 Å.

Correlation between the mean structural avidity of each pMHC-TCR pair with the number of non-polar, n_{apolar}, and the number of polar, n_{polar}, contacts between modeled TCR and pMHC was obtained via the equation:

$$T_{\frac{1}{2}}(s) = K + \gamma * n_{apolar} + \delta * n_{polar} \quad (1)$$

This equation represents a simplification of the binding free energy estimation[10], where γ and δ are weighting terms applied on the number of apolar and polar contacts, respectively, and K is added to account for contributions that are not a function of the number of polar and non-polar contacts. The K, γ and δ parameters are fitted by multiple linear regression against the experimental pMHC-TCR $T_{1/2}$. The parameters were optimized using the 10 complexes (**Supplementary Table 3**) and values of -62.89 s, 2.647 s and 8.747 s were obtained for K, γ and δ, respectively. The correlation coefficient R, the leave-one-out correlation coefficient, the standard deviation and the p-value are 0.8679, 0.6928, 24 s and 0.005, respectively. The relevance of this correlation was assessed by a randomization test. The latter consisted in attributing randomly, for each TCR, the $T_{1/2}$ of another TCR (paying attention that each of the ten $T_{1/2}$ obtained experimentally was re-attributed only once), before applying the same multiple linear regression. This randomization test was performed 10’000 times. A better correlation with the randomized $T_{1/2}$ than with the true $T_{1/2}$ was obtained for only 0.5% of the tests, in agreement with a p-value of 0.005.

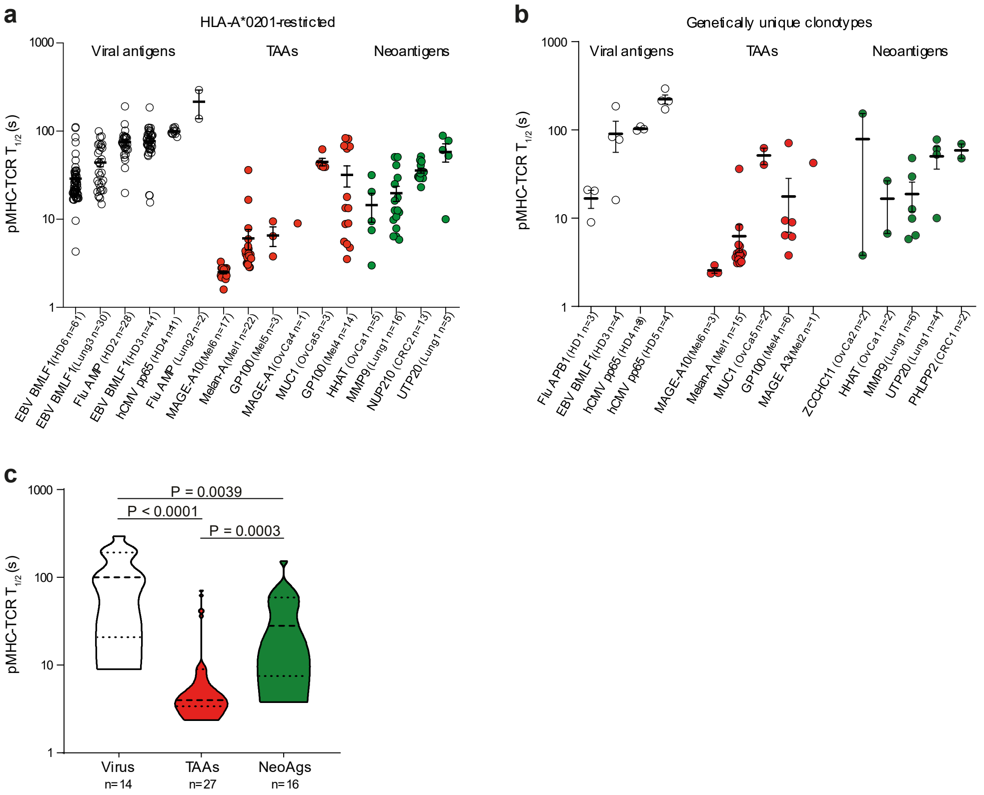

Supplementary Fig. 1. Structural avidity of neoantigen-, TAA- and virus-specific CD8 T cells. a. Structural avidity (mean±SEM) of HLA-A*0201-restricted virus-, TAA- or neoantigen-specific CD8 T cells measured by reversible pMHC multimers (NTAmers). The number of clones is indicated in brackets (n=1 independent experiment per clone). **b**. Structural avidity of genetically unique TCR-restricted virus-, TAA- or neoantigen-specific CD8 T cells measured by NTAmers (mean±SEM). The number of clones is indicated in brackets (n=1 independent experiment per clone). **c**. Cumulative structural avidities per classes of antigen (virus, TAAs and neoantigen)-specific CD8 T cells restricted to unique TCR clonotypes described in **Supplementary Table 1**. The number of clones is indicated in brackets. Exact P-values are provided at 95% confidence interval and using two-sided Mann-Whitney test. Source data are provided as a Source Data file.

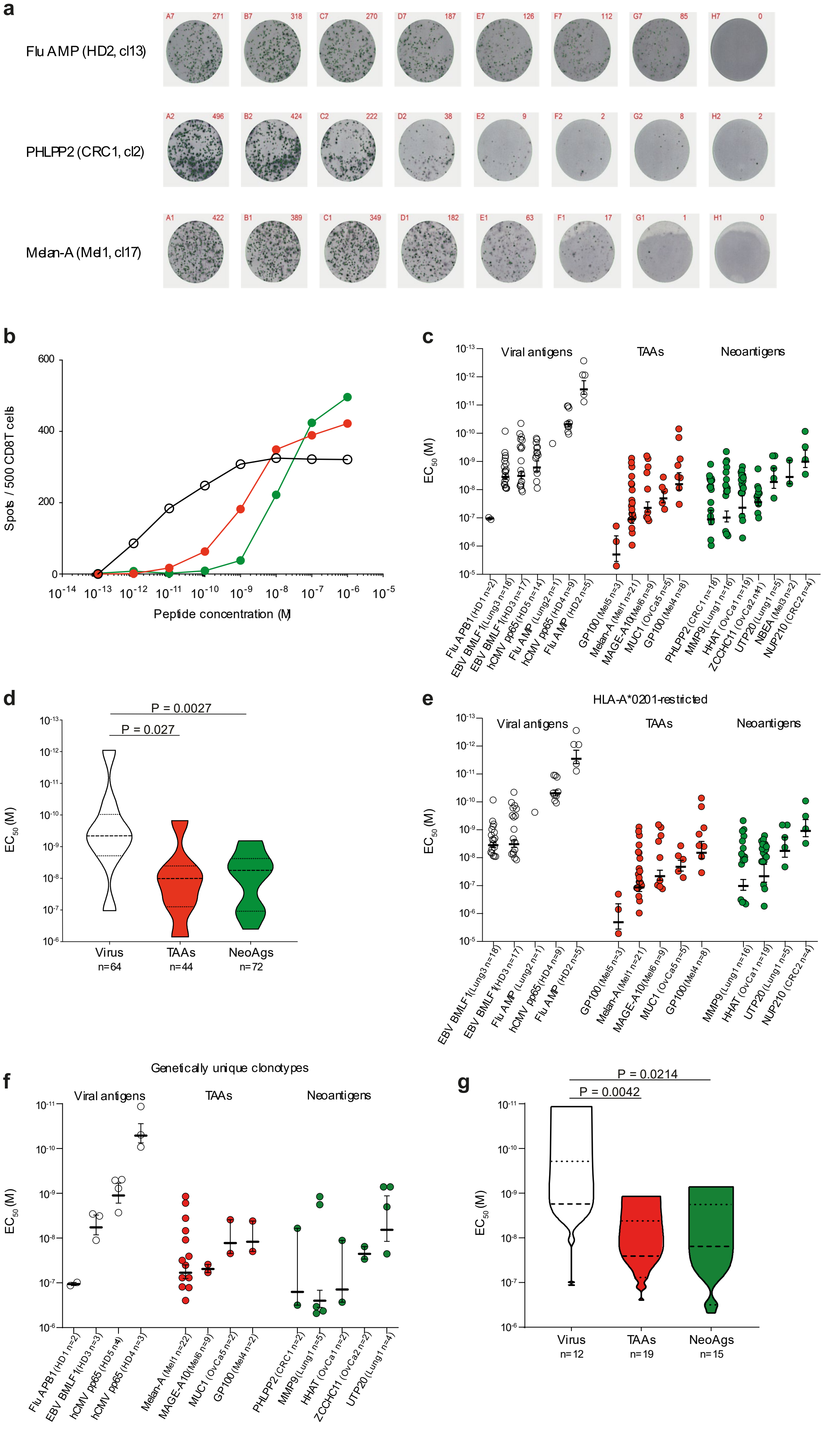

a
Flu AMP (HD2, cl13)
PHLPP2 (CRC1, cl2)
Melan-A (Mel1, cl17)
b
Spots / 500 CD8T cells
Peptide concentration (M)
c
Viral antigens
TAAs
Neoantigens
EC50 (M)
Flu APB1 (HD1 n=2)
EBV BMLF1 (Lung3 n=18)
EBV BMLF1 (HD3 n=17)
hCMV pp65 (HD5 n=14)
Flu AMP (Lung2 n=1)
hCMV pp65 (HD4 n=9)
Flu AMP (HD2 n=5)
GP100 (Mel5 n=3)
Melan-A (Mel1 n=21)
MAGE-A10 (Mel6 n=9)
MUC1 (OvCa5 n=5)
GP100 (Mel4 n=8)
PHLPP2 (CRC1 n=18)
MMP9 (Lung1 n=16)
HHAT (OvCa1 n=19)
ZCCHC11 (OvCa2 n=11)
UTP20 (Lung1 n=5)
NBEA (Mel3 n=2)
NUP210 (CRC2 n=4)
d
P = 0.0027
P = 0.027
EC50 (M)
Virus n=64
TAAs n=44
NeoAgs n=72
e
HLA-A*0201-restricted
Viral antigens
TAAs
Neoantigens
EC50 (M)
EBV BMLF1 (Lung3 n=18)
EBV BMLF1 (HD3 n=17)
Flu AMP (Lung2 n=1)
hCMV pp65 (HD4 n=9)
Flu AMP (HD2 n=5)
GP100 (Mel5 n=3)
Melan-A (Mel1 n=21)
MAGE-A10 (Mel6 n=9)
MUC1 (OvCa5 n=5)
GP100 (Mel4 n=8)
MMP9 (Lung1 n=16)
HHAT (OvCa1 n=19)
UTP20 (Lung1 n=5)
NUP210 (CRC2 n=4)
f
Genetically unique clonotypes
Viral antigens
TAAs
Neoantigens
EC50 (M)
Flu APB1 (HD1 n=2)
EBV BMLF1 (HD3 n=3)
hCMV pp65 (HD5 n=4)
hCMV pp65 (HD4 n=3)
Melan-A (Mel1 n=22)
MAGE-A10 (Mel6 n=9)
MUC1 (OvCa5 n=2)
GP100 (Mel4 n=2)
PHLPP2 (CRC1 n=2)
MMP9 (Lung1 n=5)
HHAT (OvCa1 n=2)
ZCCHC11 (OvCa2 n=2)
UTP20 (Lung1 n=4)
g
P = 0.0214
P = 0.0042
EC50 (M)
Virus n=12
TAAs n=19
NeoAgs n=15

Supplementary Fig. 2. Functional avidity of neoantigen-, TAA- and virus-specific CD8 T cells. a. Representative IFN-γ ELISPOT raw data for each class of antigen. Peptide concentration ranging from 10^{-6} M (left) to 10^{-13} M (right). **b**. Representative IFN-γ ELISPOT curves for each class of antigen (viral in black, TAA in red and neoantigen in green). **c**. Antigen sensitivity of individual virus-, TAA- and neoantigen-specific CD8 T cells measured by IFN-γ ELISpot assay (mean±SEM). The number of clones is indicated in brackets (n=1 independent experiment per clone). **d**. Median antigen sensitivity per classes of antigen (virus, TAA and neoantigen)-specific CD8 T cells. The number of clones is indicated in brackets. Mann-Whitney two-sided tests were used to calculate P-values. **e**. Antigen sensitivity of HLA-A*0201-restricted virus-, TAA- and neoantigen-specific CD8 T cells (mean±SEM). The number of clones is indicated in brackets (n=1 independent experiment per clone). **f**. Antigen sensitivity of genetically-unique TCR-restricted virus-, TAA- and neoantigen-specific CD8 T cells measured by IFN-γ ELISpot assay (mean±SEM). The number of clones is indicated in brackets (n=1 independent experiment per clone). **g**. Cumulative antigen sensitivity per classes of antigen (virus, TAA and neoantigen)-specific CD8 T cells restricted to unique TCR clonotypes described in **Supplementary Table 1**. The number of clones is indicated in brackets. P-values are provided at 95% confidence interval and using two-sided Mann-Whitney test. Source data are provided as a Source Data file.

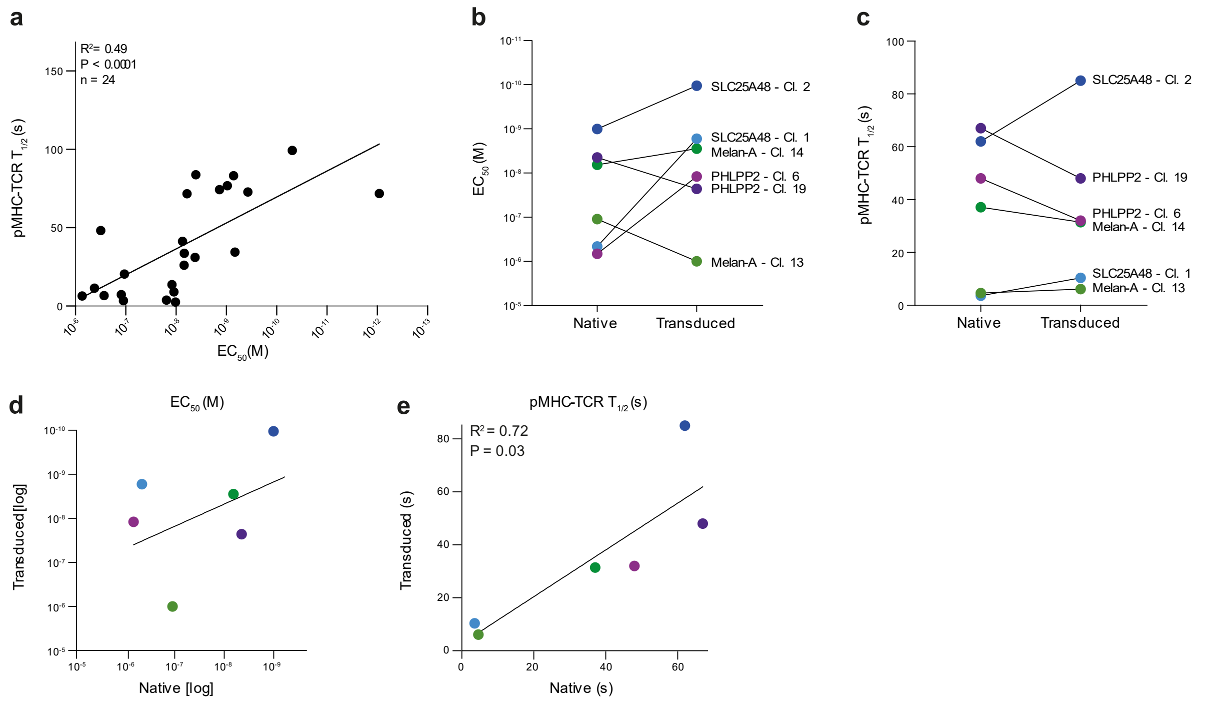

Supplementary Fig. 3. Correlation between antigen sensitivity and structural avidity measurement and cross-validation in native clones or TCR-transduced cells. a. Correlation between antigen sensitivity and structural avidity of antigen-specific CD8 T cells. Medians of antigen specificity and structural avidities were calculated for each specificity, separating clones originating from TILs or PBLs. Spearman's coefficient R^2 and exact P-values are indicated when significant and provided at 95% confidence interval and using two-sided t-test. **b-c**. Paired analyses of (**b**) the antigen sensitivity (EC_{50} (M) measured by IFN-γ ELISpot assay) or (**c**) the structural avidity (monomeric pMHC-TCR dissociation kinetics, $T_{1/2}$ (s)) of individual native TAA- and neoantigen-specific CD8 T cells and cells transduced with respective cognate TCRs (n=2 independent experiments). **d-e**. Associations between the antigen sensitivity (**d**) and the structural avidity (**e**) of native clones or TCR-transduced cells. TCRs are described in **Supplementary Table 1.** Spearman's coefficient R^2 and P-values are indicated when significant and provided at 95% confidence interval and using two-sided t-test. Source data are provided as a Source Data file.

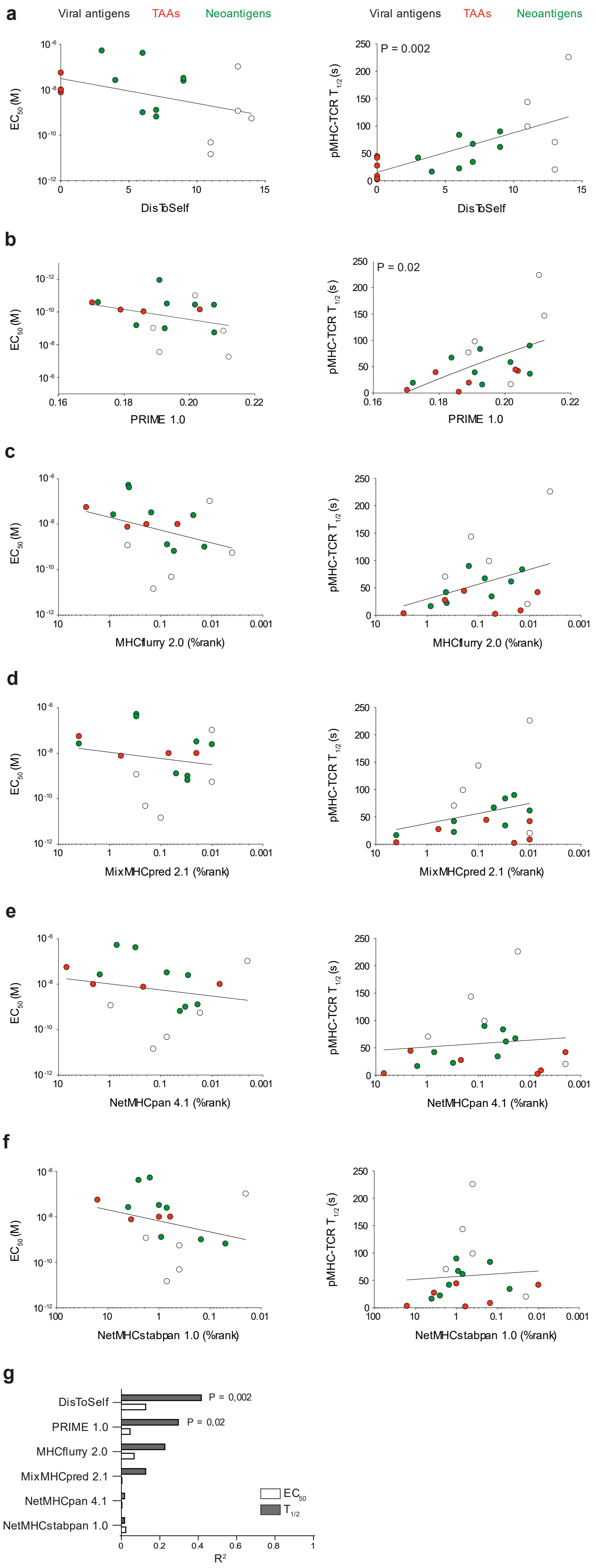

a
Viral antigens
TAAs
Neoantigens
EC50 (M)
DisToSelf
pMHC-TCR T1/2 (s)
P = 0.002
b
PRIME 1.0
P = 0.02
c
MHCflurry 2.0 (%rank)
d
MixMHCpred 2.1 (%rank)
e
NetMHCpan 4.1 (%rank)
f
NetMHCstabpan 1.0 (%rank)
g
DisToSelf
PRIME 1.0
MHCflurry 2.0
MixMHCpred 2.1
NetMHCpan 4.1
NetMHCstabpan 1.0
P = 0,002
P = 0,02
EC50
T1/2
R2

Supplementary Fig. 4. Correlations between antigen sensitivity and structural avidity of neoantigen-, TAA- and virus-specific CD8 T cells and their predicted peptide-MHC binding parameters, dissimilarity-to-self or immunogenicity. **a-f**. Median values of antigen sensitivity (left) or structural avidity (right) obtained respectively by IFNγ ELISpot assay and reversible pMHC multimers on virus- (white), TAA- (red) and neoantigen-specific (green) CD8 T cells clones and their association with (**a**) Dissimilarity-to-self (DisToSelf)[11], (**b**) PRIME 1.0[12], (**c**) MHCflurry 2.0[13], (**d**) MixMHCpred 2.1[14], (**e**) NetMHCpan 4.1[15] and (**f**) NetMHCstabpan 1.0[16]. Spearman's coefficient R^2 and P-values are indicated when significant and provided at 95% confidence interval and using two-sided t-test. **g**. Coefficient of determination R^2 of the regression analyses between different predictors values and the medians of $T_{1/2}$ (s) or EC_{50} (M) of unique CD8 T cell clones. P-values are provided when significant at 95% confidence interval and using two-sided t-tests. Unique clones are described in **Supplementary Table 1**. Source data are provided as a Source Data file.

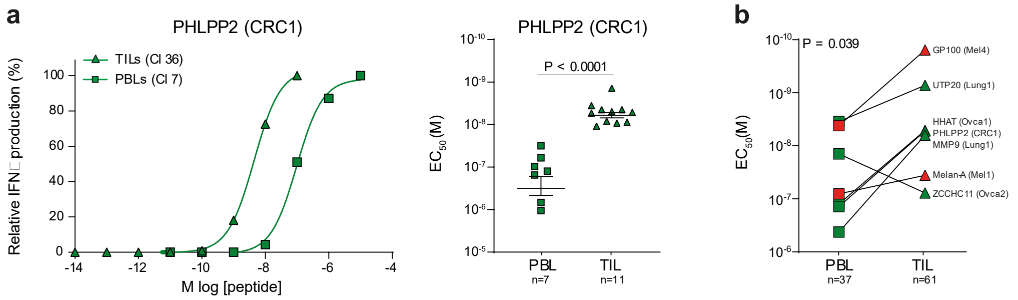

Supplementary Fig. 5. Antigen sensitivity of neoantigen- and TAA-specific CD8 PBLs and TILs. **a**. Representative examples (left) and cumulative analyses (mean±SD) (right) of the antigen sensitivity of PHLPP2-specific PBLs (squares) and TILs (triangles) from patient CRC1 assessed by IFNγ ELISpot assay. The number of clones is indicated in brackets (n=1 independent experiment). Exact P-values are provided at 95% confidence interval and using two-sided Mann-Whitney test. **b**. Comparison of the antigen specificity of seven pairs of PBL and TIL each recognizing the same pMHCs. The number of clones is indicated in brackets (n=1 independent experiment). P-values are provided at 95% confidence interval and using two-sided Wilcoxon test. Source data are provided as a Source Data file.

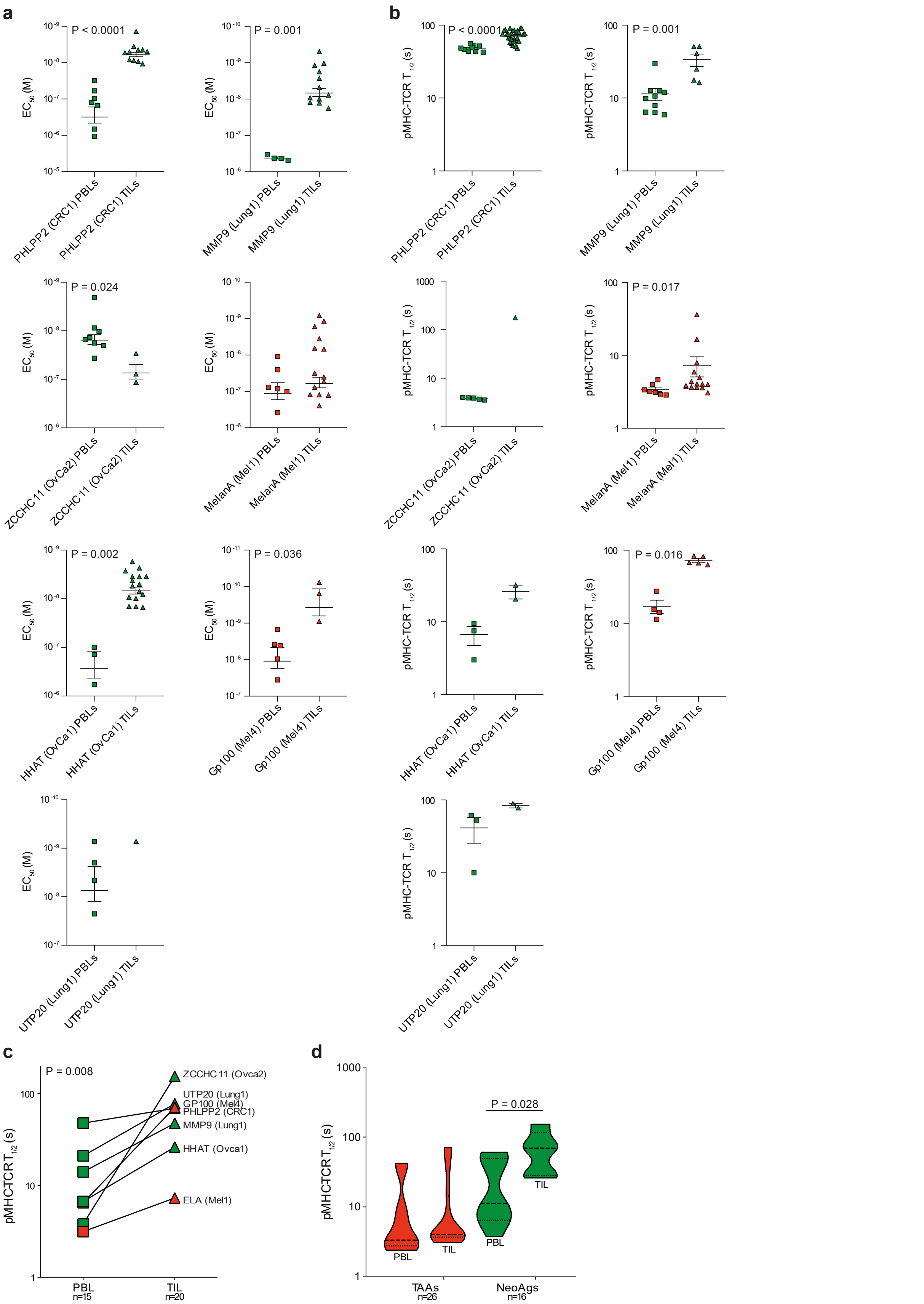

Supplementary Fig. 6. Functional and structural avidity of TAA- and neoantigen-specific CD8 T cells isolated from PBLs and TILs. a-b. Antigen sensitivity (**a**) and structural avidity (**b**) of TAA- (red) or neoantigen- (green) specific CD8 T cells originating from PBLs (square) and TILs (triangles)

assessed by IFNγ ELISpot assay and reversible pMHC multimers, respectively (n=1 independent experiment per clone, data are presented as Mean±SEM). Exact P-values are provided at 95% confidence interval and using two-sided Mann-Whitney test and are indicated when significant. **c**. Comparison of the structural avidity of seven pairs of PBLs and TILs recognizing the same pMHCs. The number of genetically unique clones is indicated in brackets. P-values are provided at 95% confidence interval and using two-sided Wilcoxon test. **d**. Structural avidity of TAA- and neoantigen-specific PBLs and TILs restricted to genetically unique clones. The number of unique clones is indicated in brackets. P-values are provided at 95% confidence interval and using two-sided Mann-Whitney test. Source data are provided as a Source Data file.

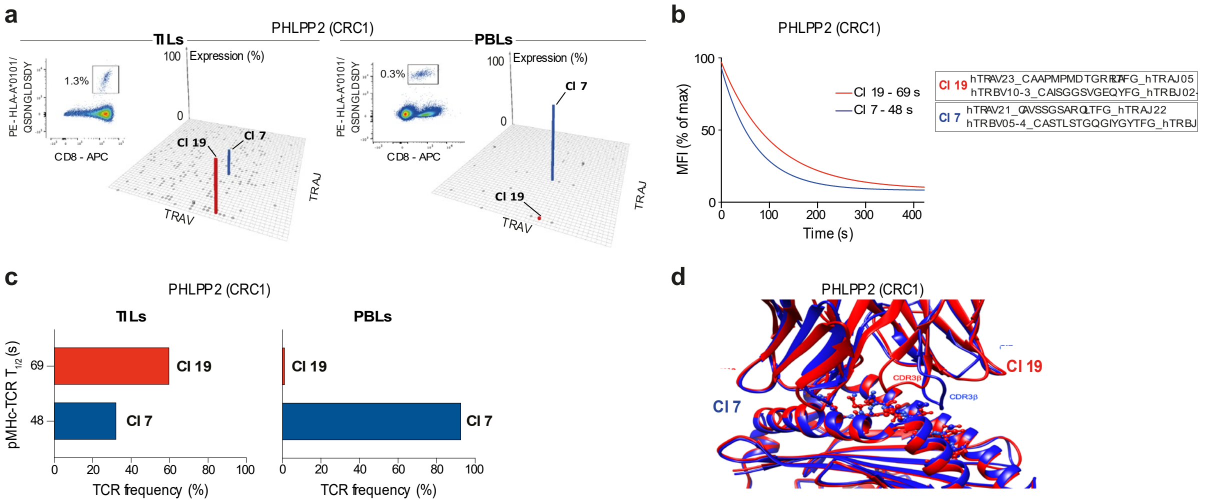

Supplementary Fig. 7. Structural avidity of PBLs and TILs clonotypes targeting the same neoepitope. a. PHLPP2-specific CD8 T cells from patient CRC1 were sorted from TILs and PBLs using NTAmers, bulk TCR sequenced and cloned by limiting dilution. The Manhattan plots of TCRα repertoires are shown and only clonotypes identified in both PBLs and TILs repertoires are color-coded. **b**. Monomeric pMHC-TCR dissociation kinetics of two PHLPP2-specific clones of patient CRC1 assessed with NTAmers. **c**. Relative frequency of clones 7 and 19 among PHLPP2-specific CD8 TILs (left) and PBLs (right). Structural avidity for each clone is also plotted. **d**. Superimposition of *in silico* analyses of the pMHC-TCRs molecular interactions for PHLPP2-specific clones 7 and 19. Blue and red are used to color TCR ribbons, MHC and peptides (ball and stick) for clones 7 and 19, respectively. Source data are provided as a Source Data file.

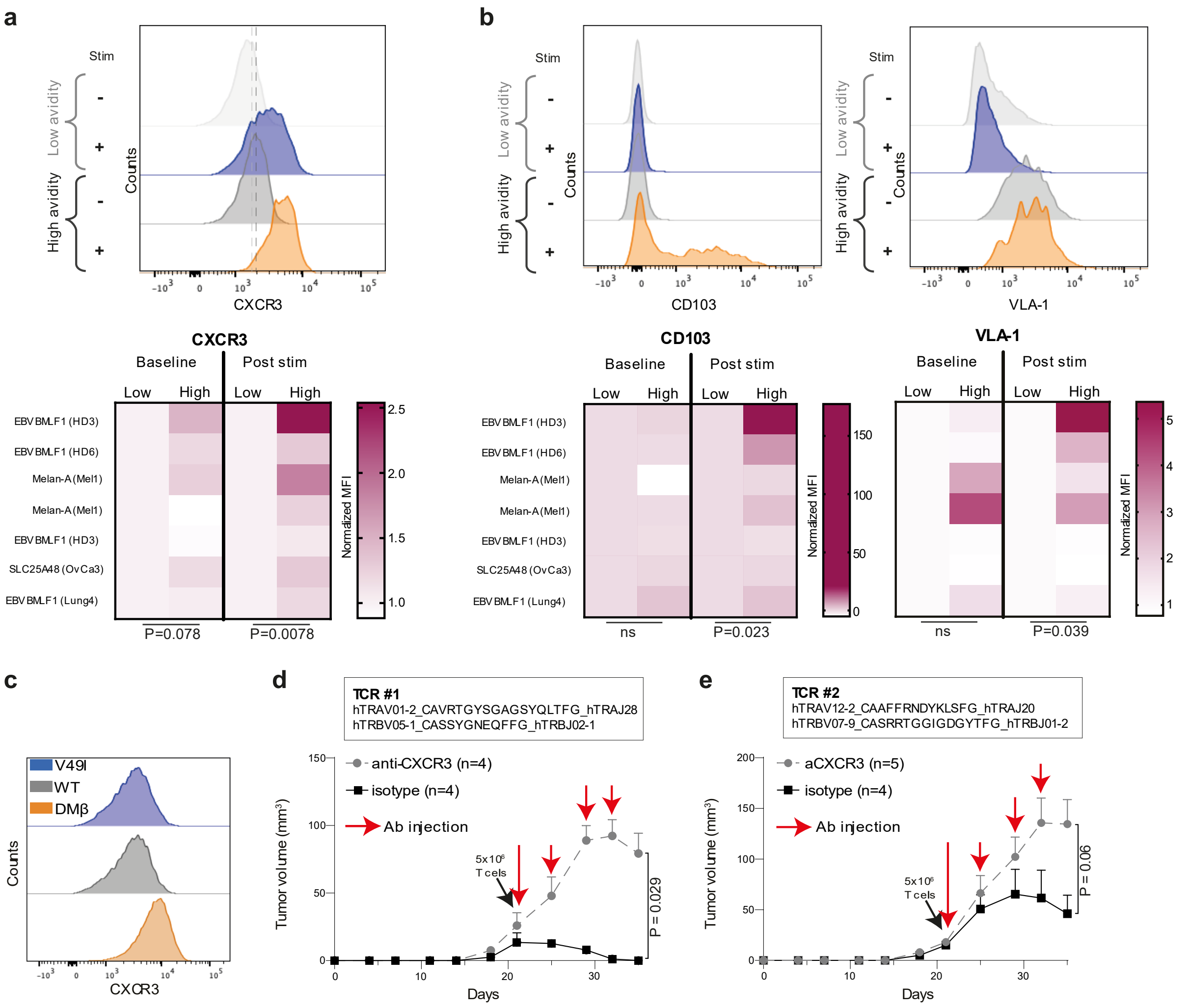

Supplementary Fig. 8. CXCR3, CD103 and VLA-1 (CD49a) expression by high avidity clones and CXCR3-mediated tumor control. a. CXCR3 expression (top) on BMFL1-specific CD8 T cell clones 4 (low avidity) and 2 (high avidity) from patient Lung4. Grey histograms represent basal expression while colored histograms show CXCR3 expression after 48h stimulation with T2 cells pulsed with 1µM of peptide GLCTLVAML. Dotted grey bars show geometric mean fluorescence intensity of unstimulated low and high avidity clones respectively. Heat maps (bottom panel) of the basal and post-stimulation expression of CXCR3 in seven pairs of high and low structural avidity virus-, TAA- and neoantigen-specific CD8 T cells. P-values are provided at 95% confidence interval and using two-sided Wilcoxon test. **b**. Same as a. but representing expression of CD103 (clones EBV BMLF1 from HD3) and VLA-1 (clones EBV BMLF1 from Lung4). P-values are provided at 95% confidence interval and using two-sided Wilcoxon test. **c**. CXCR3 expression by CD8 T cells transduced with V49I, WT or DMβ NY-ESO-$I_{157–165}$-specific TCRs after 48h stimulation with peptide-pulsed T2 cells. **d-e**. Inhibition of tumor (Mel8) control with anti-CXCR3 blocking antibody (circle) or an isotype control (square) in IL-2 NOG mice (n=4 mice for all except TCR #2 + aCXCR3 where n=5 mice) adoptively-transferred with $5x10^6$ autologous primary T cells transduced with neoantigen-specific TCRs. CXCR3 blocking prevented the complete tumor control achieved with the high avidity $KIF1B_{S918F}$-specific TCR#1 (**d**) while it led to a 2-3 fold higher tumor volume in the model with the low avidity $KIF1B_{S918F}$-specific TCR#2 (**e**). In both models, 100µg of anti-CXCR3 blocking antibody or an isotype control were injected at day 21, 25, 29 and 32. Data are presented as Mean ±SEM. Log rank two-sided tests were used to determine P-values. Source data are provided as a Source Data file.

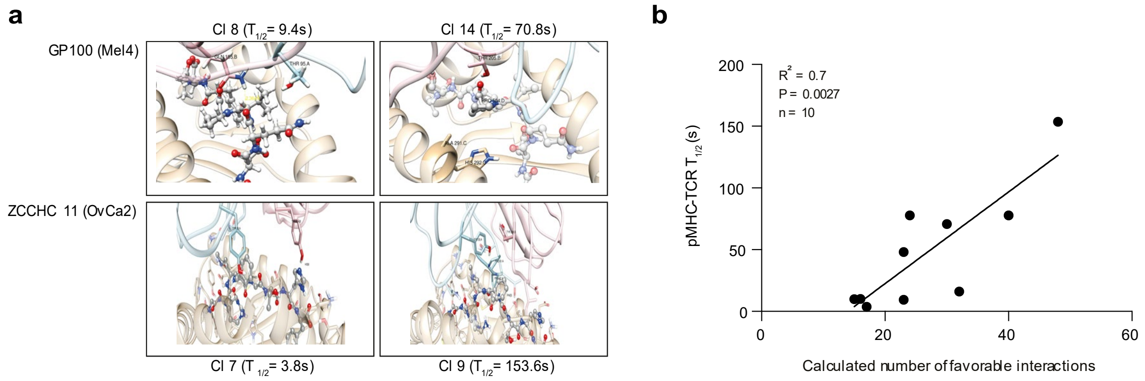

Supplementary Fig. 9. Molecular modeling of predicted high and low structural avidity clones. a. Molecular modeling of pMHC-TCR interactions. GP100 clones 8 and 14 from patient Mel4 are displayed on top. ZCCHC11 clones 7 and 9 from patient OvCa2 are shown below. TCRα ribbon is colored in light blue, with residues displayed in sticks and colored according to the atom types with carbon colored in light blue. TCRβ is colored in pink, with residues displayed in sticks and colored according to the atom types, with carbon colored in pink. MHC is colored in brown, with residues displayed in sticks and colored according to the atom types, with carbon colored in brown. The peptide is shown in grey ball and stick and colored according to the atom types and carbon colored in grey. Residues are labelled in black. **b**. Correlation between the number of calculated favorable interactions between pMHC-TCR and the structural avidity of virus-, TAA- or neoantigen-specific primary CD8 T cells. Spearman's coefficient R^2 and P-values are provided at 95% confidence interval and using two-sided t-test. Source data are provided as a Source Data file.

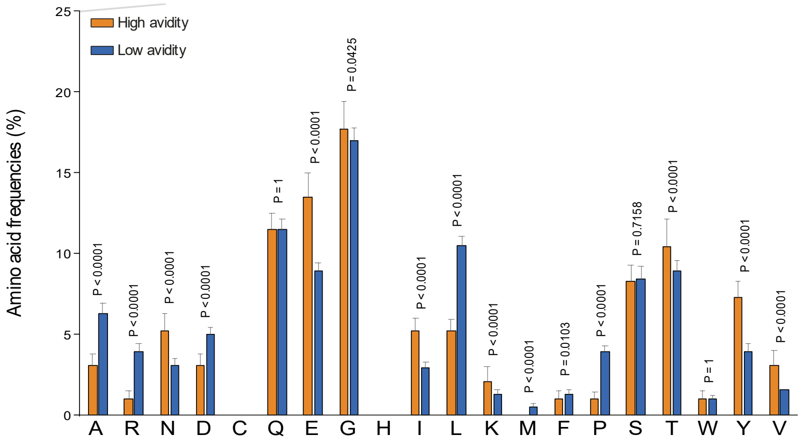

Supplementary Fig. 10. Amino acids frequencies in high and low structural avidity TCRs. Amino acid frequencies of the CDR3β, excluding the first four and last three residues that may not contact the peptide, in agreement with the biophysical-based approach from Atchley[17] for TCRs with high structural avidity (orange, n=12) or low structural avidity (blue, n=39). High avidity is considered as $T_{1/2} > 60s$. Data are presented as Mean ±SD. A significance value of the differences between high and low- avidity (exact p-value) is provided at 95% confidence interval and using two-sided t-test. Source data are provided as a Source Data file.

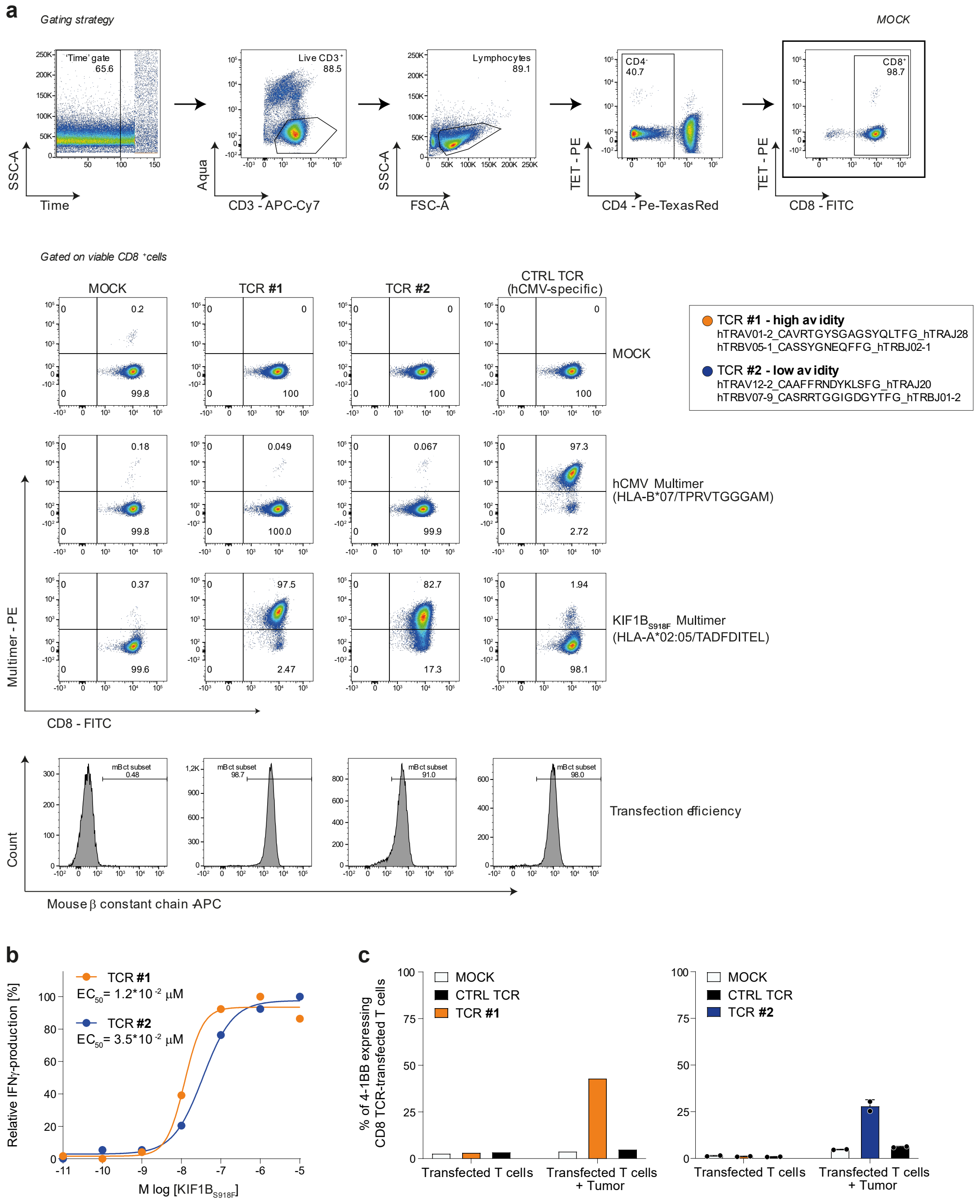

Supplementary Fig. 11. Validation of antigen- and tumor-specificity of neoepitope-specific TCR-transfected cells. a. Validation of antigen-specificity of the two clones selected for the adoptive cell transfer in **Fig. 4d-e**. The RNA coding for two KIF1B$_{S918F}$-specific TCRs was transfected into recipient activated T cells followed by staining with the KIF1B$_{S918F}$-specific multimer. A virus-specific TCR targeting the CMV pp65 peptide TPRVTGGGAM (HLA-B*07) was used as irrelevant control. **b**. The functional avidity of both KIF1B$_{S918F}$-specific TCRs was measured using TCR-transfected activated T cells. Shown are the normalized relative frequencies of IFNγ-producing T cells (Mean, n=2 independent experiments) and the EC$_{50}$ is given for each TCR (color-coded according to panel **a**). **c**. The reactivity of KIF1B$_{S918F}$ TCR-transfected T cells against the autologous tumor cell line was assessed by flow cytometry and is shown as the fraction of 4-1BB upregulating CD8^{+} T cells (Mean±SD). The color-code of the different clones corresponds to that in panel **a**. For TCR #1, 4-1BB upregulation was assessed from n=2 wells recovered from an ELISpot and pooled in n=1 tube, for TCR #2, n=2 technical replicates were made and Mean ±SD is shown. Source data are provided as a Source Data file.

Supplementary Table 1 - Specificity, antigen sensitivity and structural profile of CD8 T-cell clones

Patient/HD code	Protein	HLA restriction	Epitope	SNV	ID Clone	Origin	EC_{50} (M)	pMHC-TCR $T_{1/2}$ (s)	Clonotype (TCR - CDR3β chain)
Mel1	Melan-A	HLA-A*02:01	EAAGIGILTV		1	PBL	3.66E-07	2.9	hTRBV20_CSARDVGLGIYEQYFG_hTRBJ02-7
					2	PBL	-	2.9	hTRBV13_CASSLDPSGSPNEQFFG_hTRBJ02-1
					3	PBL	8.47E-08	3.2	hTRBV20_CSARDVGLGIYEQYFG_hTRBJ02-7
					4	PBL	7.65E-08	3.1	hTRBV03-1_CASSQGDLAWIPTEAFFG_hTRBJ01-1
					5	PBL	2.54E-08	4.0	hTRBV13_CASSLDPSGSPNEQFFG_hTRBJ02-1
					6	PBL	1.02E-07	4.6	ND
					7	PBL	1.10E-08	3.4	hTRBV20_CSARDVGLGIYEQYFG_hTRBJ02-7
					8	TIL	4.03E-08	4.0	hTRBV20_CSASPGLAEQFFG_hTRBJ02-1
					9	TIL	3.15E-08	3.7	hTRBV14_CASSQDTGLSSYNEQFFG_hTRBJ02-1
					10	TIL	8.20E-10	4.4	hTRBV06-1_CASSELGLAGNEQFFG_hTRBJ02-1
					11	TIL	1.24E-07	4.0	hTRBV20_CSAERGLGQPQHFG_hTRBJ01-5
					12	TIL	3.59E-09	4.1	hTRBV19_CASTSGELGQPQHFG_hTRBJ01-5
					13	TIL	1.23E-07	3.6	hTRBV27_CASSLSGLAGVEQYFG_hTRBJ02-7
					14	TIL	1.28E-07	36.3	hTRBV19_CASKWGALMNTEAFFG_hTRBJ01-1
					15	TIL	7.68E-08	5.0	hTRBV27_CASSSLGATYEQYFG_hTRBJ02-7
					16	TIL	1.66E-09	3.7	hTRBV27_CASSWTSGSPSEQFFG_hTRBJ02-1
					17	TIL	1.17E-09	3.9	hTRBV27_CASSLFSGSSGELFFG_hTRBJ02-2
					18	TIL	5.33E-08	16.6	ND
					19	TIL	2.49E-07	3.1	hTRBV03-1_CASSQGSLAGSEQYFG_hTRBJ02-7
					20	TIL	6.81E-09	7.9	hTRBV28_CASRVQGLGQPQHFG_hTRBJ01-5
					21	TIL	1.00E-06	3.6	hTRBV06-1_CASSELGLAGNEQFFG_hTRBJ02-1
					22	TIL	6.47E-09	5.9	hTRBV06-1_CASSELGLAGNEQFFG_hTRBJ02-1
Mel2	MAGE A3	HLA-A*01:01	EVDPIGHLY		1	PBL	-	37.4	hTRBV12-3_CASSLDRATNEKLFFG_hTRBJ01-4
					2	PBL	-	47.8	hTRBV12-3_CASSLDRATNEKLFFG_hTRBJ01-4
					3	PBL	-	37.1	hTRBV12-3_CASSLDRATNEKLFFG_hTRBJ01-4
					4	PBL	-	46.8	hTRBV12-3_CASSLDRATNEKLFFG_hTRBJ01-4
Mel3	NBEA	HLA-B*07:02	LPQARRI**L**L	S2272L	1	PBL	1.03E-09	83.8	ND
					2	PBL	7.06E-09	-	ND
Mel4	GP100	HLA-A*02:01	ITDQVPFSV		1	PBL	9.55E-09	13.5	ND
					2	PBL	1.49E-09	18.0	ND
					3	PBL	3.59E-08	4.8	hTRBV19_CASSMGQLILGYEQYFG_hTRBJ02-7
					4	PBL	4.15E-09	8.8	hTRBV02_CASSELERLKVYNSPLHFG_hTRBJ01-6
					5	PBL	3.83E-09	3.5	hTRBV19_CASSMGQLILGYEQYFG_hTRBJ02-7
					6	PBL	-	5.6	hTRBV19_CASSMGQLILGYEQYFG_hTRBJ02-7
					7	PBL	-	5.2	hTRBV19_CASSMGQLILGYEQYFG_hTRBJ02-7
					8	PBL	-	9.4	hTRBV02_CASSELERLKVYNSPLHFG_hTRBJ01-6
					9	PBL	-	13.4	hTRBV19_CASSMGQLILGYEQYFG_hTRBJ02-7
					10	TIL	7.70E-11	82.1	ND
					11	TIL	8.89E-10	63.3	ND
					12	TIL	-	68.1	ND
					13	TIL	1.56E-10	83.6	ND
					14	TIL	-	70.8	hTRBV19_CASSITTSGGYEQYFG_hTRBJ02-7
Mel5	GP100	HLA-A*02:01	ITDQVPFSV		1	PBL	2.07E-07	6.4	hTRBV10-2_CASSYRGNSPLHFG_hTRBJ01-6
					2	PBL	5.35E-06	3.8	hTRBV19_CASSLRLAATIYNEQFFG_hTRBJ02-1
					3	PBL	7.33E-07	9.4	hTRBV19_CASSARGYASPLHFG_hTRBJ01-6
Mel6	MAGE A10	HLA-A*02:01	GLYDGMEHL		1	PBL	-	2.8	hTRBV02_CASIGLAKNIQYFG_hTRBJ02-4
					2	PBL	-	2.2	hTRBV11-1_CASSFGGSSYEQYFG_hTRBJ02-7
					3	PBL	-	3.3	hTRBV09_CASSPVWAGAYNEQFFG_hTRBJ02-1
					4	PBL	-	2.7	hTRBV09_CASSPVWAGAYNEQFFG_hTRBJ02-1
					5	PBL	-	2.8	hTRBV09_CASSPVWAGAYNEQFFG_hTRBJ02-1
					6	PBL	-	2.1	hTRBV02_CASIGLAKNIQYFG_hTRBJ02-4
					7	PBL	-	2.3	hTRBV02_CASIGLAKNIQYFG_hTRBJ02-4
					8	PBL	-	2.7	hTRBV11-1_CASSFGGSSYEQYFG_hTRBJ02-7
					9	PBL	9.92E-08	2.8	ND
					10	PBL	1.34E-07	2.2	hTRBV02_CASIGLAKNIQYFG_hTRBJ02-4
					11	PBL	8.56E-10	2.5	ND
					12	PBL	6.92E-10	2.3	hTRBV11-1_CASSFGGSSYEQYFG_hTRBJ02-7
					13	PBL	1.67E-09	2.7	hTRBV09_CASSPVWAGAYNEQFFG_hTRBJ02-1
					14	PBL	1.03E-08	2.8	hTRBV09_CASSPVWAGAYNEQFFG_hTRBJ02-1
					15	PBL	6.81E-08	2.4	hTRBV11-1_CASSFGGSSYEQYFG_hTRBJ02-7
					16	PBL	2.69E-09	2.3	hTRBV11-1_CASSFGGSSYEQYFG_hTRBJ02-7
					17	PBL	1.12E-07	1.6	ND
CRC1	PHLPP2	HLA-A*01:01	QSDNGLDSD**Y**[2]	D1186Y	1	PBL	3.14E-08	43.3	hTRBV05-4_CASTLSTGQGIYGYTFG_hTRBJ01-2
					2	PBL	6.02E-08	53.2	hTRBV05-4_CASTLSTGQGIYGYTFG_hTRBJ01-2
					3	PBL	1.25E-07	43.9	hTRBV05-4_CASTLSTGQGIYGYTFG_hTRBJ01-2
					4	PBL	9.67E-08	42.6	ND
					5	PBL	1.06E-06	46.3	hTRBV05-4_CASTLSTGQGIYGYTFG_hTRBJ01-2
					6	PBL	6.76E-07	48.5	hTRBV05-4_CASTLSTGQGIYGYTFG_hTRBJ01-2
					7	PBL	1.53E-07	48.9	ND
					8	PBL	-	51.8	hTRBV05-4_CASTLSTGQGIYGYTFG_hTRBJ01-2
					9	PBL	-	55.7	ND
					10	TIL	5.12E-09	76.4	hTRBV10-3_CAISGGSVGEQYFG_hTRBJ02-7
					11	TIL	1.09E-08	75.2	hTRBV10-3_CAISGGSVGEQYFG_hTRBJ02-7
					12	TIL	4.44E-09	80.2	ND
					13	TIL	8.75E-09	75.8	ND
					14	TIL	-	48.5	ND
					15	TIL	-	65.4	hTRBV10-3_CAISGGSVGEQYFG_hTRBJ02-7
					16	TIL	-	64.4	hTRBV10-3_CAISGGSVGEQYFG_hTRBJ02-7
					17	TIL	-	77.4	ND
					18	TIL	-	56.2	ND

CRC1	PHLPP2	HLA-A*01:01	QSDNGLDSD**Y**[2]	D1186Y	19	TIL	4.45E-09	69.0	hTRBV10-3_CAISGGSVGEQYFG_hTRBJ02-7
					20	TIL	-	62.2	hTRBV10-3_CAISGGSVGEQYFG_hTRBJ02-7
					21	TIL	-	51.6	hTRBV10-3_CAISGGSVGEQYFG_hTRBJ02-7
					22	TIL	-	63.1	hTRBV10-3_CAISGGSVGEQYFG_hTRBJ02-7
					23	TIL	-	83.7	hTRBV10-3_CAISGGSVGEQYFG_hTRBJ02-7
					24	TIL	-	63.9	hTRBV10-3_CAISGGSVGEQYFG_hTRBJ02-7
					25	TIL	-	84.6	ND
					26	TIL	-	66.4	hTRBV10-3_CAISGGSVGEQYFG_hTRBJ02-7
					27	TIL	-	59.3	hTRBV10-3_CAISGGSVGEQYFG_hTRBJ02-7
					28	TIL	-	75.1	hTRBV10-3_CAISGGSVGEQYFG_hTRBJ02-7
					29	TIL	-	60.3	hTRBV10-3_CAISGGSVGEQYFG_hTRBJ02-7
					30	TIL	8.30E-09	90.6	hTRBV10-3_CAISGGSVGEQYFG_hTRBJ02-7
					31	TIL	-	58.2	hTRBV10-3_CAISGGSVGEQYFG_hTRBJ02-7
					32	TIL	-	84.2	ND
					33	TIL	-	80.6	ND
					34	TIL	9.25E-09	52.7	hTRBV10-3_CAISGGSVGEQYFG_hTRBJ02-7
					35	TIL	-	91.2	ND
					36	TIL	4.95E-09	84.8	hTRBV10-3_CAISGGSVGEQYFG_hTRBJ02-7
					37	TIL	1.39E-09	75.5	hTRBV10-3_CAISGGSVGEQYFG_hTRBJ02-7
					38	TIL	5.35E-09	86.4	hTRBV10-3_CAISGGSVGEQYFG_hTRBJ02-7
					39	TIL	3.58E-09	90.8	ND
CRC2	NUP210	HLA-A*02:01	GLQAILVH**V**	E849V	1	PBL	1.00E-09	34.5	ND
					2	PBL	-	29.2	ND
					3	PBL	3.49E-10	51.8	ND
					4	PBL	9.94E-11	30.4	ND
					5	PBL	3.27E-09	35.6	ND
					6	PBL	-	29.8	ND
					7	PBL	-	40.8	ND
					8	PBL	-	23.1	ND
					9	PBL	-	34.4	ND
					10	PBL	-	29.3	ND
					11	PBL	-	46.8	ND
					12	PBL	-	33.2	ND
					13	PBL	-	45.4	ND
OvCa1	HHAT	HLA-A*02:01	KQWLVWL**F**L[1]	L75F	1	PBL	5.80E-07	7.5	hTRBV04-2_CASSQDAETQYFG_hTRBJ02-5
					2	PBL	1.00E-07	3.0	hTRBV04-2_CASSQDAETQYFG_hTRBJ02-5
					3	PBL	1.40E-07	9.5	hTRBV04-2_CASSQDAETQYFG_hTRBJ02-5
					4	TIL	1.44E-08	20.5	hTRBV12-3_CASSRTSPTDTQYFG_hTRBJ02-3
					5	TIL	8.18E-09	31.8	hTRBV12-3_CASSRTSPTDTQYFG_hTRBJ02-3
					6	TIL	1.45E-08	-	ND
					7	TIL	1.51E-08	-	ND
					8	TIL	3.50E-09	-	ND
					9	TIL	9.29E-09	-	ND
					10	TIL	7.03E-09	-	ND
					11	TIL	9.87E-09	-	ND
					12	TIL	1.72E-09	-	ND
					13	TIL	3.52E-09	-	ND
					14	TIL	2.68E-09	-	ND
					15	TIL	2.34E-09	-	ND
					16	TIL	5.46E-09	-	ND
					17	TIL	5.02E-09	-	ND
					18	TIL	3.52E-09	-	ND
					19	TIL	4.26E-09	-	ND
OvCa2	ZCCHC11	HLA-B*27:05	GRKLFGT**H**F[1]	P1265H	1	PBL	8.73E-09	-	ND
					2	PBL	2.01E-08	4.0	hTRBV07-8_CASSWDSGYEQYFG_hTRBJ02-7
					3	PBL	3.66E-08	-	ND
					4	PBL	1.52E-08	3.7	hTRBV07-8_CASSWDSGYEQYFG_hTRBJ02-7
					5	PBL	1.79E-08	3.9	hTRBV07-8_CASSWDSGYEQYFG_hTRBJ02-7
					6	PBL	1.37E-08	3.6	hTRBV07-8_CASSWDSGYEQYFG_hTRBJ02-7
					7	PBL	1.03E-08	3.8	hTRBV07-8_CASSWDSGYEQYFG_hTRBJ02-7
					8	PBL	2.08E-09	-	ND
					9	TIL	2.94E-08	153.6	hTRBV07-8_CASSLDIGTYEQFFG_hTRBJ02-1
					10	TIL	7.74E-08	-	hTRBV28_CASSLAGLNTEAFFG_hTRBJ01-1
					11	TIL	1.15E-07	-	ND
OvCa3	SCL25A48	HLA-A*24:02	PY**M**FLSEWI	V200M	1	PBL	7.90E-07	2.6	hTRBV19_CASSIARVTEAFFG_hTRBJ01-1
					2	PBL	1.02E-09	61.4	hTRBV19_CASSIGKTGKLFFG_hTRBJ01-4
OvCa4	MAGE A1	HLA-A*02:01	KVLEYVIKV		1	PBL	-	9.0	ND
OvCa5	MUC1	HLA-A*02:01	VLVCVLVAL		1	PBL	3.20E-08	43.1	hTRBV07-3_CASSVGSYNEQFFG_hTRBJ02-1
					2	PBL	1.20E-08	39.3	hTRBV07-3_CASSVGSYNEQFFG_hTRBJ02-1
					3	PBL	3.90E-09	62.2	ND
					4	PBL	8.90E-09	40.1	hTRBV07-3_CASSVGSYNEQFFG_hTRBJ02-1
					5	PBL	5.40E-08	39.2	hTRBV07-3_CASSVGSYNEQFFG_hTRBJ02-1
Lung1	MMP9	HLA-A*02:01	NI**L**DAIAEI	F621L	1	PBL	4.24E-07	12.0	ND
					2	PBL	-	12.7	ND
					3	PBL	-	7.9	ND
					4	PBL	4.81E-07	6.4	hTRBV05-4_CASFFSGGGTDTQYFG_hTRBJ02-3
					5	PBL	-	5.9	hTRBV20_CSASRADTYEQYFG_hTRBJ02-7
					6	PBL	-	29.6	hTRBV07-2_CASTDTDTQYFG_hTRBJ02-3
					7	PBL	3.41E-07	9.9	hTRBV05-4_CASFFSGGGTDTQYFG_hTRBJ02-3
					8	PBL	-	12.7	hTRBV10-2_CASSLGQETQYFG_hTRBJ02-5
					9	PBL	4.22E-07	10.7	ND
					10	PBL	-	6.4	ND
					11	TIL	1.79E-09	50.8	hTRBV07-9_CASSPIAGGTDTQYFG_hTRBJ02-3

Lung1	MMP9	HLA-A*02:01	NILDAIAEI	F621L	12	TIL	9.97E-09	17.7	ND
					13	TIL	4.94E-10	50.9	hTRBV07-9_CASSPIAGGTDTQYFG_hTRBJ02-3
					14	TIL	1.04E-09	16.3	ND
					15	TIL	1.18E-09	25.1	ND
					16	TIL	2.71E-09	41.0	hTRBV07-9_CASSPIAGGTDTQYFG_hTRBJ02-3
					17	TIL	1.80E-08	-	ND
					18	TIL	4.80E-09	-	ND
					19	TIL	1.28E-08	-	ND
					20	TIL	9.26E-09	-	ND
					21	TIL	7.79E-09	-	hTRBV05-6_CASSLGGGRDEQYFG_hTRBJ02-7
					22	TIL	1.24E-08	-	ND
Lung1	UTP20	HLA-A*02:01	AMDLGI**H**KV	D2661H	1	PBL	7.21E-10	10.0	hTRBV10-1_CASSDSTAKETQYFG_hTRBJ02-5
					2	PBL	2.26E-08	61.2	hTRBV04-3_CASSQEESYEQYFG_hTRBJ02-7
					3	PBL	2.01E-09	54.7	hTRBV05-6_CASSLGGGRDTQYFG_hTRBJ02-3
					4	PBL	4.55E-09	-	ND
					5	TIL	7.20E-10	77.7	hTRBV11-1_CASSFQTGWNEQFFG_hTRBJ02-1
					6	TIL	-	88.8	ND
Lung2	Influenza A MP	HLA-A*02:01	GILGFVFTL		1	PBL	-	293.0	ND
					2	PBL	-	138.5	ND
					3	PBL	2.39E-10	-	ND
Lung3	EBV BMLF1	HLA-A*02:01	GLCTLVAML		1	PBL	-	27.6	ND
					2	PBL	-	44.6	ND
					3	PBL	-	27.3	ND
					4	PBL	-	18.2	ND
					5	PBL	4.96E-09	18.0	ND
					6	PBL	8.48E-09	21.3	ND
					7	PBL	1.54E-09	25.4	ND
					8	PBL	2.30E-09	21.0	ND
					9	PBL	6.84E-09	33.7	ND
					10	PBL	9.12E-09	20.5	ND
					11	PBL	6.41E-09	25.8	ND
					12	PBL	8.87E-09	17.3	ND
					13	PBL	4.22E-09	22.8	ND
					14	PBL	2.00E-09	16.1	ND
					15	PBL	1.24E-09	31.5	ND
					16	PBL	8.56E-11	71.8	ND
					17	PBL	2.25E-09	42.6	ND
					18	PBL	4.93E-10	66.2	ND
					19	PBL	-	33.8	ND
					20	PBL	-	21.5	ND
					21	PBL	-	47.9	ND
					22	TIL	6.21E-10	85.8	ND
					23	TIL	-	71.2	ND
					24	TIL	-	60.3	ND
					25	TIL	-	64.5	ND
					26	TIL	9.65E-10	87.4	ND
					27	TIL	7.41E-10	99.8	ND
					28	TIL	-	77.8	ND
					29	TIL	-	69.7	ND
					30	TIL	3.16E-09	56.3	ND
HD1	Influenza A PB1	HLA-A*01:01	VSDGGPNLY		1	PBL	1.15E-07	9.0	hTRBV19_CATSGRSGDTQYFG_hTRBJ02-3
					2	PBL	9.78E-08	20.9	hTRBV11-2_CASSLDGQGPLYGYTFG_hTRBJ01-2
					3	PBL	-	20.4	hTRBV19_CASSTRSSYEQYFG_hTRBJ02-7
HD2	Influenza A MP	HLA-A*02:01	GILGFVFTL		1	PBL	9.10E-13	83.7	ND
					2	PBL	-	72.6	ND
					3	PBL	-	86.5	ND
					4	PBL	-	54.1	ND
					5	PBL	-	19.9	ND
					6	PBL	-	47.2	ND
					7	PBL	-	101.3	ND
					8	PBL	8.90E-13	82.0	ND
					9	PBL	-	85.0	ND
					10	PBL	-	83.3	ND
					11	PBL	4.40E-12	69.3	ND
					12	PBL	2.80E-13	89.1	ND
					13	PBL	8.00E-12	72.2	ND
					14	PBL	-	87.0	ND
					15	PBL	-	66.6	ND
					16	PBL	-	38.1	ND
					17	PBL	-	119.7	ND
					18	PBL	-	66.7	ND
					19	PBL	-	53.9	ND
					20	PBL	-	70.1	ND
					21	PBL	-	62.1	ND
					22	PBL	-	61.8	ND
					23	PBL	-	50.2	ND
					24	PBL	-	83.7	ND
					25	PBL	-	64.1	ND
					26	PBL	-	191.2	ND
					27	PBL	-	74.3	ND
					28	PBL	-	71.4	ND
HD3	EBV BMLF1	HLA-A*02:01	GLCTLVAML		1	PBL	7.40E-09	102.7	hTRBV07-3_CASSPGGQSTDTQYFG_hTRBJ02-3
					2	PBL	-	81.3	hTRBV06-8_CASSENVGIGANVLTFG_hTRBJ02-6

					3	PBL	-	68.5	hTRBV06-8_CASSENVGIGANVLTFG_hTRBJ02-6
					4	PBL	-	108.4	ND
					5	PBL	2.78E-09	98.6	hTRBV07-3_CASSPGGQSTDTQYFG_hTRBJ02-3
					6	PBL	-	95.4	hTRBV06-8_CASSENVGIGANVLTFG_hTRBJ02-6
					7	PBL	-	62.5	hTRBV06-8_CASSENVGIGANVLTFG_hTRBJ02-6
					8	PBL	-	67.1	hTRBV06-8_CASSENVGIGANVLTFG_hTRBJ02-6
					9	PBL	3.10E-10	79.2	hTRBV06-8_CASSENVGIGANVLTFG_hTRBJ02-6
					10	PBL	3.52E-10	90.3	hTRBV06-8_CASSENVGIGANVLTFG_hTRBJ02-6
					11	PBL	-	80.1	hTRBV06-8_CASSENVGIGANVLTFG_hTRBJ02-6
					12	PBL	-	65.0	hTRBV06-8_CASSENVGIGANVLTFG_hTRBJ02-6
					13	PBL	1.66E-10	81.2	hTRBV07-3_CASSPGGQSTDTQYFG_hTRBJ02-3
					14	PBL	-	80.2	ND
					15	PBL	1.05E-08	15.5	hTRBV20_CSARDRGLGNTIYFG_hTRBJ01-3
					16	PBL	5.25E-09	88.5	hTRBV06-8_CASSENVGIGANVLTFG_hTRBJ02-6
					17	PBL	9.58E-10	71.0	hTRBV07-3_CASSPGGQSTDTQYFG_hTRBJ02-3
					18	PBL	6.37E-09	15.7	hTRBV20_CSARDRGLGNTIYFG_hTRBJ01-3
					19	PBL	2.10E-09	109.4	ND
					20	PBL	1.19E-08	17.1	hTRBV20_CSARDRGLGNTIYFG_hTRBJ01-3
					21	PBL	1.05E-10	61.7	hTRBV07-3_CASSPGGQSTDTQYFG_hTRBJ02-3
HD3	EBV BMLF1	HLA-A*02:01	GLCTLVAML		22	PBL	-	185.3	ND
					23	PBL	4.53E-11	68.4	ND
					24	PBL	1.37E-10	68.3	hTRBV07-3_CASSPGGQSTDTQYFG_hTRBJ02-3
					25	PBL	3.18E-10	108.6	ND
					26	PBL	-	74.3	ND
					27	PBL	-	76.2	hTRBV06-8_CASSENVGIGANVLTFG_hTRBJ02-6
					28	PBL	-	88.4	hTRBV06-8_CASSENVGIGANVLTFG_hTRBJ02-6
					29	PBL	1.77E-10	101.5	hTRBV07-3_CASSPGGQSTDTQYFG_hTRBJ02-3
					30	PBL	-	91.5	ND
					31	PBL	-	89.8	ND
					32	PBL	-	62.2	hTRBV06-8_CASSENVGIGANVLTFG_hTRBJ02-6
					33	PBL	-	69.7	ND
					34	PBL	-	76.8	hTRBV06-8_CASSENVGIGANVLTFG_hTRBJ02-6
					35	PBL	-	53.9	ND
					36	PBL	-	52.4	ND
					37	PBL	-	57.1	ND
					38	PBL	-	62.9	hTRBV06-8_CASSENVGIGANVLTFG_hTRBJ02-6
					39	PBL	7.40E-09	84.5	hTRBV06-8_CASSENVGIGANVLTFG_hTRBJ02-6
					40	PBL	-	56.9	ND
					41	PBL	-	89.3	hTRBV06-8_CASSENVGIGANVLTFG_hTRBJ02-6
					1	PBL	1.15E-11	102.3	hTRBV7-6_CASSLAPGATNEKLFFG_hTRBJ1-4
					2	PBL	1.10E-10	111.3	hTRBV27_CASSLNGGLPETQYFG_hTRBJ2-5
					3	PBL	1.12E-11	106.2	hTRBV27_CASSLNGGLPETQYFG_hTRBJ2-5
					4	PBL	4.39E-11	92.9	ND
					5	PBL	6.06E-11	93.1	ND
HD4	hCMV pp65	HLA-A*02:01	NLVPMVATV		6	PBL	9.26E-11	109.1	hTRBV27_CASSLNGGLPETQYFG_hTRBJ2-5
					7	PBL	-	85.8	ND
					8	PBL	4.90E-11	99.4	hTRBV20_CSARDNTVANYGYTFG_hTRBJ1-2
					9	PBL	-	100.6	hTRBV20_CSARDNTVANYGYTFG_hTRBJ1-2
					10	PBL	1.31E-11	93.8	ND
					11	PBL	5.89E-11	95.5	hTRBV20_CSARDNTVANYGYTFG_hTRBJ1-2
					1	PBL	6.73E-10	227.8	hTRBV05-4_CASSSLATSTDTQYFG_hTRBJ02-3
					2	PBL	6.12E-10	241.4	hTRBV05-4_CASSSLATSTDTQYFG_hTRBJ02-3
					3	PBL	9.09E-09	142.5	hTRBV05-4_CASSSLATSTDTQYFG_hTRBJ02-3
					4	PBL	4.80E-10	300.2	hTRBV05-4_CASSSLATSTDTQYFG_hTRBJ02-3
					5	PBL	5.14E-10	294.5	hTRBV04-1_CASSQDGTNYGYTFG_hTRBJ01-2
					6	PBL	2.19E-10	226.2	hTRBV05-4_CASSSLATSTDTQYFG_hTRBJ02-3
HD5	hCMV pp65	HLA-B*35:01	IPSINVHHY		7	PBL	2.69E-09	171.0	hTRBV02_CASSEEETGGSPLHFG_hTRBJ01-6
					8	PBL	7.76E-10	211.9	hTRBV02_CASMGGAYNEQFFG_hTRBJ02-1
					9	PBL	3.17E-10	201.1	hTRBV05-4_CASSSLATSTDTQYFG_hTRBJ02-3
					10	PBL	5.31E-09	-	ND
					11	PBL	1.69E-10	-	ND
					12	PBL	2.02E-09	-	ND
					13	PBL	3.41E-10	-	ND
					14	PBL	1.55E-10	-	ND
					1	PBL	-	111.0	ND
					2	PBL	-	20.6	ND
					3	PBL	-	16.7	ND
					4	PBL	-	17.6	ND
					5	PBL	-	19.4	ND
					6	PBL	-	27.4	ND
					7	PBL	-	18.5	ND
					8	PBL	-	29.1	ND
					9	PBL	-	25.8	ND
HD6	EBV BMLF1	HLA-A*02:01	GLCTLVAML		10	PBL	-	17.3	ND
					11	PBL	-	33.3	ND
					12	PBL	-	21.5	ND
					13	PBL	-	35.0	ND
					14	PBL	-	46.8	ND
					15	PBL	-	26.9	ND
					16	PBL	-	85.5	ND
					17	PBL	-	24.1	ND
					18	PBL	-	55.5	ND
					19	PBL	-	17.3	ND

					20	PBL	-	19.9	ND
					21	PBL	-	20.2	ND
					22	PBL	-	22.8	ND
					23	PBL	-	19.3	ND
					24	PBL	-	74.7	ND
					25	PBL	-	26.2	ND
					26	PBL	-	21.4	ND
					27	PBL	-	21.6	ND
					28	PBL	-	31.7	ND
					29	PBL	-	31.7	ND
					30	PBL	-	20.6	ND
					31	PBL	-	20.5	ND
					32	PBL	-	35.9	ND
					33	PBL	-	30.5	ND
					34	PBL	-	21.6	ND
					35	PBL	-	27.6	ND
					36	PBL	-	32.4	ND
					37	PBL	-	35.9	ND
					38	PBL	-	22.7	ND
					39	PBL	-	19.2	ND
					40	PBL	-	16.9	ND
HD6	EBV BMLF1	HLA-A*02:01	GLCTLVAML		41	PBL	-	17.5	ND
					42	PBL	-	4.3	ND
					43	PBL	-	19.4	ND
					44	PBL	-	17.7	ND
					45	PBL	-	19.0	ND
					46	PBL	-	16.7	ND
					47	PBL	-	17.5	ND
					48	PBL	-	23.3	ND
					49	PBL	-	9.6	ND
					50	PBL	-	26.4	ND
					51	PBL	-	24.8	ND
					52	PBL	-	108.8	ND
					53	PBL	-	21.6	ND
					54	PBL	-	19.9	ND
					55	PBL	-	34.5	ND
					56	PBL	-	20.3	ND
					57	PBL	-	30.3	ND
					58	PBL	-	39.0	ND
					59	PBL	-	27.6	ND
					60	PBL	-	24.6	ND
					61	PBL	-	20.0	ND

[1] Immunogenicity against the wild-type and mutated sequences previously reported in Bobisse *et al* , Sensitive and frequent identification of high avidity neo-epitope specific CD8$^+$ T cells in immunotherapy-naive ovarian cancer. *Nature Communications* (2018)[18].

[2] Immunogenicity against the wild-type and mutated sequences previously reported in Arnaud *et al.* , Sensitive identification of neoantigens and cognate TCRs in human solid tumors. *Nature Biotechnology* (2022)[19].

Supplementary Table 2 - Patients and donors description and clinical information

Code	Tumor type	Tumor stage	Sex	Treatment
Mel1	Melanoma	Advanced melanoma	M	naive
Mel2	Melanoma	Advanced melanoma	F	MAGE-A3 vaccination
Mel3	Melanoma	Advanced melanoma	M	Targeted therapy-Checkpoint blockade-Targeted therapy
Mel4	Melanoma	Advanced melanoma	M	naive
Mel5	Melanoma	Advanced melanoma	F	Lymphodepleting chemotherapy, MART-1 vaccination and ACT of PBMCs
Mel6	Melanoma	Advanced melanoma	M	Melan A and MART-1 peptide vaccines
Mel7	Melanoma	Advanced melanoma	M	Checkpoint blockade
Mel8	Melanoma	Advanced melanoma	M	Melan-A (ELA), MAGE-A10, NY-ESO1 peptide Vaccines-Checkpoint blockade
Mel9	Melanoma	Advanced melanoma	M	Targeted therapy-Checkpoint blockade-Targeted therapy
Mel10	Melanoma	Advanced melanoma	M	Chemotherapy-Checkpoint blockade
CRC1	Colorectal	Early-stage microsatellite instable colon adenocarcinoma	M	naive
CRC2	Colorectal	Early-stage microsatellite instable colon adenocarcinoma	F	naive
OvCa1	Ovarian	Advanced HGSOC	F	Chemotherapy, targeted therapy and dendritic cell vaccines
OvCa2	Ovarian	Advanced BRCA1 mutated HGSOC	F	Chemotherapy and dendritic cell vaccines
OvCa3	Ovarian	Advanced HGSOC	F	Chemotherapy and dendritic cell vaccines
OvCa4	Ovarian	Advanced HGSOC	F	Chemotherapy and dendritic cell vaccines
OvCa5	Ovarian	High grade serous ovarian carcinoma, BRCA1 mutated	F	Chemotherapy and targeted therapy
Lung1	NSCLC	Early-stage NSCLC squamous	M	naive
Lung2	NSCLC	Early-stage NSCLC adenocarcinoma	M	naive
Lung3	NSCLC	Early-stage NSCLC squamous	F	naive
**Code**	**Indication**		**Gender**	**Treatment**
HD1	healthy donor	NA	F (21 yo)	NA
HD2	healthy donor	NA	M (60 yo)	NA
HD3	healthy donor	NA	F (24 yo)	NA
HD4	healthy donor	NA	F (35 yo)	NA
HD5	healthy donor	NA	NA	NA
HD6	healthy donor	NA	NA	NA

Supplementary Table 3 - pMHC-TCR structural avidity and TCR pMHC interactions in the modeled complexes

Protein (patient)	peptide-MHC	ID Clone	TCRα/β	T1/2 (s)*	Number of predicted interactions			Data adjusted via equation
					polar	non-polar	total	
EBV (Lung3)	GLCTLVAML/HLA-A*02:01	Cl 14	hTRAV05_CAEDSNARLMFG_hTRAJ31<br>hTRBV20_CSARDRGLGNTIYFG_hTRBJ01-3	16,1	8	24	32	70,6
	GLCTLVAML/HLA-A*02:01	Cl 28	hTRAV30_CGTEGQMNTGFQKLVFG_hTRAJ08<br>hTRBV06-8_CASSENVGIGANVLTFG_hTRBJ02-6	77.8	8	32	40	91,8
ZCCHC11 (OvCa2)	GRKLFGTHF/HLA-B*27:05	Cl 9	hTRAV04_CLVGGPPTGNQFYFG_hTRAJ49<br>hTRBV07-8_CASSLDIGTYEQFFG_hTRBJ02-1	153,6	11	37	48	131,3
	GRKLFGTHF/HLA-B*27:05	Cl 7	hTRAV8-6_CAANNNNDMRFG_hTRAJ43<br>hTRBV7-8_CASSWDSGYEQYFG_hTRBJ02-7	3,8	4	13	17	6,5
UTP20 (Lung1)	AMDLGIHKV/HLA-A*02:01	Cl 5	hTRAV25_CAGMDSSYKLIFG_hTRAJ12<br>hTRBV11-1_CASSFQTGWNEQFFG_hTRBJ02-1	77,7	10	14	24	61,6
	AMDLGIHKV/HLA-A*02:01	Cl 1	hTRAV24_CAFINSGNTPLVFG_hTRAJ29<br>hTRBV10-1_CASSDSTAKETQYFG_hTRBJ02-5	10,0	5	11	16	10,0
MMP9 (Lung1)	NILDAIAEI/HLA-A*02:01	Cl 11	hTRAV12-312-4_CAMRSIGGSNYKLTFG_hTRAJ53<br>hTRBV07-9_CASSPIAGGTDTQYFG_hTRBJ02-3	48,1	7	16	23	40,7
	NILDAIAEI/HLA-A*02:01	Cl 7	hTRAV35_CAGHGNTGKLIFG_hTRAJ37<br>hTRBV05-4_CASFFSGGGTDTQYFG_hTRBJ02-3	9,9	4	11	15	1,2
GP100 (Mel4)	ITDQVPFSV/HLA-A*02:01	Cl 14	hTRAV24_CAFAELWGGSQGNLIFG_hTRAJ42<br>hTRBV19_CASSITTSGGYEQYFG_hTRBJ02-7	70,8	4	26	30	40,9
	ITDQVPFSV/HLA-A*02:01	Cl 8	hTRAV41_CASTNVGGSGNTPLVFG_hTRAJ29<br>hTRBV19_CASSARGYASPLHFG_hTRBJ01-6	9,4	4	19	23	22,4

*The mean half-life, T1/2 (s), was correlated with the number of non-polar, napolar, and the number of polar, npolar, contacts between TCR and pMHC via the equation below:

$$T_{\frac{1}{2}}(s) = K + \gamma \times n_{apolar} + \delta \times n_{polar}$$

Supplementary Table 4 - List of pMHC-TCR and their structural avidity used for clustering analysis

	Clone specificity	TCRα/β	peptide-MHC	$T_{1/2}$ (s)	ID TCR Model
Viral Antigens	EBV BMLF1 PBLs	hTRAV30_CGTEGQMNTGFQKLVFG_hTRAJ08<br>hTRBV06-8_CASSENVGIGANVLTFG_hTRBJ02-6	GLCTLVAML/HLA-A*02:01	77.8	1
		hTRAV05_CAEDSNARLMFG_hTRAJ31<br>hTRBV20_CSARDRGLGNTIYFG_hTRBJ01-3	GLCTLVAML/HLA-A*02:01	16.1	2
	hCMV pp65 PBLs	hTRAV24_CAFLTGTYKYIFG_hTRAJ40<br>hTRBV05-4_CASSSLATSTDTQYFG_hTRBJ02-3	IPSINVHHY/HLA-B*35:01	171.0	3
		hTRAV12-2_CAGYSGTYKYIFG_hTRAJ40<br>hTRBV02_CASMGGAYNEQFFG_hTRBJ02-1	IPSINVHHY/HLA-B*35:01	294.5	4
		hTRAV16_FNKFYFG_hTRAJ21<br>hTRBV02_CASSEEETGGSPLHFG_hTRBJ01-6	IPSINVHHY/HLA-B*35:01	211.9	5
		hTRAV22_CAGREVTGGGNKLTFG_hTRAJ10<br>hTRBV04-1_CASSQDGTNYGYTFG_hTRBJ01-2	IPSINVHHY/HLA-B*35:01	214.5	6
	Influenza A PB1 PBLs	hTRAV21_CAVINAGNNRKLIWG_hTRAJ38<br>hTRBV11-2_CASSLDGQGPLYGYTFG_hTRBJ01-2	VSDGGPNLY/HLA-A*01:01	16.8	8
	Influenza A PB1 TILs	hTRAV27_CAGAGSQGNLIFG_hTRAJ42<br>hTRBV19_CASSIRSSYEQYFG_hTRBJ02-7	VSDGGPNLY/HLA-A*01:01	293.0	9
		hTRAV27_CAGAGGGSQGNLIFG_hTRAJ42<br>hTRBV19_CASSIRSSNEQFFG_hTRBJ02-1	VSDGGPNLY/HLA-A*01:01	ND	10
		hTRAV04_CLNAGNNRKLIWG_hTRAJ38<br>hTRBV19_CASGLGLEQFFG_hTRBJ02-1	VSDGGPNLY/HLA-A*01:01	ND	11
TAAs	GP100 PBLs	hTRAV08-1_CAGGGDRDDKIIFG_hTRAJ30<br>hTRBV10-2_CASSYRGNSPLHFG_hTRBJ01-6	ITDQVPFSV/HLA-A*02:01	6.4	12
		hTRAV01-2_CAVPSYGQNFVFG_hTRAJ26<br>hTRBV19_CASSLRLAATIYNEQFFG_hTRBJ02-1	ITDQVPFSV/HLA-A*02:01	3.8	13
		hTRAV41_CASTNVGGSGNTPLVFG_hTRAJ29<br>hTRBV19_CASSARGYASPLHFG_hTRBJ01-6	ITDQVPFSV/HLA-A*02:01	9.4	14
		hTRAV19_CALSEGGGGADGLTFG_hTRAJ45<br>hTRBV02_CASSELERLKVYNSPLHFG_hTRBJ01-6	ITDQVPFSV/HLA-A*02:01	9.0	15
		hTRAV10_CVVSARSGGSYIPTFG_hTRAJ06<br>hTRBV19_CASSMGQLILGYEQYFG_hTRBJ02-7	ITDQVPFSV/HLA-A*02:01	6.2	16
	GP100 TILs	hTRAV24_CAFAELWGGSQGNLIFG_hTRAJ42<br>hTRBV19_CASSITTSGGYEQYFG_hTRBJ02-7	ITDQVPFSV/HLA-A*02:01	70.8	17
	Melan-A TILs	hTRAV12-2_CAGGGSNYQLIWGAG_hTRAJ33<br>hTRBV20_CSASPGLAEQFFG_hTRBJ02-1	EAAGIGILTV/HLA-A*02:01	4.0	18
		hTRAV12-2_CAVDVGARLMFG_hTRAJ31<br>hTRBV14_CASSQDTGLSSYNEQFFG_hTRBJ02-1	EAAGIGILTV/HLA-A*02:01	3.7	19
		hTRAV12-2_CAYQAGTALIFG_hTRAJ15<br>hTRBV06-1_CASSELGLAGNEQFFG_hTRBJ02-1	EAAGIGILTV/HLA-A*02:01	4.8	20
		hTRAV12-2_CAVNTGNQFYFG_hTRAJ49<br>hTRBV20_CSAERGLGQPQHFG_hTRBJ01-5	EAAGIGILTV/HLA-A*02:01	4.0	21
		hTRAV12-2_CAPGGGYQKVTFG_hTRAJ13<br>hTRBV19_CASTSGELGQPQHFG_hTRBJ01-5	EAAGIGILTV/HLA-A*02:01	4.1	22
		hTRAV12-2_CAVIHAGKSTFG_hTRAJ27<br>hTRBV27_CASSLSGLAGVEQYFG_hTRBJ02-7	EAAGIGILTV/HLA-A*02:01	3.6	23
		hTRAV35_CAGVLGSARQLTFG_hTRAJ22<br>hTRBV19_CASKWGALMNTEAFFG_hTRBJ01-1	EAAGIGILTV/HLA-A*02:01	36.3	24
		hTRAV12-2_CAASIGFGNVLHCGSG_hTRAJ35<br>hTRBV27_CASSSLGATYEQYFG_hTRBJ02-7	EAAGIGILTV/HLA-A*02:01	5.0	25
		hTRAV12-2_CAASIGFGNVLHCGSG_hTRAJ35<br>hTRBV27_CASSWTSGSPSEQFFG_hTRBJ02-1	EAAGIGILTV/HLA-A*02:01	3.7	26
		hTRAV12-2_CAVTIGFGNVLHCGSG_hTRAJ35<br>hTRBV27_CASSLFSGSSGELFFG_hTRBJ02-2	EAAGIGILTV/HLA-A*02:01	3.9	27
		hTRAV12-2_CAVGGGADGLTFG_hTRAJ45<br>hTRBV03-1_CASSQGSLAGSEQYFG_hTRBJ02-7	EAAGIGILTV/HLA-A*02:01	3.1	28
		hTRAV12-2_CAVGGAAGNKLTFG_hTRAJ17<br>hTRBV28_CASRVQGLGQPQHFG_hTRBJ01-5	EAAGIGILTV/HLA-A*02:01	7.9	29
	Melan-A PBLs	hTRAV12-2_CAVSSGFQKLVFG_hTRAJ08<br>hTRBV13_CASSLDPSGSPNEQFFG_hTRBJ02-1	EAAGIGILTV/HLA-A*02:01	3.4	30
		hTRAV12-2_CAVNDAGKSTFG_hTRAJ27<br>hTRBV03-1_CASSQGDLAWIPTEAFFG_hTRBJ01-1	EAAGIGILTV/HLA-A*02:01	3.1	31
		hTRAV14_CAMRGPYNTDKLIFG_hTRAJ34<br>hTRBV20_CSARDVGLGIYEQYFG_hTRBJ02-7	EAAGIGILTV/HLA-A*02:01	3.2	32
NeoAntigens	PHLPP2 TILs	hTRAV23_CAAPMPMDTGRRALTFG_hTRAJ05<br>hTRBV10-3_CAISGGSVGEQYFG_hTRBJ02-7	QSDNGLDSDY/HLA-A*01:01	69.3	36
	PHLPP2 PBLs	hTRAV21_CAVSSGSARQLTFG_hTRAJ22<br>hTRBV05-4_CASTLSTGQGIYGYTFG_hTRBJ01-2	QSDNGLDSDY/HLA-A*01:01	47.8	37
		hTRAV21_CAVGGSGSARQLTFG_hTRAJ22<br>hTRBV05-4_CASSPTTSGRIGELFFG_hTRBJ02-2	QSDNGLDSDY/HLA-A*01:01	<3s	69
	ZCCHC11 TILs	hTRAV04_CLVGGPPTGNQFYFG_hTRAJ49<br>hTRBV07-8_CASSLDIGTYEQFFG_hTRBJ02-1	GRKLFGTHF/HLA-B*27:05	153.6	38
		hTRAV21_CAVRLTGQGAQKLVFG_hTRAJ54<br>hTRBV28_CASSLAGLNTEAFFG_hTRBJ01-1	GRKLFGTHF/HLA-B*27:05	ND	39

NeoAntigens	**ZCCHC11 PBLs**	hTRAV08-6_CAANNNNDMRFG_hTRAJ43<br>hTRBV07-8_CASSWDSGYEQYFG_hTRBJ02-7	GRKLFGTHF/HLA-B*27:05	3.8	40
	**HHAT PBLs**	hTRAV12-2_CAVNYNNARLMFG_hTRAJ31<br>hTRBV04-2_CASSQDAETQYFG_hTRBJ02-5	KQWLVWLFL/HLA-A*02:01	6.7	41
	**HHAT TILs**	hTRAV38-2_CAFMDSNYQLIWGAG_hTRAJ33<br>hTRBV12-3_CASSRTSPTDTQYFG_hTRBJ02-3	KQWLVWLFL/HLA-A*02:01	26.2	43
	**UTP20 TILs**	hTRAV25_CAGMDSSYKLIFG_hTRAJ12<br>hTRBV11-1_CASSFQTGWNEQFFG_hTRBJ02-1	AMDLGIHKV/HLA-A*02:01	77.7	44
	**UTP20 PBLs**	hTRAV12-2_CAGGVDSNYQLIWGAG_hTRAJ33<br>hTRBV05-6_CASSLGGGRDEQYFG_hTRBJ02-7	AMDLGIHKV/HLA-A*02:01	ND	45
		hTRAV24_CAFINSGNTPLVFG_hTRAJ29<br>hTRBV10-1_CASSDSTAKETQYFG_hTRBJ02-5	AMDLGIHKV/HLA-A*02:01	10.0	46
		hTRAV20_CAVSGGSYIPTFG_hTRAJ06<br>hTRBV04-3_CASSQEESYEQYFG_hTRBJ02-7	AMDLGIHKV/HLA-A*02:01	61.2	47
		hTRAV19_CALIFNQAGTALIFG_hTRAJ15<br>hTRBV05-6_CASSLGGGRDTQYFG_hTRBJ02-3	AMDLGIHKV/HLA-A*02:01	52.7	48
	**MMP9 TILs**	hTRAV24_CAPNRDDKIIFG_hTRAJ30<br>hTRBV12-3_CASATGVKLAKNIQYFG_hTRBJ02-4	NILDAIAEI/HLA-A*02:01	ND	49
		hTRAV12-312-4_CAMRSIGGSNYKLTFG_hTRAJ53<br>hTRBV07-9_CASSPIAGGTDTQYFG_hTRBJ02-3	NILDAIAEI/HLA-A*02:01	48.1	50
	**MMP9 PBLs**	hTRAV35_CAGHGNTGKLIFG_hTRAJ37<br>hTRBV05-4_CASFFSGGGTDTQYFG_hTRBJ02-3	NILDAIAEI/HLA-A*02:01	9.9	51
		hTRAV12-2_CAVRGNEKLTFG_hTRAJ48<br>hTRBV20_CSASRADTYEQYFG_hTRBJ02-7	NILDAIAEI/HLA-A*02:01	5.8	52
		hTRAV13-1_CAASSMNRDDKIIFG_hTRAJ30<br>hTRBV07-2_CASTDTDTQYFG_hTRBJ02-3	NILDAIAEI/HLA-A*02:01	29.6	53
		hTRAV13-1_CAASINTDKLIFG_hTRAJ34<br>hTRBV05-4_CASFFSGGGTDTQYFG_hTRBJ02-3	NILDAIAEI/HLA-A*02:01	6.4	55
		hTRAV12-2_CAVGGTSYGKLTFG_hTRAJ52<br>hTRBV10-2_CASSLGQETQYFG_hTRBJ02-5	NILDAIAEI/HLA-A*02:01	12.6	57
		hTRAV27_CAGGNSGGYQKVTFG_hTRAJ13<br>hTRBV05-4_CASFFSGGGTDTQYFG_hTRBJ02-3	NILDAIAEI/HLA-A*02:01	ND	58
	**SLC25A48 PBLs**	hTRAV21_CAVPSTSGTYKYIFG_hTRAJ40<br>hTRBV19_CASSIGKTGKLFFG_hTRBJ01-4	PYMFLSEWI/HLA-A*24:02	62.0	59
		hTRAV17_CATGGALGYGGSQGNLIFG_hTRAJ42<br>hTRBV19_CASSIARVTEAFFG_hTRBJ01-1	PYMFLSEWI/HLA-A*24:02	3.6	60
		hTRAV12-1_CVVRANNARLMFG_hTRAJ31<br>hTRBV07-2_CASSTGSSGELFFG_hTRBJ02-2	PYMFLSEWI/HLA-A*24:02	4.1	65
	**FPR2 PBLs**	hTRAV12-2_CGGSGTASKLTFG_hTRAJ44<br>hTRBV05-1_CASSFSGSEQFFG_hTRBJ02-1	VFSFTATLPF/HLA-A*24:02	<3s	66
		hTRAV19_CALSEWELNTNAGKSTFG_hTRAJ27<br>hTRBV20_CSARKRGYREEAFFG_hTRBJ01-1	VFSFTATLPF/HLA-A*24:02	<3s	67
	**FPR2 TILs**	hTRAV20_CAVLSGNTGKLIFG_hTRAJ37<br>hTRBV20_CSARGQGNTEAFFG_hTRBJ01-1	VFSFTATLPF/HLA-A*24:02	<3s	68

Supplementary Table 5 - Amino acids frequencies in CDR3β for high (>60s) and low avidity TCRs used for clustering analyses. Cluster of high avidity TCRs is depicted as a black dashed box in **Figure 4b**.

Amino Acid	Inside box cluster	Outside box cluster	High-Affinity (> 60 s)	High-Affinity outside box cluster	High-Affinity within box cluster	Low-Affinity (< 60 s)
**A**	0,029	0,072	0,031	0,048	0,016	0,063
**R**	0,052	0,023	0,01	0	0,032	0,039
**N**	0,017	0,046	0,052	0,095	0,016	0,031
**D**	0,069	0,033	0,031	0,024	0,048	0,05
**C**	0	0	0	0	0	0
**Q**	0,092	0,128	0,115	0,119	0,113	0,115
**E**	0,075	0,112	0,135	0,119	0,129	0,089
**G**	0,201	0,155	0,177	0,19	0,194	0,17
**H**	0	0	0	0	0	0
**I**	0,023	0,039	0,052	0,071	0,032	0,029
**L**	0,075	0,105	0,052	0,048	0,065	0,105
**K**	0,023	0,01	0,021	0	0,032	0,013
**M**	0	0,007	0	0	0	0,005
**F**	0,017	0,01	0,01	0,024	0	0,013
**P**	0,04	0,03	0,01	0	0,016	0,039
**S**	0,086	0,082	0,083	0,024	0,113	0,084
**T**	0,121	0,076	0,104	0,095	0,113	0,089
**W**	0	0,016	0,01	0,024	0	0,01
**Y**	0,069	0,033	0,073	0,071	0,065	0,039
**V**	0,011	0,023	0,031	0,048	0,016	0,016

Amino acid frequencies in CDR3β (first 4 and last 3 residues removed - in agreement with the biophysical-based clustering analysis).

Supplementary References

1. Giudicelli, V. IMGT/GENE-DB: a comprehensive database for human and mouse immunoglobulin and T cell receptor genes. *Nucleic Acids Research* **33**, D256–D261 (2004).

2. Leaver-Fay, A. *et al.* ROSETTA3: an object-oriented software suite for the simulation and design of macromolecules. *Methods Enzymol* **487**, 545–574 (2011).

3. Rose, P. W. *et al.* The RCSB protein data bank: integrative view of protein, gene and 3D structural information. *Nucleic Acids Res* **45**, D271–D281 (2017).

4. Gowthaman, R. & Pierce, B. G. TCRmodel: high resolution modeling of T cell receptors from sequence. *Nucleic Acids Res* **46**, W396–W401 (2018).

5. Henikoff, S. & Henikoff, J. G. Amino acid substitution matrices from protein blocks. *Proc Natl Acad Sci U S A* **89**, 10915–10919 (1992).

6. Nivón, L. G., Moretti, R. & Baker, D. A Pareto-optimal refinement method for protein design scaffolds. *PLoS One* **8**, e59004 (2013).

7. Webb, B. & Sali, A. Comparative Protein Structure Modeling Using MODELLER. *Current Protocols in Protein Science* **86**, 2.9.1-2.9.37 (2016).

8. Mandell, D. J., Coutsias, E. A. & Kortemme, T. Sub-angstrom accuracy in protein loop reconstruction by robotics-inspired conformational sampling. *Nat Methods* **6**, 551–552 (2009).

9. Pettersen, E. F. *et al.* UCSF Chimera—A visualization system for exploratory research and analysis. *Journal of Computational Chemistry* **25**, 1605–1612 (2004).

10. Zoete, V., Cuendet, M. A., Grosdidier, A. & Michielin, O. SwissParam: A fast force field generation tool for small organic molecules. *Journal of Computational Chemistry* **32**, 2359–2368 (2011).

11. Bjerregaard, A.-M. *et al.* An Analysis of Natural T Cell Responses to Predicted Tumor Neoepitopes. *Front Immunol* **8**, 1566 (2017).

12. Schmidt, J. *et al.* Prediction of neo-epitope immunogenicity reveals TCR recognition determinants and provides insight into immunoediting. *Cell Reports Medicine* **2**, 100194 (2021).

13. O'Donnell, T. J., Rubinsteyn, A. & Laserson, U. MHCflurry 2.0: Improved Pan-Allele

Prediction of MHC Class I-Presented Peptides by Incorporating Antigen Processing. *Cell Syst* **11**, 42-48.e7 (2020).

14. Bassani-Sternberg, M. *et al.* Deciphering HLA-I motifs across HLA peptidomes improves neo-antigen predictions and identifies allostery regulating HLA specificity. *PLOS Computational Biology* **13**, e1005725 (2017).

15. Reynisson, B., Alvarez, B., Paul, S., Peters, B. & Nielsen, M. NetMHCpan-4.1 and NetMHCIIpan-4.0: improved predictions of MHC antigen presentation by concurrent motif deconvolution and integration of MS MHC eluted ligand data. *Nucleic Acids Res* **48**, W449–W454 (2020).

16. Rasmussen, M. *et al.* Pan-Specific Prediction of Peptide–MHC Class I Complex Stability, a Correlate of T Cell Immunogenicity. *The Journal of Immunology* **197**, 1517–1524 (2016).

17. Atchley, W. R., Zhao, J., Fernandes, A. D. & Drüke, T. Solving the protein sequence metric problem. *Proc Natl Acad Sci U S A* **102**, 6395–6400 (2005).

18. Bobisse, S. *et al.* Sensitive and frequent identification of high avidity neo-epitope specific CD8 + T cells in immunotherapy-naive ovarian cancer. *Nature Communications* **9**, (2018).

19. Arnaud, M. *et al.* Sensitive identification of neoantigens and cognate TCRs in human solid tumors. *Nat Biotechnol* 1–5 (2021) doi:10.1038/s41587-021-01072-6.
