# Supplementary material for: Neoantigen-specific CD8 T cells with high structural avidity preferentially reside in and eliminate tumors
Source: Nat Commun. 2023 Jun 6;14:3188. doi: 10.1038/s41467-023-38946-z (PMC10244384; doi:10.1038/s41467-023-38946-z)
Supplement: Supplementary file 2 — Reporting Summary [file 41467_2023_38946_MOESM2_ESM.pdf]

nature portfolio

Corresponding author(s): Pr. Alexandre Harari

Last updated by author(s): Mar 28, 2023

Reporting Summary

Nature Portfolio wishes to improve the reproducibility of the work that we publish. This form provides structure for consistency and transparency in reporting. For further information on Nature Portfolio policies, see our Editorial Policies and the Editorial Policy Checklist.

Statistics

For all statistical analyses, confirm that the following items are present in the figure legend, table legend, main text, or Methods section.

n/a	Confirmed	
☐	☒	The exact sample size (n) for each experimental group/condition, given as a discrete number and unit of measurement
☐	☒	A statement on whether measurements were taken from distinct samples or whether the same sample was measured repeatedly
☐	☒	The statistical test(s) used AND whether they are one- or two-sided<br>*Only common tests should be described solely by name; describe more complex techniques in the Methods section.*
☒	☐	A description of all covariates tested
☐	☒	A description of any assumptions or corrections, such as tests of normality and adjustment for multiple comparisons
☐	☒	A full description of the statistical parameters including central tendency (e.g. means) or other basic estimates (e.g. regression coefficient) AND variation (e.g. standard deviation) or associated estimates of uncertainty (e.g. confidence intervals)
☐	☒	For null hypothesis testing, the test statistic (e.g. F, t, r) with confidence intervals, effect sizes, degrees of freedom and P value noted<br>*Give P values as exact values whenever suitable.*
☒	☐	For Bayesian analysis, information on the choice of priors and Markov chain Monte Carlo settings
☐	☒	For hierarchical and complex designs, identification of the appropriate level for tests and full reporting of outcomes
☐	☒	Estimates of effect sizes (e.g. Cohen's d, Pearson's r), indicating how they were calculated

Our web collection on statistics for biologists contains articles on many of the points above.

Software and code

Policy information about availability of computer code

Data collection	Data were collected using software publicly available and stated in the method section.<br>For single-cell RNAseq and TCRseq: 10X Genomics.<br>For FACS sorting: BD FACS ARIA III, BD Melody Cell sorter.<br>For flow cytometry: BD LSRII, BD Fortessa, IntelliCyt iQue Screener Plus (Bucher Biotec).<br>For sequencing: MiniSeq, HiSeq 2500, HiSeq 4000 (Illumina)
Data analysis	The following available codes and software were used in this study.<br>PRIME 1.0 (https://github.com/GfellerLab/PRIME), MHCflurry 2.0 (https://github.com/openvax/MHCflurry), MixMHCpred 2.1 (https://github.com/GfellerLab/MixMHCpred), NetMHCpan 4.1 and NetMHCpan 3.0 (https://www.cbs.dtu.dk/services/NetMHCpan), NetMHCstabpan 1.0 (https://www.cbs.dtu.dk/services/NetMHCstabpan), Rosetta 3.10 (https://www.rosettacommons.org/software), Modeller 10.1 (https://salilab.org/modeller) and UCSF Chimera 1.14 (https://www.cgl.ucsf.edu/chimera).<br>For exome analysis: NeoDisc V1.2 (https://github.com/bassanilab/neodisc).<br>Graphical analysis: GraphPad Prim V7 and V9.<br>For single-cell RNAseq analysis: Cell Ranger V3.0.1, Seurat V3.2.2.<br>For single-cell TCRseq analysis: Cell Ranger V3.1.0.<br>For flow cytometry: FlowJo 10.5.3, FACS DIVA V9.0<br>For fluorescence imaging: PhenoChart, inForm v2.3.0

For manuscripts utilizing custom algorithms or software that are central to the research but not yet described in published literature, software must be made available to editors and reviewers. We strongly encourage code deposition in a community repository (e.g. GitHub). See the Nature Portfolio guidelines for submitting code & software for further information.

Data

Policy information about availability of data

All manuscripts must include a data availability statement. This statement should provide the following information, where applicable:

- Accession codes, unique identifiers, or web links for publicly available datasets
- A description of any restrictions on data availability
- For clinical datasets or third party data, please ensure that the statement adheres to our policy

The authors declare that additional data supporting the findings of this study are available within the article and its supplementary information. Other data are available from the corresponding author upon reasonnable request.

Human research participants

Policy information about studies involving human research participants and Sex and Gender in Research.

Reporting on sex and gender	No bias induced by gender or age were present in the enrolled patients. No bias induced by gender or age were present in healthy donors.
Population characteristics	All patients received several lines of chemotherapy. Patients included stage III/IV metastatic melanoma, ovarian, non-small cell lung cancer and colorectal cancer (described in Table S2). Anonymous healthy donors were enrolled without any restrictions of gender or age.
Recruitment	Patients were enrolled under protocols approved by the respective institutional regulatory committees at the University of Pennsylvania (USA) and Lausanne University Hospital (Switzerland) without any restrictions or induced selection-biases .
Ethics oversight	Samples were collected and biobanked from patients enrolled under protocols approved by the respective institutional regulatory committees at the University of Pennsylvania, USA, and Lausanne university hospital (CHUV), Switzerland. Patients recruitment, study procedures, and blood withdrawal were approved by regulatory authorities and all patients signed written informed consents. Collection from healthy donors followed legal Swiss guidelines under the project P_123 with informed consent and with Ethics Approval from the Canton de Vaud (Switzerland).

Note that full information on the approval of the study protocol must also be provided in the manuscript.

Field-specific reporting

Please select the one below that is the best fit for your research. If you are not sure, read the appropriate sections before making your selection.

[x] Life sciences [ ] Behavioural & social sciences [ ] Ecological, evolutionary & environmental sciences

For a reference copy of the document with all sections, see nature.com/documents/nr-reporting-summary-flat.pdf

Life sciences study design

All studies must disclose on these points even when the disclosure is negative.

Sample size	All in vitro studies were performed according to sample availability.No sample-size calculation was performed but all clones generated by sorting and limiting dilution were used providing most of the time enough data for statistical significance.<br>For in vivo studies, 2 to 5 millions CD8 T cells/mouse were used. We achieved a sample size of minimum 5 animals per treatment group which proved to be sufficient to reproducibly observe statistically significant differences. The number of animals per group was based on calculations via the following website: https://www.openepi.com/samplesize/SSMean.htm.
Data exclusions	No data were excluded in this study.
Replication	All attempts at replication were successful. Each in vitro experiment was replicated 3 times. Each in vivo experiment was replicated twice except the ACT with neoantigen-specific CD8 T cells that was a single experiment asked fo revision of the manuscript in a limited period of time. However results were statistically significant.
Randomization	For in vivo experiment, tumor burden was evaluated by calipering the same day of T cell transfer and mice were then assigned in experimental groups randomly. Each group had the same overall average tumor volume.<br>Apheresis were obtained from anonymous healthy donors. Blood and tumor samples were obtained from anonimized patients.<br>For in vitro experiments, randomization was not applicable.
Blinding	To minimize stress to mice, treatment administration and tumor volume measurements were done at the same time (during same anaesthesia). An independent investigator verified caliper measurements in a blinded fashion. Analysis of data (plotting of pre-recorded tumor volumes at end of study) was performed in a non-blinded manner. A complete blinding was not achieved because it would have required additional operators.

For in vitro experiments, the blinding concept is not applicable.

Reporting for specific materials, systems and methods

We require information from authors about some types of materials, experimental systems and methods used in many studies. Here, indicate whether each material, system or method listed is relevant to your study. If you are not sure if a list item applies to your research, read the appropriate section before selecting a response.

Materials & experimental systems

n/a	Involved in the study
☐	☒ Antibodies
☐	☒ Eukaryotic cell lines
☒	☐ Palaeontology and archaeology
☐	☒ Animals and other organisms
☒	☐ Clinical data
☒	☐ Dual use research of concern

Methods

n/a	Involved in the study
☒	☐ ChIP-seq
☐	☒ Flow cytometry
☒	☐ MRI-based neuroimaging

Antibodies

Antibodies used	The following antibodies were used for immunofluorescence labeling: anti-hCD8 antibody (CellMarque, clone SP16, cat# 108R), anti-SOX10 antibody (CellMarque, clone EP261, cat# 383R), anti rabbit HRP antibody (Ventana, #760-4311), anti mouse HRP (Ventana, #760-4310)<br>The following antibodies were used for flow cytometry: anti-hCD8 (Biolegend, clone SK1, cat# 980906 or BDBioscience clone RPA-T8, cat# 558207), anti-4-1BB (Miltenyi, #4B4-1, cat# 130-093-475), anti-CD3 (Biolegend, clone SK7, cat# 981006 or Invitrogen, clone 7D6, cat# MHCD0301), anti-hCD4 (BDBioscience, RPA-T4, cat# 562281), anti-mouse TCRb-constant (Thermofishcer Scientific, clone H57-597, cat# 17-5961-81), anti-CXCR1 (Biolegend, clone 2A9-1, cat# 341625), anti-CD194 (Biolegend, clone L291H4, cat# 359417), anti-CD197 (Biolegend, clone G043H7, cat# 353227), anti-CD49b (Biolegend, clone P1E6, cat# 359305), anti-CD195 (Biolegend, clone HEK/1/85a, cat# 313715), anti-CD196 (Biolegend, clone G034E3, cat# 353425), anti-CD49a (Biolegend, clone TS2/7, cat# 328303), anti-CD103 (Biolegend, clone Ber-ACT8, cat# 350211), anti-CD183 (Biolegend, clone G025H7, cat# 353725).<br>For in vivo the following antibodies were used: anti CXCR3 (Biolegend, clone G025H7, cat# 353749) and isotype control (Biolegend, clone MOPC-21, cat# 400166).<br>For stimulation: anti CD3 (Biolegend, clone OKT3, cat# 317302).
Validation	Each antibody was used according to the manufacturer's instructions (www.biolegend.com, www.cellmarque.com, www.bdbiosciences.com) and optimally titrated. Validation at Biolegend is done by testing antibodies on multiple cell and tissue types with a variety of know expression levels and using cell treatment to modulate target expression when possible. Antibody validation at BDBiosciences is performed using multiple methodologies including flow cytometry, immunofluorescence, immunohistochemistry or western blot to test staining on a combination of primary cells, cell lines or transfectant models. Validation of antibodies for immunofluorescence labeling was performed in-house using tumors and PBMCs samples.

Eukaryotic cell lines

Policy information about cell lines and Sex and Gender in Research

Cell line source(s)	Tumor lines were derived from tumor biopsies in-house. T2 cells were obtained from ATCC (CRL-1992) as well as HEK293T cells (CRL-3216). TCR/CD3 Jurkat cells (NFAT) were obtained from Promega (cat# J131A, Promega Academic Access Program).
Authentication	T2 cells and HEK293 cells were authenticated by ATCC, using STR profiling (www.atcc.org/products/CRL-1992 and www.atcc.org/products/CRL-3216, respectively). TCR/CD3 Jurkat were not authenticated.
Mycoplasma contamination	Before each study, cells were tested negative for mycoplasma by PCR in-house.
Commonly misidentified lines (See ICLAC register)	No misidentified cell lines were used.

Animals and other research organisms

Policy information about studies involving animals; ARRIVE guidelines recommended for reporting animal research, and Sex and Gender in Research

Laboratory animals	IL2-NOG mice were obtained from Taconic Biosciences and maintained in a conventional animal facility at the University of Lausanne under specific pathogen-free status. Housing conditions of mice were the following: alternating dark/light cycles of 12h, humidity 55% (+/- 10%) and temperature 22°C (+/- 1°C). Six- to nine-week old female were used in this study.
Wild animals	No wild animals were used in this study.

Reporting on sex	Sex of animals was not considered in this study. However, as patented by Taconic Biosciences, we could only one gender. As we studied ovarian cancer samples, we used only females.
Field-collected samples	No field-collected samples were used in this study.
Ethics oversight	This study was approved by the Veterinary Authority of the Canton de Vaud (under licenses VD3387 and VD3746) and performed in accordance with Swiss Ethical Guidelines.

Note that full information on the approval of the study protocol must also be provided in the manuscript.

Flow Cytometry

Plots

Confirm that:

- [x] The axis labels state the marker and fluorochrome used (e.g. CD4-FITC).
- [x] The axis scales are clearly visible. Include numbers along axes only for bottom left plot of group (a 'group' is an analysis of identical markers).
- [x] All plots are contour plots with outliers or pseudocolor plots.
- [x] A numerical value for number of cells or percentage (with statistics) is provided.

Methodology

Sample preparation	Cells from culture media were harvested and stained in FACS buffer (PBS 1x, sodium azide 0.05%, EDTA 5 mM) at 4°C with first tetramers, second with antibodies and finally with dead cell markers. A more detailed protocol is available within the method section of this manuscript.
Instrument	Data were acquired on LSRII, LSRFortessa (BDBiosciences) or IntelliCyt iQue Scener Plus (Bucher Biotec).
Software	FlowJo version 10.5.3 and DIVA version 9.0 were used to analyse and acquire the data.
Cell population abundance	Tetramer-sorted bulk or single clones obtained by limiting dilution were checked by tetramer staining before being used in any experiment.
Gating strategy	In all flow cytometry experiment, doublets were excluded by gating on FSC-A/FSC-H and SSC-A/SSC-H plots. Live cells were determined by exclusion from positive Live/Dead stained cells.

- [x] Tick this box to confirm that a figure exemplifying the gating strategy is provided in the Supplementary Information.
